# Protein-Binding Microarray Analysis of Tumor Suppressor AP2α Target Gene Specificity

**DOI:** 10.1371/journal.pone.0022895

**Published:** 2011-08-18

**Authors:** Jan Kerschgens, Stéphanie Renaud, Frédéric Schütz, Luigino Grasso, Tanja Egener-Kuhn, Jean-François Delaloye, Hans-Anton Lehr, Horst Vogel, Nicolas Mermod

**Affiliations:** 1 Institute of Biotechnology, University of Lausanne, Lausanne, Switzerland; 2 Swiss Institute of Bioinformatics, University of Lausanne, Lausanne, Switzerland; 3 Laboratory of Physical Chemistry of Polymers and Membranes, École Polytechnique Fédérale de Lausanne, Lausanne, Switzerland; 4 National Competence Center for Biomedical Imaging, École Polytechnique Fédérale de Lausanne, Lausanne, Switzerland; 5 Centre Hospitalier Universitaire Vaudois, Lausanne, Switzerland; Université Paris-Diderot, France

## Abstract

Cheap and massively parallel methods to assess the DNA-binding specificity of transcription factors are actively sought, given their prominent regulatory role in cellular processes and diseases. Here we evaluated the use of protein-binding microarrays (PBM) to probe the association of the tumor suppressor AP2α with 6000 human genomic DNA regulatory sequences. We show that the PBM provides accurate relative binding affinities when compared to quantitative surface plasmon resonance assays. A PBM-based study of human healthy and breast tumor tissue extracts allowed the identification of previously unknown AP2α target genes and it revealed genes whose direct or indirect interactions with AP2α are affected in the diseased tissues. AP2α binding and regulation was confirmed experimentally in human carcinoma cells for novel target genes involved in tumor progression and resistance to chemotherapeutics, providing a molecular interpretation of AP2α role in cancer chemoresistance. Overall, we conclude that this approach provides quantitative and accurate assays of the specificity and activity of tumor suppressor and oncogenic proteins in clinical samples, interfacing genomic and proteomic assays.

## Introduction

Since the complete sequencing of the human genome in 2001 [1], [2], a wealth of DNA sequences has been available via online databases [1], [3], [4], [5], [6], [7]. The vast majority of the sequences are intergenic or intronic, which may provide the platform for the concerted action of DNA-binding regulatory proteins and chromatin constituents. Knowledge of the integration of the multitude of specific transcription factor binding may lay the foundation for a system-wide understanding of fundamental multicellular processes like development and growth, and for more comprehensive descriptions of diseases that are linked to gene expression misregulation. Human diseases like cancer have often been linked to the improper interplay of proteins involved in the transcriptional control of cells and tissues, as illustrated by the prominent role of oncogenes in regulating gene transcription and chromatin structure [8], [9].

Several laboratory techniques have been devised for large scale identification of transcription factor target sites, either *in vitro* or using cellular assays [10]. One such assay relies on protein-binding microarrays (PBM) that bear immobilized double-stranded DNA molecules to which the binding of regulatory proteins can be probed. PBMs have been prominently used for the assignment of the binding specificities of purified transcription factors [10], [11], [12], [13], [14], [15]. A Recent studies also demonstrated that PBMs can be used to assess the DNA-binding specificity of transcription factors from cell extracts [10], [16]. Subsequent computational analysis of PBM-generated data allows the computing of protein-specific DNA-binding weight matrices, which can be used to scan genomic sequences to identify new putative binding sites and transcriptional pathways, as exemplified by those formed by the Hox proteins and developmentally regulated genes [17]. However, the actual binding of the transcription factors to the predicted site must be confirmed experimentally, as it may be occluded by chromatin or DNA modification or by other proteins binding overlapping DNA sequences, while synergistic binding may occur on non-canonical sites that are not detected by *in silico* predictions.

Activating protein 2 alpha (AP2α) is a transcription factor whose binding sites were first discovered in cellular and viral *cis*-regulatory sequences and gene promoters [17], [18], [19]. It belongs to the TFAP2 family of sequence-specific DNA-binding polypeptides that share a highly conserved basic helix–span–helix DNA-binding and dimerization domain at their C-terminus, and a less conserved N-terminal proline-rich and glutamine-rich transcriptional regulatory domain. The various TFAP2 isoforms, namely AP-2α, AP-2β, AP-2γ, AP-2δ, and AP-2ε in humans and rodents, form homo and heterodimers that recognize GC-rich palindromic DNA sequences related to the 5′-GCCN_3–4_GGC-3′ consensus sequence [20], [21].

AP2α biological function stretches from the regulation of neural crest formation during mice development to a proposed role in the mitochondrial pathways leading to apoptosis [22], [23], [24]. Cloning of AP2α coding sequence has allowed the identification of protein-interaction partners and of a small set of potential target genes [25], [26], [27], [28]. Interestingly, AP2α DNA-binding specificity was reported to be modulated by synergistic or antagonistic interactions with other DNA binding proteins present in human tumor cells, and changes in these interactions was associated to tumor progression [21], [24], [29]. At present, a system-wide identification of its direct and indirect target genes is not available, despite growing interest raised by its action as a tumor suppressor or oncogene and its implication in cancer progression and resistance to therapeutics.

PBMs have so far been used mostly to assess interactions to short synthetic DNA sequences, for the modeling of the DNA sequence specificity of transcription factors. Here we show that PBMs can be used to perform large-scale assays of the interaction of regulatory proteins from crude cellular extracts with long genomic fragments such as promoters and enhancers. Assay of approximately 6000 human genomic sequences allowed an *ab initio* assignment of the target gene specificity of the AP2α tumor suppressor, as a purified protein as well as from healthy and cancer breast tissues from patients. Several target genes were validated in cell-based assays. The PBM-based approach may thus allow the identification of previously unknown target genes of tumor suppressors in cancer cells, and it provides novel markers of cancer progression at the interface of proteomics and genomics.

## Results

### AP2α binding assay on 6000 human genomic sequences

**Figure 1:**
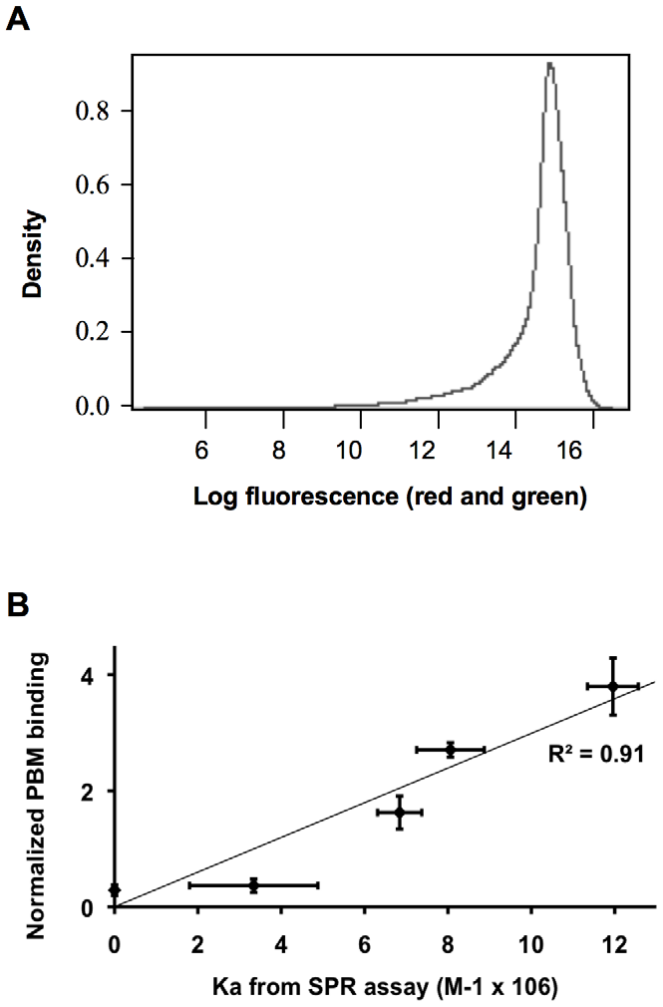
Analysis of AP2α binding to PBM DNA sequences. (A) Distribution of the measured values on each slide (red and green channel) after normalization. Values on the X axis represent the log_2_ of green or red fluorescences. Following value normalization, tracings obtained from the two channels are superimposed. (B) Association of protein-binding microarrays (PBM) and SPR-estimated binding affinities. Five different sequences of 252 bp length were analyzed for AP2α binding using PBM and SPR. The resulting relative affinity measurement averages of 3 independent assays each were fitted by a linear regression.

We first aimed at identifying potential target genes of the AP2α oncogene/tumor suppressor protein by assessing its binding to double-stranded DNA sequences immobilized on a microarray. We used a collection of human genomic DNA sequences that was originally devised for ChIP-chip assays [30]. This microarray contains approximately 6000 promoter and intergenic regulatory human DNA sequences of approximately 1000 bp length on average (hu6k microarray) that were labeled with the Cy5 fluorophore. Bacterially expressed and purified AP2α protein was incubated with the microarray, and its binding to particular sequences was assessed using Cy3-labeled fluorescent antibodies and microarray scanning. Dye bias was removed by the Loess normalization, and the data was normalized first within each slide and then among three independent slides, to remove intra- or inter-slide biases (see Materials and Methods section). The distribution of the normalized binding values is shown in Figure 1A (see Table S1 for the complete data set and statistical analysis). A high reproducibility was observed between individual assays, with the majority of the coefficient of variation (CV) values under 5%, and most under 10%, for both the red (Cy5 DNA labeling, Figure S1A) and the green (Cy3 protein labeling, Figure S1B) channels.

As PBMs rely on immobilized DNA molecules that reach high local concentrations at the surface of microarrays, slow or undetectable off-rates may occur [31]. Thus, whether a thermodynamic binding equilibrium is reached during the assay, and consequently whether accurate affinity values may be deduced from PBM data has remained unclear. We addressed this issue by assaying a set of dsDNA molecules chosen to have a wide range of affinities, as predicted using a previously described AP2α hidden Markov model of AP2-binding specificity (Figure S2), and as assessed experimentally [32]. The DNAs were immobilized on Biacore sensor chips using an oriented biotin-streptavin crosslink, and the binding constants of purified AP2α were determined by surface plasmon resonance (SPR) for low (E18), medium (E25, E33) and high affinity (E38 and E58) DNA sequences. In parallel, these sequences were also spotted on a small scale PBM, and AP2α binding was assessed as described above. Affinity values determined by SPR were found to correspond well to the PBM-based and weight-matrix-estimated affinities of AP2α, with a coefficient of correlation of 91% (Figure 1B and Figure S3).

### AP2α target genes network analysis

**Figure 2:**
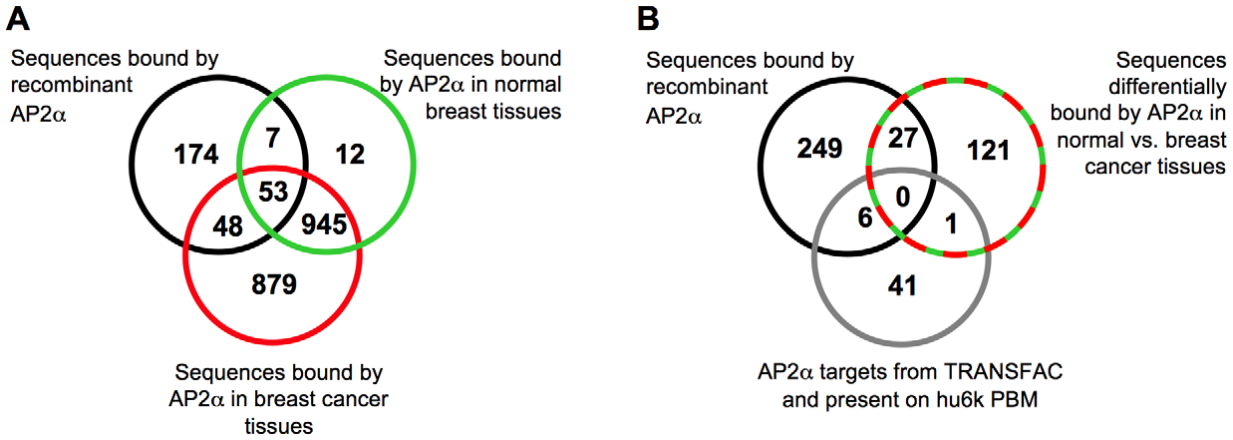
Venn diagrams depicting the overlap between the sequences most potently bound by AP2α from different sources. (A) The 282 sequences found to be significantly bound by recombinant AP2α on hu6k PBM were compared to 1017 sequences bound by AP2α from healthy breast tissues and 1925 sequences from breast cancer tissues. (B) The 282 sequences significantly bound by recombinant AP2α were compared to 48 sequences bound by AP2α from the TRANSFAC database and to the 149 sequences differentially bound by AP2α when comparing healthy to cancer breast tissues. The comparison is as in panel A, except that the datasets obtained from tissue extracts were obtained using a significance cut-off of p<0.01.

**Figure 3:**
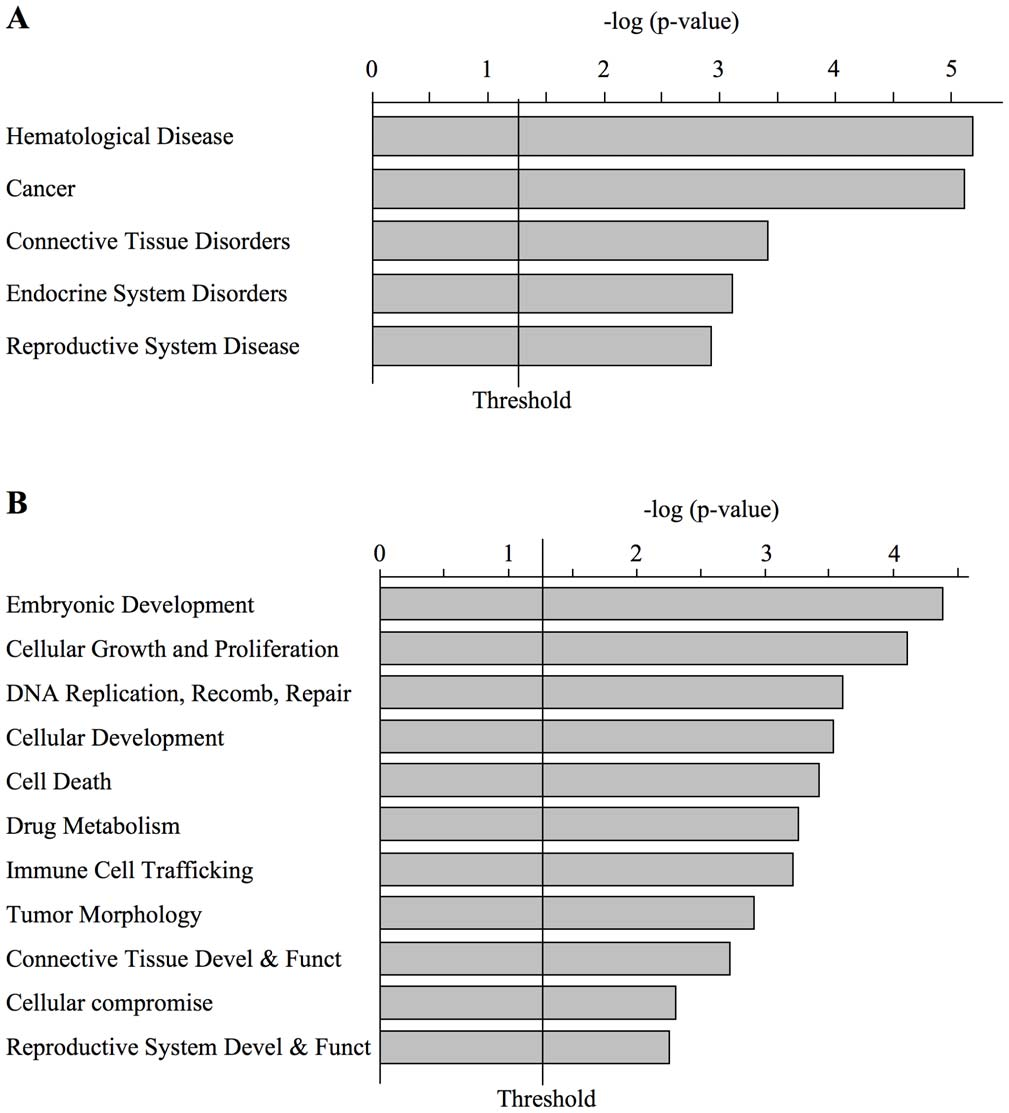
Functional analysis of AP2α target genes. Most prominent functional categories of diseases and pathological disorders (A), or of biological functions (B), associated with 272 potential AP2αtarget genes (PBM binding values, p<0.05). The threshold significance line indicates a value of −log(p-value) greater than 1.25.

After validating the PBM-based interactions *in vitro*, we assessed globally the functional significance of potential AP2α target genes. A statistical analysis yielded 282 DNA sequences that were significantly bound by AP2α (see Table S1 for a complete data set). In order to assess if some of these hits may correspond to previously identified AP2α targets, we compared the 282 PBM sequences with AP2α target genes as listed on the TRANSFAC database (http://www.biobase-international.com). 49 genes represented on the hu6k microarray were also listed in the TRANSFAC dataset. Among these 49 sequences, 6 were directly bound by the recombinant AP2α protein on the PBM (Figure 2). This first comparison indicated that the analysis of the binding of recombinant AP2α to the PBM sequences can reveal functionally relevant target genes. The biological significance of these 282 AP2α-bound sequences was assessed using the Ingenuity Pathway Analysis software, among which 175 sequences could be associated with one or several biological functions or diseases. Target genes associated with cancer and hematological diseases featured prominently (Figure 3A). A similar analysis of functions pertaining to physiological systems and biological functions revealed an enrichment of genes associated with development, cellular growth and proliferation, or cell death (Figure 3B).

**Figure 4:**
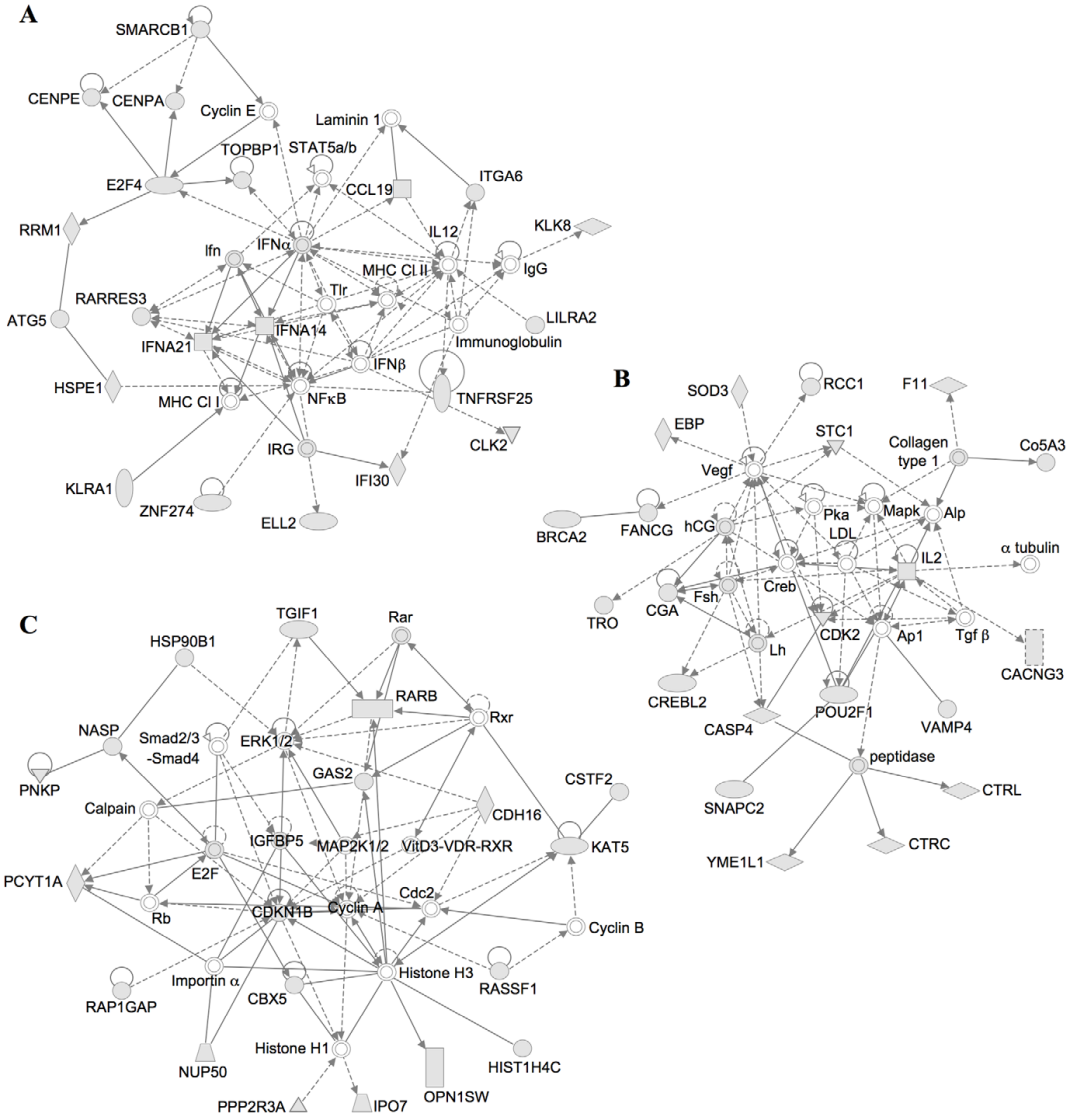
Network diagrams illustrating prominent regulatory relationships among AP2α-bound genes. Grey-filled shapes represent genes significantly bound by recombinant AP2α on the PBM. Main functions related as these networks, as constructed and classified by the Ingenuity Analysis Software are cellular development, cell-to-cell signaling and interaction, and embryonic development (A), cell death, cancer and genetic disorder (B), or cancer, cell cycle and embryonic development (C).

Network analysis was also performed to provide a graphical representation of the biological inter-relationships of AP2α-bound genes. This led to the identification of twenty-one networks whose gene nodes preferentially associate with AP2α (Table S2). Among the five most significant networks, three of them feature cancer and/or development prominently. One represents the interaction of cell-to-cell signaling intermediates with regulators of the cell cycle and of embryonic development (Figure 4A). Another network features the interactions of cell death regulators with cancer, while the third one illustrates the connection of cancer-linked proteins with the cell cycle and embryonic development (Figure 4B and 4C). Other networks whose gene nodes are often bound by AP2α were linked with developmental disorders and cellular development and morphology (Figure S4). Overall, we conclude that AP2α associates preferentially with developmental regulator and cancer-associated genes. Taken together, these analyses strongly supports the role of AP2α in developmental regulation, as illustrated by the severe dysmorphogenesis of numerous tissues and organs observed in AP2α-defficient mice, as well as its role as a tumor suppressor or as an oncogene [20], [21].

### Cell-based validation of potential AP2α target DNA sequences

**Figure 5:**
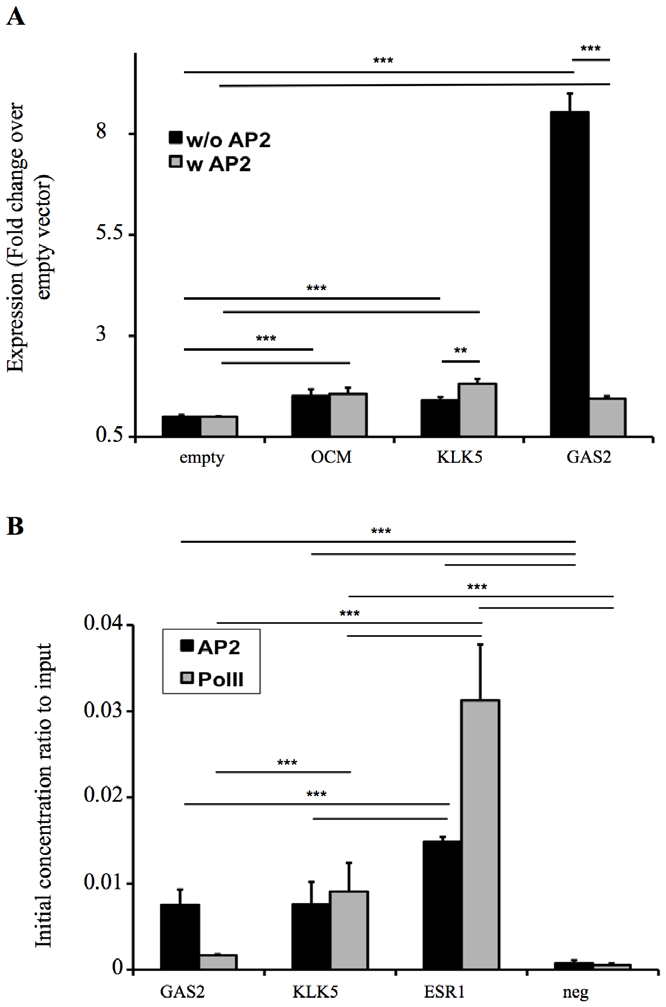
Functional validation of AP2α binding sites selected from PBM and weight matrix-based predictions. A) Genomic sequences corresponding to the OCM and GAS2 genes were selected on the basis of low and high binding potential, respectively, from the PBM dataset, while the KLK5 sequence was selected as an AP2α-bound promoter by weight matrix-based predictions with a score of 39 (medium affinity). SW480 cells were transiently co-transfected with reporter constructs containing these ∼900 bp regulatory sequences genomic sequences inserted upstream of a minimal promoter and eGFP sequence, and with either an AP2α expression vector or with the corresponding empty control vector. eGFP fluorescence was quantified by cytofluorometry. Graphs show the fold change of eGFP expression in absence or presence of AP2α as normalized to the fluorescence level obtained from the cotransfection of the eGFP reporter plasmids alone (n = 4, ***: p<0.001, **: p<0.005; two-tailed, two-sample equal variance t-test). B) ChIP-qPCR analysis of *in vivo* AP2α binding was performed using KM12C colon cancer cells that constitutively expresses AP2α [41]. The ∼900 bp sequences used in the eGFP reporter assay of panel A where searched for AP2α binding motifs utilizing the prediction weight matrix shown in Fig. S2, and qPCR primers were designed for the quantification of the assigned regions. A known AP2α binding site on the Estrogen Receptor 1 gene (ESR1) promoter serves as positive control, while an intergenic sequence upstream of the KLK5 gene was used as negative control. The abundance of RNA polymerase II indicates promoter occupancy by the transcriptional machinery. The initial concentration ratio of IP sample to sample Input were calculated as previously described [71]. Values obtained from the immunoprecipitated samples were multiplied by a factor of 100 before plotting.

Several promoters were selected for experimental validation from the PBM and *in silico*-identified potential AP2α targets. This was performed on the intergenic sequence of the Growth arrest-specific protein 2 (GAS2) gene, an activator of p53-mediated apoptosis [33], [34], as well as the kallikrein 5 (KLK-5) gene, a secreted protease involved in cancer progression [35], [36], [37], [38]. The promoter sequence of the parvalbumine gene (OCM gene) was selected as a negative control, on the basis of its low interaction with AP2α on the PBM [39], [40]. These sequences were inserted upstream of a minimal promoter and the eGFP sequence (Table S3), and they were transiently transfected into SW480 colon cancer cell line, as these cells display low endogenous AP2α levels [41]. The OCM promoter sequence mediated a low level of eGFP expression that was not affected by the co-transfection of an AP2α expression vector, consistently with the lack of significant interaction with the transcription factor in the PBM analysis (Figure 5A). The intergenic GAS2 regulatory sequence increased eGFP expression from the minimal promoter by approximately 8-fold in the absence of AP2α, but this effect was abbrogated by the expression of AP2α. The KLK-5 sequence mediated a low expression comparable to that conferred by the OCM sequence, and AP2α expression mediated a small but nevetherless significant activation, in agreement with the occurrence of a AP2α binding site. Similar results were obtained from similar experiments performed using a hepatocarcinoma cell line (Figure S5). We concluded that the novel AP2α target genes likely represent functional interactions, in that AP2α expression could mediate either a repressive (GAS2) or activating (KLK5) effect on these target promoters.

Next, we assessed whether these AP2α interactions may take place in the cell in the context of chromatin. AP2α binding sites having the highest score computed from the weight matrix prediction algorithm were localized on the DNA sequences imobilized on the PBM, and the corresponding genomic sequences were assayed for AP2α–interaction by chromatin immunoprecipitation-quantitative PCR (ChIP-qPCR) using a colon cancer cell line that constitutively expresses this transcription factor [41]. AP2α was indeed found to interact with the predicted sites on the GAS2 and on KLK-5 upstream regulatory sequences in the cancer cells as well as to the AP2α binding site in the promoter of the estrogene receptor gene used as a positive control (ESR1, Figure 5B). RNA polymerase II was found to bind to the KLK-5 promoter but not to that of GAS2, as expected from the activating and repressive role of AP2α seen in transfection studies, respectively. This indicated that the PBM and *in silico* analysis allowed the identification of functionally relevant target genes.

### Association of AP2α from cancer tissue to human genomic sequences

It has been proposed that AP2α DNA-binding specificity is modulated by synergistic or antagonistic interactions with other DNA binding proteins present in human tumor cells, and that changes in these interactions may lead to tumor progression [21], [24], [29]. AP2α association was therefore also assessed on the 6000 human genomic regulatory sequences in conditions where these competitive or synergistic interactions may take place. Nuclear extracts were generated from 4 healthy breast tissues and 8 breast cancer biopsies, and they were applied to hu6k microarrays to specifically detect AP2α binding [32]. Using nuclear protein extracts from normal or cancerous breast tissues, 1017 and 1925 sequences were identified as potential AP2α targets (See Tables S4 and S5 for complete datasets and statistical analysis).

Among the 998 target sequences obtained from both the healthy breast tissue and the breast cancer protein extracts using the log fold change (protein/DNA) as endpoint, 53 also associated with the purified protein (Figure 2A). These likely corresponds to direct binding targets of AP2α as exemplified by chorionic gonadotropin alpha promoter (CGA), which is a well characterized functional and binding target of AP2α in tumor cells [42]. The synergistic or antagonistic binding of AP2α with numerous other DNA-binding proteins may explain different gene occurrence in the various datasets. For instance, interactions observed from nuclear extracts of healthy and/or cancerous tissues, but not from purified AP2α may result from its ability to piggy-back other DNA-binding proteins such as YY1, Sp1 or p53 [43], [44], [45].

Using a position weight matrix (PWM) prediction algorithm for AP2α binding sites [32], we tested the 50 sequences most prominently bound by AP2 from the PBM data sets generated with recombinant AP2α and with the normal and tumor tissue extracts. We found that the occurrence of AP2α predicted binding sites was 1.5 fold lower in sequences obtained using normal tissue extracts as compared to those obtained with the recombinant AP2α (Table S6). This result thus supports the interpretation of an indirect binding of AP2α occurring from tissues extract and the proposed ability of AP2α to interact with other proteins to bind its target genes in the context of normal cells. Interestingly, there was a higher percentage of genes with a predicted AP2α binding sites when analyzing the dataset obtained with the cancer extracts (Table S6). This likely reflects a greater ability of AP2α to bind DNA directly in cancer cells as compared to normal cells, as also indicated by the high number of targets commons to recombinant AP2α and to the cancer extracts (Figure 2A). Thus, oncogenic transformation may be accompanied by the release of AP-2 from interactions with proteins that hinder its ability to directly interact with its cognate recognition sequence. Indeed, a higher number of sequences significantly bound by AP2 were obtained from the cancer extract relative to the healthy tissue (Figure 2A). When similarly using a PWM algorithm that predict p53 binding sites (p53mh, [46]), little difference was found between hits obtained from the cancer and normal tissue extracts (Table S6). However, when genes that have a low PWM score for AP2α binding and a high score for p53 binding were extracted from these datasets, as genes that AP2α might indirectly bind by piggybacking p53, a higher proportion was bound by AP-2 from the normal tissue extracts. This implied that such genes may bind the transcription factor indirectly, via AP-2's ability to associate with p53, but that this ability may be lost or decreased upon oncogenic transformation. Indeed, some of these genes are known regulatory targets of p53, as exemplified by the REPRIMO, GDF9 and TLR3 genes [47], [48], [49]. Two other such genes encode the matrix metalloproteinase 2 (MMP2) and Rad51, where AP2α DNA binding and regulation was shown experimentally to require p53. Consistently, these genes were not recognized by purified AP2α recombinant protein but interaction was only observed when using nuclear extract (Tables S1, S4, S5). Thus, the PBM results suggest that indirect interactions of AP2α are much more widespread than previously known and that oncogenic transformation is accompanied by a change in AP2α target gene specificity mediated in part by the modulation of these indirect interactions. In this respect, novel AP2-bound genes from cellular datasets feature prominent cell cycle-related regulatory targets of the p53 and Rb tumor suppressors such as the E2F and cyclin gene families [50].

**Figure 6:**
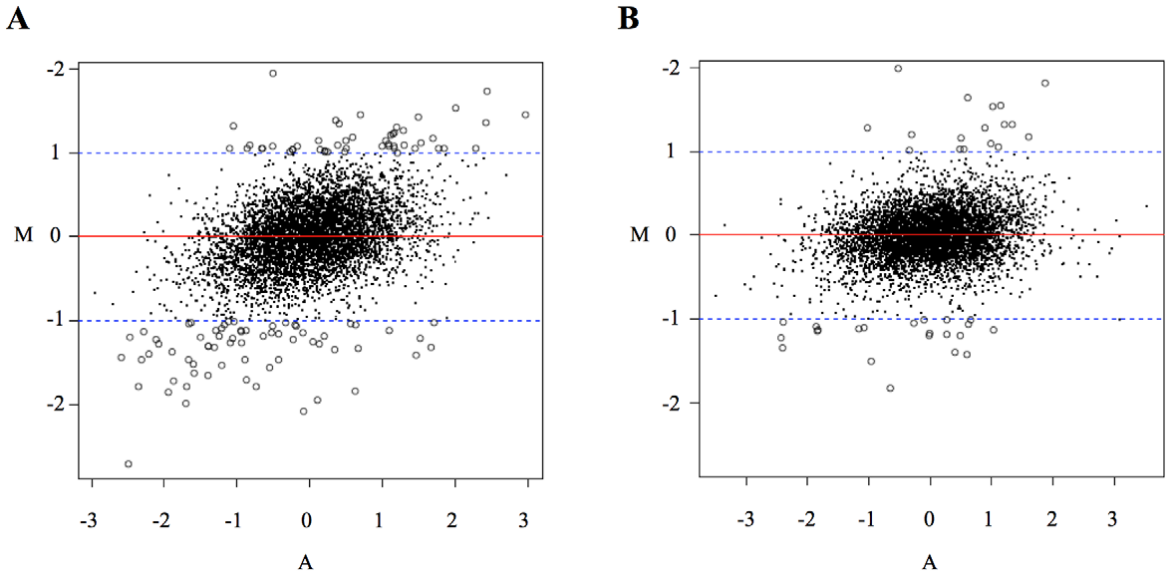
PBM analysis of AP2α extracted from healthy or tumor tissue samples. (A) Result of a comparison between 4 normal samples and 4 cancer samples, in the form of an MA-plot. The X-axis (A values) shows the average of the log ratios (protein/DNA) of binding from normal and cancer extracts to each target DNA sequence, and it thus displays the average AP2 binding value to each gene. The Y-axis (M values) represents the averaged difference of log ratios (protein/DNA) between the normal and the cancer samples, and thus represent differential binding when comparing healthy and diseased tissues. Potential differential binding was defined as an absolute value of M>1, as shown with dashed lines, corresponding to a 2-fold difference between cancer and normal tissues. 149 genes were selected using this cut-off. Circles indicate statistically significant differential binding. (B) Same data for a comparison between two sets of 4 randomly selected cancer samples; 52 genes were selected using the same criteria as for panel (A).

Comparison of AP2 binding specificity from normal and tumor tissues yielded generally correlated results, as many genes that were bound by AP2α in the healthy tissue extract were also bound using tumor extracts (Figure 2A). The higher number of genes bound from the tumor extracts can be attributed to the combined effects of differences in the activity of AP2α and of the proteins it synergizes or antagonizes with. Consistently, cancer-associated genes that had not been previously associated to AP-2 were identified in the tumor extracts datasets, as for instance the breast cancer susceptibility gene 2 (BRCA2) and the cyclin-dependent kinase 2 gene. Comparison of the binding strength of regulatory sequences detected using the two types of extract yielded 149 sequences that were differentially bound by AP2α (Figure 2B and Figure 6A). When a similar comparison was performed between 2 sets of 4 randomly selected breast cancer extracts, to assess the experimental noise, 52 differentially bound sequences were obtained (Figure 6B). Differences between the two sets of tumors may reflect the known heterogeneity of breast tumors [21]. Nevertheless, the nearly three-fold higher number of differentially bound genes from healthy versus breast tumors tissue comparisons indicated that AP2α specificity differs much more between normal and cancerous tissues than among individual tumor types. One of the genes bound solely from the cancer biopsy extract was found to encode Bcl2, which is known to be down-regulated by AP2α to provoke tumor cell apoptosis upon chemotherapy [23]. Functional analysis of networks associated with genes differentially bound by AP2α in normal and tumor extracts showed that genes are involved in genetic disorders and cancer, but also in reproductive system disease (Table S7). Taken together, these results imply that the PBM-based approach may be used to detect bona fide direct and indirect targets of AP2 as well as reveal novel ones, and that it may differentiate healthy from cancerous breast tissues. They also support previous proposals that AP2α DNA binding may be subjected to antagonistic or synergistic interactions with numerous other nuclear proteins in tumor cells [21], [51], [52].

## Discussion

In this study, we evaluated whether double-stranded DNA fragments bearing whole promoters and regulatory sequences and immobilized on a PBM may be used to identify the target genes of an oncogenic transcription factor. We found that binding values obtained from the PBM correlated well with the affinities determined by surface plasmon resonance or computed with a weight matrix. This indicated that PBMs provide reliable estimations of the binding strength. Genomic fragments that bind AP2α on the PBM and/or *in silico* were found to mediate AP2α-regulated expression in transfection assays. Occupancy of the AP2α binding sites within the native chromatin structure of tumor cell lines was confirmed experimentally. This also indicated that valid target genes may be identified by combining these PBM and modeling approaches. Thus, the increase in non-specific background binding to the long DNA molecules, as resulting from the use of relatively long promoter and enhancer sequences, did not mask sequence-specific functional interactions.

*In vivo*, an additional level of complexity arises from the chromatin structure, which may shield the DNA from protein binding. Thus, a high binding potential *in vitro* or *in silico* cannot provide definitive evidence that a putative binding site will be occupied in any given cell type. Furthermore, binding may be occluded by other transcription factors that interact with overlapping sites on the promoter, or conversely, protein association may allow interactions to non-canonical sites. The relative contributions of chromatin and of other transcription factors to the actual binding site occupancy *in vivo* remains difficult to assess for eukaryotic transcription factors, as large-scale assays of promoter binding occupancy with chromatin alone, or with competing or synergizing transcription factors but without chromatin, have not been available.

The finding that the most prominent functional feature of AP2α target genes relates to cancer was validated experimentally for two newly identified AP2α target genes. Kallikrein 5 is a member of the kallikrein family of extracellular proteases that includes the prostate-specific antigen, and it is currently emerging as some of the most prominent biomarkers of tumor progression for various types of cancers [53], [54], [55]. The growth-arrest specific 2 (GAS2) protein modulates cell susceptibility to p53-dependent apoptosis upon chemotherapy [33], [34]. The finding of an AP2α-mediated regulation of both genes may thus provide a molecular mechanism for its proposed role in tumor progression and resistance to chemotherapy.

Interestingly, we found that promoter-binding by AP2α from total cell nucleus extracts differs significantly from that observed with the purified protein. Because nuclear extracts contain regulatory proteins such as transcription factors but do not mediate nucleosome deposition, differences in the binding patterns obtained from healthy and cancer tissues imply that AP2α binding is regulated by other regulatory proteins present in the extracts. This finding correlates well with previous observations of the synergistic or antagonistic interactions of AP2α with other transcription factors and oncogenes such as p53, Rb and c-Myc [21], [51], [52]. The recruitment of AP2α at p53-binding sites and the resulting synergistic regulation of p53 target promoters has been associated to the tumor suppressor effects of the two transcription factors [21], [51], [52]. Interaction of AP2 from the crude extracts was observed with two such AP2α and p53-regulated genes presented on the PBM, those of the matrix metalloproteinase 2 and Rad51 genes [43], [44], [45], while these interactions were not seen with the purified protein. In addition to the alterations of AP2 DNA binding specificity by piggybacking effects, the formation of heterodimers of AP2α with other AP2 species may also alter its interaction with target genes. Although, the DNA binding specificity of the various isoforms are generally considered to be similar, the possibility of an altered interaction of particular heteromultimers from the tissue extracts but not from the purified AP2α cannot be excluded. For instance, a preferential interactions of the AP2α/AP2γ or AP2β/AP2γ dimmers has been proposed to occur on the KAI1 prostate cancer metastasis suppressor gene [56]. Thus, numerous types of interactions may concur to modulate the interaction of cellular AP2α with its target genes and may correspondingly affect its interaction with the PBM.

Overall, we conclude that the use of genomic DNA-bearing PBMs and unfractionated cell nuclear extracts can provide so far unavailable data allowing (i) to distinguish transcription factor cooperative or antagonistic effects from those elicited by chromatin, and (ii) to interpret some of the discrepancies noted between cellular assays such as ChIP and assays of purified components *in vitro* or from *in silico* predictions. PBMs can thus complement other microarray-based assays of disease related proteins, as may be needed to achieve more efficient integrated molecular diagnostics of cancer.

## Materials and Methods

### Ethics statement

Written informed consent obtained from patients undergoing tumor biopsies and the protocol for use of human tissue samples were in accordance to the guidelines of -and approved by- the ethical committee of the University Hospital of Lausanne (Switzerland).

### Tissues and cell lines

Five breast cancer frozen tissues were obtained from the Tissue Bank of the University Hospital of Lausanne. Five normal breast tissues from reduction surgeries were obtained from Dr. Brisken C. Laboratory (ISREC, Lausanne). All tissue samples were histologically examined by a pathologist (H.A.L.).

SW480, humancolorectal adenocarcinoma hybridoma (ATTC line number CCL-228), was cultured according to the supplier's instructions. This cell line is documented to express little or no significant AP2 levels [22], [23], [24]. AP2α-expressing KM12C, human colon cancer cell line [57], was grown in DMEM (ATCC catalogue number 30-2002) supplemented with 10% FBS.

### Preparation of proteins and DNA reagents

Full-length human TFAP2α protein was overexpressed as a GST-fusion protein in *Echerichia coli W3110* utilizing the previously described expression vector pGEX-AP2 following the manufacturer's instructions [32]. The GST-AP2α fusion protein was purified by column affinity chromatography on Glutathione Sepharose 4B (Amersham Biosciences). Glycerol was added to the final concentration of 10%, and aliquots were shock frozen in liquid nitrogen and stored at −80°C.

Plasmids bearing standard DNA sequences for AP2α binding served as template for PCR-mediated DNA amplification [32]. Double stranded 252 bp sequences for biotin-streptavidin coupling to biacore sensor chips or spotting on microarrays were amplified by polymerase chain reaction (PCR) utilizing 5′-biotin-CGA CGT TGT AAA ACG ACG GC-3′ and 5′-TGA CCA TGA TTA CGC CAA GC-3′ primers, as described previously [32]. DNA fragments were purified form 1×TAE agarose gels (1.8% w/v) utilizing the ‘PCR and Gel purification kit’ (Promega) and DNA concentration was quantified with a Nanodrop ND-1000 spectrophotometer (Witeg AG).

### Preparation of total protein extracts from tissue samples

Tissue sample sections of 30 µm were thawed on ice in a solution of 1× PBS (10 mM pH 7.4 Phosphate, 137 mM NaCl, 2.7 mM KCL) containing a cocktail of protease inhibitor (Roche complete protease inhibitor cocktail tablets, Roche) followed by centrifugation of the tissue fragments at 4°C for 5 min at 3500 rpm. Pellets were recovered into 250 µL Low Salt Lysis Buffer (10 mM Tris pH 7.8, 1.5 mM MgCl_2_, 10 mM KCl) and incubation allowed on ice for 15 min. Cells were lysed by addition of 0.5 µL NP-40 [0.02% w/v] and mixed by vortexing for 5 s. Nuclei were harvested by centrifugation for 5 min at 3500 rpm, 4°C and the pellet washed with 300 µL Low Salt Lysis Buffer. After an additional centrifugation step (3500 rpm at 4°C for 5 min) the supernatant was taken off and the pelleted nuclei resuspended in 25 µL of High Salt Lysis Buffer (10 mM Tris pH 7.8, 1.5 mM MgCl_2_, 10 mM KCl, 400 mM NaCl, NP-40 [3% w/v]). Samples were mixed by vortexing for 5 s and incubated on ice for 30 min with vortexing every 5–10 min in order to allow extraction of nuclear proteins. The supernatant containing the nuclear proteins was transferred into new coded tubes and diluted by adding 25 µL of Low Salt Buffer to bring the final NaCl concentration to 200 mM and immediately frozen in liquid nitrogen.

### Protein-binding microarray assay

Spotting of custom microarrays was done as described previously [32]. In short, DNA concentrations were adjusted to 170 ng/µL unless stated otherwise and mixed 1∶1 with 2× spotting buffer (6× SSC; 3 M betaine). Each sequence was spotted 20 times on different positions on the array slide. Spotting was carried out with an ESI arrayer (BioRad) at 50–55% humidity and 23°C onto Nexterion aldehyde slides (Schott).

Custom-made PBMs, or the hu6k microarrays containing approximately 6000 human genomic sequences of similar length (980 to 1000 bp; Aviva Systems Biology, San Diego, USA; [30]) were treated prior to use as follows: slides were washed twice for 2 min with 1×TEN (40 mM Tris-HCL [pH 7,5], 1 mM EDTA, 150 mM NaCl), then reduction of aldehydes was performed in the reduction solution (0.25% NaBH4 (w/v) in PBS 1×/EtOH 25%) for 10 min, followed by two washes of 2 min with 1×TEN. Slides were then blocked in the blocking solution for 1 h (5% skimmed milk in PBS 1×, 0.05% Tween-20) followed by two final washes for 2 min with 1×TEN. The DNA on the arrays was end-terminally labeled following the protocol described here: pre-incubation of slides with 1×PBS containing 100 µg/mL adenosine monophosphate (AMP) was followed by application of the DNA labeling mix (1× NEB buffer 4, 100 µg/mL AMP, 0.1 mM CoCL_2_, 0.2 µM ddNTP-Cyanine5 [Perkin Elmer], 0.02 U/µL TdT [NEB]). After incubation for 1.5 h at 37°C in the absence of light, slides were washed twice for 2 min in 1×TEN and spun dry.

AP2-binding reaction mix (2.5 mM Tris-Hcl pH 7.9, 15 mM KCl, 1 mM MgCl_2_, 0.025 mM EDTA, 12.5 µg/ml BSA, 0.05% NP40 2% [w/v] skimmed milk, 0.015 µg/µL poly-dIdC, 0.15 ng/µL purified GST-AP2α or 100 to 1000 ng/ml of total proteins from nuclear cell extract) was applied onto each array slide and incubated at room temperature (RT) in the absence of light for 1 h, as described previously [14], [32]. Slides were washed 5×3 min with 1× PBS supplemented with 1% (v/v) Tween 20, 3×5 min with 1× PBS containing 0.01% (v/v) Triton-X-100 and spun dry. Next, the primary antibody (AP2α (C-18):sc-184, Santa Cruz Biotechnologies) was applied in 1× PBS supplemented with 2% skimmed milk, and incubated for 1 h at RT in the absence of light. Slides were washed 3×5 min with 1× PBS with 0.05% (v/v) Tween 20, 3×5 min with 1× PBS with 0.01% (v/v) Triton-X-100, before incubating with the secondary antibody (Cy3-labeled Alexa Fluor 546 goat anti-rabbit IgG, Molecular Probes) in 1× PBS supplemented with 2% (w/v) skimmed milk for 1 h at RT in the absence of light. This was followed by a final washing cycle (3×5 min with 1× PBS, 0.05% [v/v] Tween 20, 3×5 min with 1× PBS, 0.01% [v/v] Triton-X-100 and once for 5 min with 1× PBS). Fluorescence was scanned with an Agilent G2566AA scanner and analysed using the GenePix Pro6 software.

### Functional analysis of PBM data sets

The GenePix data was analyzed using the R statistical software [58] and the Limma Bioconductor package [59]. A Loess normalization was performed on the data to normalize the data within the same slide, assuming that the difference in fluorescence between the DNA and protein channels is independent of the total fluorescence, followed by quantile normalization of the inter-slide variations. Limma was used to assess the genes that showed differential binding when comparing log-ratios (log[protein/DNA]).

Functional classification of AP2 target genes was performed on the average of data from 3 independent PBM experiments using the Ingenuity Pathways Analysis (IPA) software (see: www.ingenuity.com).

78 genes previously experimentally assessed as AP2α target genes were obtained by the addition of Transfac lists of genes bound by TFAP2A and of genes regulated (directly or indirectly) by TFAP2A, of which 45 were also represented on the PBM. Potential AP2 binding sites on the immobilized sequences were predicted using a previously described PWM (http://ccg.vital-it.ch/httfs/software/ap2_pfsearch.html; [32]).

### Quantification of protein-DNA interaction by surface plasmon resonance

Measurements were carried out in a Biacore 3000 (GE Healthcare) machine. Immobilization and binding experiments were performed in HNM buffer (10 mM HEPES, pH 7.4, 150 mM NaCl, 5 mM MgCl_2_) at a flow rate of 5 µl/min unless otherwise stated. Streptavidin was covalently coupled to the surface of a sensor chip CM5 using standard amine coupling procedure by injecting the reagents in the following order: 35 µl 0.05 M NHS/0.2 M EDC mixture, 50 µl streptavidin (300 µg/ml) in 10 mM acetic acid (pH 4.5) and 35 µl 0.1 M ethanolamine. Finally 100 µl SDS 0.5% at 100 µl/min was injected to remove unbound proteins. The amount of immobilized streptavidin was approximately 3000 RU. Biotinylated DNA fragments were diluted in HNM buffer (1–2 ng/µl) and injected over the streptavidin-coated surface until desired amount was immobilized (typically between 300 and 500 RU). Unbound DNA was removed by injecting 100 µl SDS 0.5% at 100 µl/min. Different concentrations of AP2α ranging from 0 to 400 nM were applied in a random order to the surface for 10 minutes in order to reach equilibrium. Surface regeneration was achieved with 100 µl SDS 0.5% at 100 µl/min to dissociate bound proteins. One channel without DNA probes was used as reference in all experiments to correct the binding response for bulk refractive index changes and unspecific binding. Apparent affinity constants were obtained by plotting the responses at equilibrium versus AP2α concentration. BiaEvaluation Software Version 4.1 (Biacore) and IGOR Pro Version 5.05A (Wavemetrics) were used for data processing.

### Reporter plasmids and transfection assays

The expression cassette, consisting of a minimal CMV promoter and eGFP coding sequence as reported previously (pBSGFP_min_, [60]), was flanked by *Bam*HI and *Xba*I restriction sites by PCR and inserted into a pBSKSII backbone resulting in plasmid pJK6-CMV_min_-eGFP. Human genomic DNA sequences identified as potential AP2α binding sites from assays with the hu6k arrays (OCM and GAS2) and one sequence assigned as an AP2α target by weight-matrix analysis (KLK-5) were inserted upstream on the minimal CMV promoter of pJK6-CMV_min_-eGFP, resulting in three reporter plasmids (see Table S2). The DNA sequence feature and biological function of human genes OCM [39], [40], GAS2 [33], [34], [61], [62], [63] and KLK-5 [35], [36], [37], [38], [64], [65], [66], [67], [68], [69], [70] were described elsewhere.

The effects of AP2α expression on the eGFP expression vectors were studied by transient co-transfections of eGFP reporter constructs previously described, and AP2α expressing vector pSP72(RSV)AP2 or the empty pSP72(RSV)NN plasmid as control [27]. Total amounts of DNA per transfection were adjusted with pUC118 plasmid DNA. Transfections were performed using the Fugene reagent (Roche) according to the manufacturer's instructions. Four days after transfection, cells were detached utilizing ESGRO Complete Enzyme-Free Dissociation Solution (Millipore) and eGFP expression levels were recorded on a CyAn ADP analyzer (Dako).

### Chromatin Immunoprecipitation and Quantitative PCR

Chromatin immunoprecipitation (ChIP) and quantitative PCR (qPCR) were essentially performed as previously described [71], [72]. Primary antibodies AP2α(C-18):sc-184 (Santa Cruz Biotechnologies) and anti-Pol II (Abcam, ab5095) were utilized for the immunoprecipitation reactions. qPCRs were performed with a Roche LightCycler 480 machine utilizing the Roche Light Cycler SYBR Green Master Mix (Roche) according to the manufacturer's instructions. Primers were designed using the primer 3 software [73].

## Supporting Information

Figure S1 **Analysis of AP2α binding to PBM DNA sequences.**
(TIF)

Figure S2 **AP2α weight matrix as computed from SELEX data.**
(TIF)

Figure S3 **Correlation of AP-2α binding scores as predicted** ***in silico*** **(A) or determined using a PBM (B).**
(TIF)

Figure S4 **Additional network diagrams illustrating prominent molecular relationships of the products of AP2α-bound genes.**
(TIF)

Figure S5 **Functional validation in HEPG2 cells of AP-2α target genes, as selected from PBM and weight matrix-based predictions.**
(TIF)

Table S1 **Potential AP2α target sequences identified by PBM using the AP2α purified protein.** Sequences bound by recombinant AP2α protein on hu6k microarray (P<0.05).
(PDF)

Table S2 **Network associated functions generated by IPA using sequences ID from table S1.**
(PDF)

Table S3 **Human genomic sequences cloned into the eGFP reporter plasmid for experimental validation.** Highest AP2α weight matrix score are indicated in the table and corresponding sequences are underlined.
(PDF)

Table S4 **Potential AP2α target sequences identified by PBM using the nuclear protein extract from normal breast tissues.** Sequences bound by AP2α protein in nuclear cell extract from healthy breast tissues on hu6k microarray (P<0.05).
(XLS)

Table S5 **Potential AP2α target sequences identified by PBM using the nuclear protein extract from breast tumor tissues.** Sequences bound by AP2α protein in nuclear cell extract from cancer breast tissues on hu6k microarray (P<0.05).
(PDF)

Table S6 **Analysis of the first 50 sequences found to be bound by AP2α in recombinant AP2 extract and in normal and cancer tissues extracts.** Scores were defined by Weight Matrix Prediction Algorithms for AP2α and p53. For AP2α, scores <30 were defined as low binding sites. For p53, scores <50 were defined as low binding sites.
(PDF)

Table S7 **Network associated with disease generated by IPA using the 149 genes differentially regulated in normal versus cancer tissues nuclear extracts.**
(XLS)
