# Supplementary figures and images for: Protein-Binding Microarray Analysis of Tumor Suppressor AP2α Target Gene Specificity
Source: PLoS One. 2011 Aug 18;6(8):e22895. doi: 10.1371/journal.pone.0022895 (PMC3158074; doi:10.1371/journal.pone.0022895)

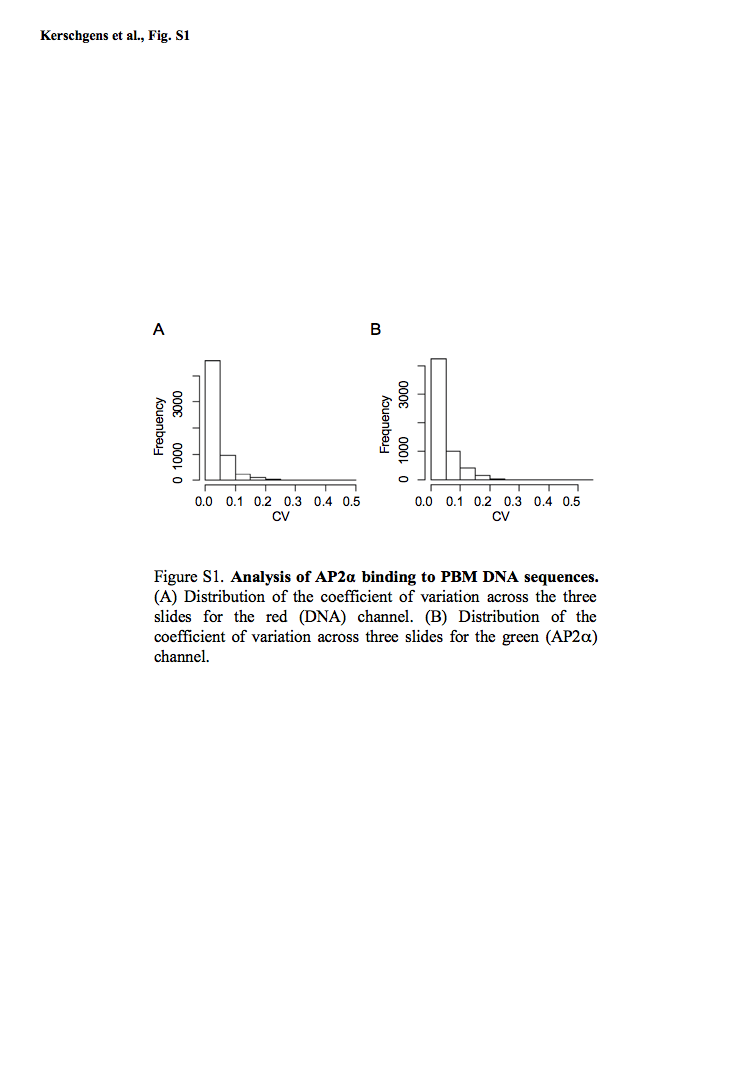

Supplement: Figure S1 — Analysis of AP2α binding to PBM DNA sequences. (TIF) [file pone.0022895.s001.tif]

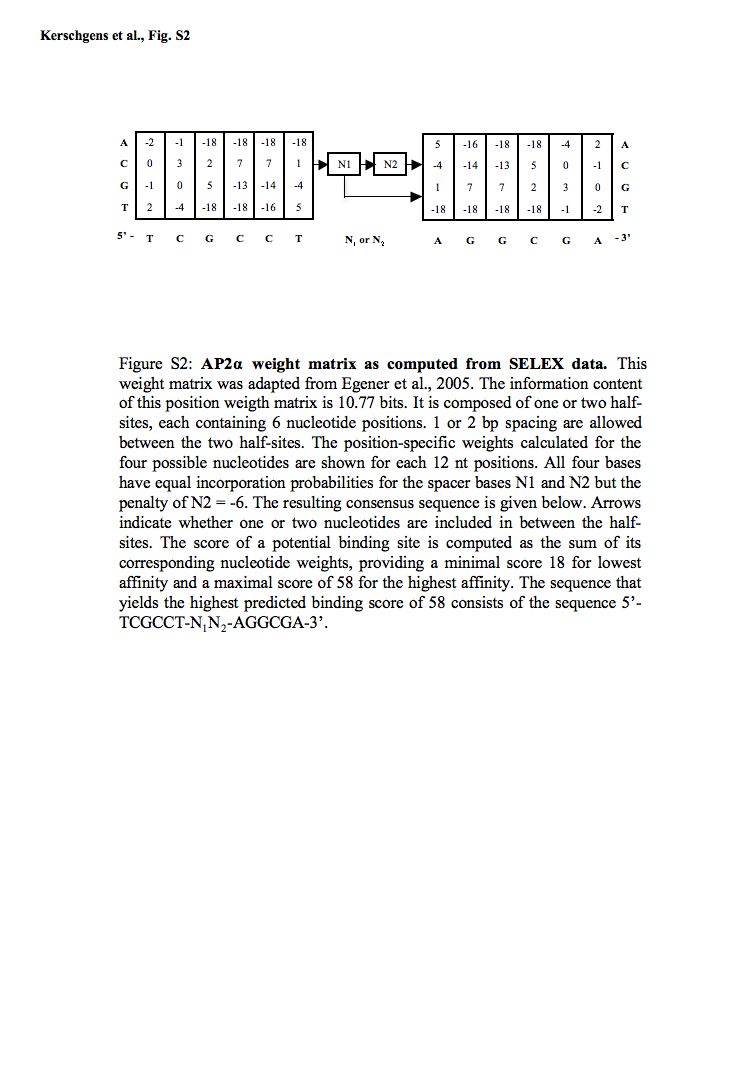

Supplement: Figure S2 — AP2α weight matrix as computed from SELEX data. (TIF) [file pone.0022895.s002.tif]

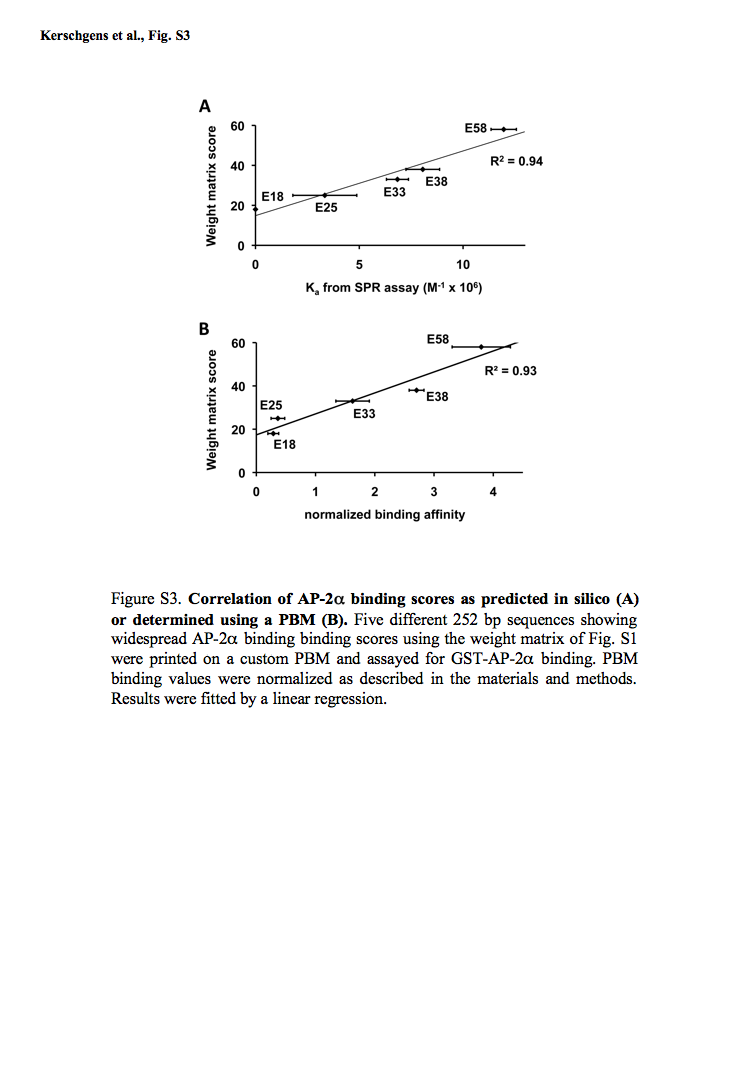

Supplement: Figure S3 — Correlation of AP-2α binding scores as predicted in silico (A) or determined using a PBM (B). (TIF) [file pone.0022895.s003.tif]

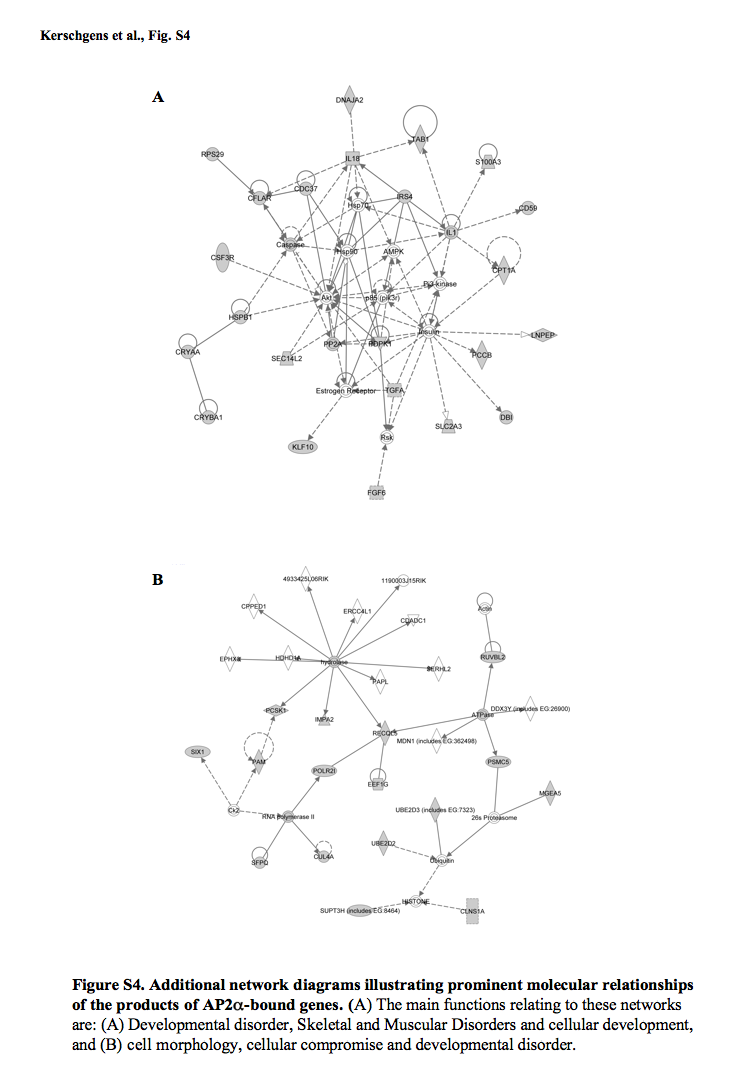

Supplement: Figure S4 — Additional network diagrams illustrating prominent molecular relationships of the products of AP2α-bound genes. (TIF) [file pone.0022895.s004.tif]

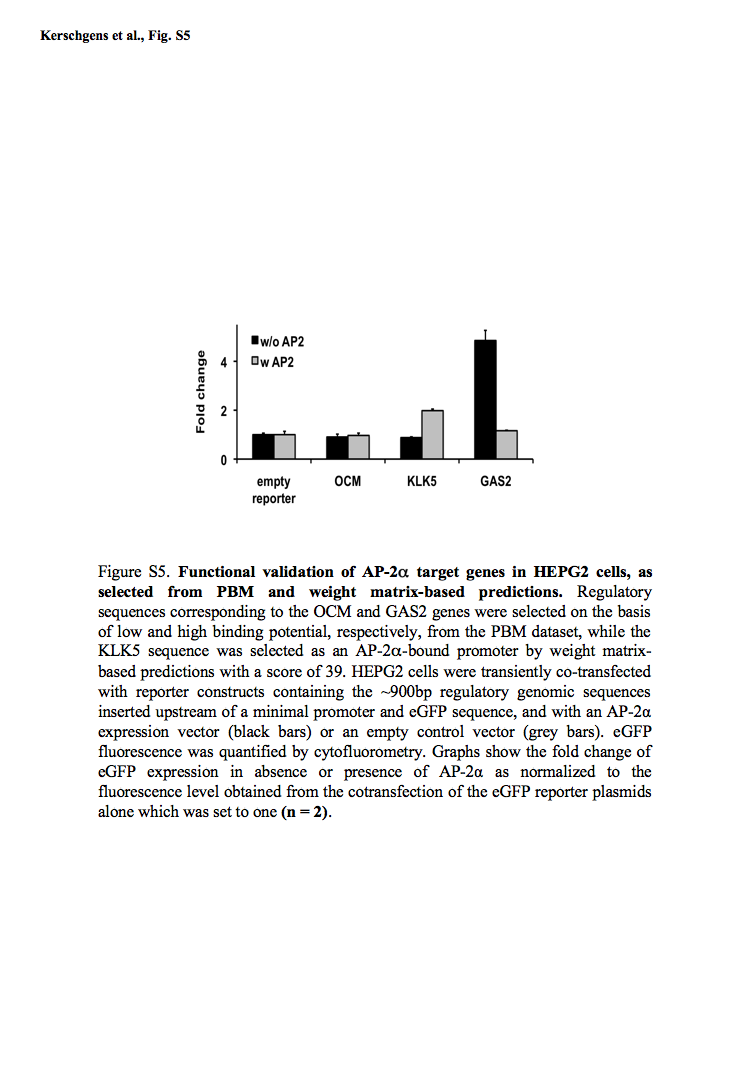

Supplement: Figure S5 — Functional validation in HEPG2 cells of AP-2α target genes, as selected from PBM and weight matrix-based predictions. (TIF) [file pone.0022895.s005.tif]
