# Supplementary material for: Protein-Binding Microarray Analysis of Tumor Suppressor AP2α Target Gene Specificity
Source: PLoS One. 2011 Aug 18;6(8):e22895. doi: 10.1371/journal.pone.0022895 (PMC3158074; doi:10.1371/journal.pone.0022895)
Supplement: Table S1 — Potential AP2α target sequences identified by PBM using the AP2α purified protein. Sequences bound by recombinant AP2α protein on hu6k microarray (P<0.05). (PDF) [file pone.0022895.s006.pdf]

Table S1: Potential AP2α target sequences identified by PBM using the AP2α purified protein

Name	ID	Description	logFC	ıverage AP2 binding	P.Value
PEX11A	NM_003847	peroxisomal biogenesis factor 11A	0.86	2.12	0.000
SIP2-28	NM_006384	calcium and integrin binding 1 (calmyrin)	0.99	1.84	0.001
PDPK1	UP_25		0.82	1.61	0.001
HSPE1	NM_002157	heat shock 10kDa protein 1 (chaperonin 10)	0.70	2.23	0.001
XIP	NM_006402	hepatitis B virus x interacting protein	0.67	2.22	0.002
FUT2	IN_30		0.70	1.96	0.002
KLK8	NM_007196	kallikrein 8 (neuropsin/ovasin)	0.59	1.96	0.002
PSMC5	NM_002805	proteasome (prosome, macropain) 26S subunit, ATPase, 5	0.72	2.10	0.002
FKBP6	NM_178125	tripartite motif-containing 50A	0.64	1.92	0.002
CRYGA	NM_014617	crystallin, gamma A	0.63	2.44	0.003
SNK	NM_006622	polo-like kinase 2 (Drosophila)	0.88	1.65	0.003
TIEG	NM_005655	TGFB inducible early growth response	0.77	2.02	0.003
CHRNE	NM_000080	cholinergic receptor, nicotinic, epsilon polypeptide	0.64	2.37	0.003
Kifap3	NM_014970	kinesin-associated protein 3	0.82	2.16	0.003
SLC25A3	NM_002635	solute carrier family 25 (mitochondrial carrier; phosphate carrier), member 3	0.52	1.81	0.003
NUDT6	NM_198041	nudix (nucleoside diphosphate linked moiety X)-type motif 6	0.84	0.93	0.003
FACTP140	NM_007192	suppressor of Ty 16 homolog (S. cerevisiae)	0.54	2.35	0.004
TRO	NM_016157	trophinin	0.49	2.28	0.004
CLNS1A	NM_001293	chloride channel, nucleotide-sensitive, 1A	0.49	2.11	0.004
TIM17B	NM_005834	translocase of inner mitochondrial membrane 17 homolog B (yeast)	0.60	2.06	0.004
RAD51L3	NM_002878	RAD51-like 3 (S. cerevisiae)	0.55	2.30	0.004
STK19	NM_004197	serine/threonine kinase 19	0.96	2.16	0.004
SMARCB1	NM_003073	SWI/SNF related, matrix associated, actin dependent regulator of chromatin, subfamily b, member 1	0.58	2.30	0.004
PRG4	NM_005807	proteoglycan 4, (megakaryocyte stimulating factor, articular superficial zone protein, camptodactyly, arthropathy, coxa vara, pericarditis syndrome)	0.82	2.04	0.005
STC1	NM_003155	stanniocalcin 1	0.86	2.26	0.005
ATRN	NM_139321	attractin	0.88	2.27	0.005
NUP50	NM_007172	nucleoporin 50kDa	0.44	2.24	0.005
UBE2D2	NM_003339	ubiquitin-conjugating enzyme E2D 2 (UBC4/5 homolog, yeast)	0.48	2.20	0.006
RRM1	NM_001033	ribonucleotide reductase M1 polypeptide	0.56	2.23	0.006
HML2	NM_182906	C-type (calcium dependent, carbohydrate-recognition domain) lectin, superfamily member 14 (macrophage-derived)	0.42	2.16	0.006
G5B	NM_021221	lymphocyte antigen 6 complex, locus G5B	0.93	1.70	0.006
CRHR2	NM_001883	corticotropin releasing hormone receptor 2	0.47	2.56	0.006
IDH3A	NM_005530	isocitrate dehydrogenase 3 (NAD+) alpha	1.35	1.26	0.007
DBI	NM_020548	diazepam binding inhibitor (GABA receptor modulator, acyl-Coenzyme A binding protein)	0.54	2.32	0.007
CALCR	NM_001742	calcitonin receptor	0.64	2.20	0.007
H4FH	NM_003543	histone 1, H4h	0.53	2.09	0.007
POP4	NM_006627	processing of precursor 4, ribonuclease P/MRP subunit (S. cerevisiae)	0.68	1.66	0.007
RPS29	NM_001032	ribosomal protein S29	0.91	1.59	0.007
IFI30	NM_006332	interferon, gamma-inducible protein 30	0.68	1.15	0.007
IDH3B	NM_174855	isocitrate dehydrogenase 3 (NAD+) beta	1.26	1.26	0.008
HTATIP	NM_006388	HIV-1 Tat interacting protein, 60kDa	0.94	1.98	0.008
TYMSTR	NM_006564	chemokine (C-X-C motif) receptor 6	0.48	2.21	0.008
NDUFB3	NM_002491	NADH dehydrogenase (ubiquinone) 1 beta subcomplex, 3, 12kDa	0.62	2.17	0.008
CDC37	NM_007065	CDC37 cell division cycle 37 homolog (S. cerevisiae)	1.06	0.40	0.008
H3FJ	NM_003535	histone 1, H3j	0.38	2.08	0.008
COL5A3	NM_015719	collagen, type V, alpha 3	0.52	2.30	0.008

CLK2	IN_154		0.53	2.50	0.009
RABGGTA	NM_004581	Rab geranylgeranyltransferase, alpha subunit	0.47	2.24	0.009
DNASE1	NM_005223	deoxyribonuclease I	0.61	1.85	0.009
IL1RAPL1	IN_113		0.82	1.67	0.009
OPN1SW	NM_001708	opsin 1 (cone pigments), short-wave-sensitive (color blindness, tritan)	0.54	1.97	0.010
PDCD10	NM_145859	programmed cell death 10	0.69	2.29	0.010
FGF6	NM_020996	fibroblast growth factor 6	0.54	2.30	0.010
RPS25	NM_001028	ribosomal protein S25	0.76	2.30	0.010
SLC22A4	NM_003059	solute carrier family 22 (organic cation transporter), member 4	0.53	2.18	0.010
SAS10	NM_020368	disrupter of silencing 10	0.41	2.51	0.010
HOXD3	IN_175		0.40	2.33	0.010
CDH16	NM_004062	cadherin 16, KSP-cadherin	1.13	1.52	0.010
CKS1	UP_159		0.53	1.94	0.010
VTI2	NM_006370	vesicle transport through interaction with t-SNAREs homolog 1B (yeast)	1.12	2.13	0.010
HREV107	NM_007069	HRAS-like suppressor 3	0.44	1.67	0.011
HUG1	NM_020527		0.63	1.95	0.011
CDKN1B	NM_004064	cyclin-dependent kinase inhibitor 1B (p27, Kip1)	0.52	2.14	0.011
IRAK-M	NM_007199	interleukin-1 receptor-associated kinase 3	0.99	1.66	0.011
PHEX	NM_000444	phosphate regulating endopeptidase homolog, X-linked (hypophosphatemia, vitamin D resistant rickets)	0.53	2.12	0.011
M83	NM_021259	transmembrane protein 8 (five membrane-spanning domains)	0.53	2.26	0.012
ORC6L	NM_014321	origin recognition complex, subunit 6 homolog-like (yeast)	0.49	1.93	0.012
P5-1	NM_006674	HLA complex P5	0.87	1.89	0.012
ACT	NM_020482	four and a half LIM domains 5	0.50	2.25	0.012
H4FG	NM_003542	histone 1, H4c	0.49	2.26	0.013
IF	NM_000204	I factor (complement)	1.28	1.51	0.013
ARPP-19	NM_006628	cyclic AMP phosphoprotein, 19 kD	0.94	2.30	0.013
EMILIN	NM_007046	elastin microfibril interfacer 1	0.68	1.76	0.013
CLCN5	NM_000084	chloride channel 5 (nephrolithiasis 2, X-linked, Dent disease)	0.61	2.47	0.013
PMVK	NM_006556	phosphomevalonate kinase	0.74	2.23	0.013
HSU93850	IN_101		0.33	2.41	0.014
CTRC	NM_007272	chymotrypsin C (caldecrin)	0.68	2.26	0.014
DVL2	NM_004422	dishevelled, dsh homolog 2 (Drosophila)	0.32	2.39	0.014
CAMP-GEFII	NM_007023	Rap guanine nucleotide exchange factor (GEF) 4	0.40	2.15	0.014
RNF3	IN_83		0.35	1.71	0.015
H4FI	NM_003544	histone 1, H4b	0.84	1.93	0.015
SDS	NM_006843	serine dehydratase	0.45	2.25	0.015
UBE2D3	NM_181889	ubiquitin-conjugating enzyme E2D 3 (UBC4/5 homolog, yeast)	0.57	1.51	0.015
IMPA2	IN_133		0.84	2.08	0.019
SDCCAG10	NM_005869	serologically defined colon cancer antigen 10	0.48	1.98	0.016
RAP2B	NM_002886	RAP2B, member of RAS oncogene family	0.40	2.42	0.016
TERA	NM_021238	chromosome 12 open reading frame 14	0.46	2.49	0.016
EEF1G	IN_171		0.55	2.42	0.016
BRCA2	NM_000059	breast cancer 2, early onset	0.83	1.48	0.016
FACL4	NM_004458	acyl-CoA synthetase long-chain family member 4	0.39	2.48	0.016
HAX1	NM_006118	HS1 binding protein	0.42	1.79	0.016
ARMET	NM_006010	arginine-rich, mutated in early stage tumors	0.70	2.27	0.017
AKAP10	NM_007202	A kinase (PRKA) anchor protein 10	0.52	1.95	0.017
TIAM1	NM_003253	T-cell lymphoma invasion and metastasis 1	0.70	2.44	0.017
EPHB4	NM_004444	EphB4	0.53	2.05	0.017
BRD3	IN_118		0.50	2.17	0.017

RARRES3	NM_004585	retinoic acid receptor responder (tazarotene induced) 3	0.61	2.22	0.017
TRN2	IN_106		0.41	1.02	0.017
SH3BP1	NM_018957	SH3-domain binding protein 1	0.49	2.26	0.017
ABCA8	IN_76		1.13	1.98	0.017
MTX2	NM_006554	metaxin 2	0.84	1.74	0.017
OAZ3	NM_016178	ornithine decarboxylase antizyme 3	0.31	2.36	0.017
TGIF	NM_173209	TGFB-induced factor (TALE family homeobox)	0.32	2.04	0.018
SNAPC2	NM_003083	small nuclear RNA activating complex, polypeptide 2, 45kDa	0.49	2.02	0.018
ELK4	IN_170		0.46	2.45	0.018
JDP1	NM_021800	DnaJ (Hsp40) homolog, subfamily C, member 12	0.31	2.35	0.018
WDR11	NM_018117	WD repeat domain 11	0.47	2.55	0.018
SOX21	NM_007084	SRY (sex determining region Y)-box 21	0.48	2.09	0.018
SCYA19	NM_006274	chemokine (C-C motif) ligand 19	0.41	2.42	0.018
RUVBL2	NM_006666	RuvB-like 2 (E. coli)	0.49	1.67	0.018
TIM23	NM_006327	translocase of inner mitochondrial membrane 23 homolog (yeast)	0.37	2.43	0.019
SDCCAG16	NM_006649	UTP14, U3 small nucleolar ribonucleoprotein, homolog A (yeast)	0.39	2.38	0.019
CBX5	NM_012117	chromobox homolog 5 (HP1 alpha homolog, Drosophila)	0.58	2.41	0.019
PCYT1A	NM_005017	phosphate cytidylyltransferase 1, choline, alpha isoform	0.57	2.04	0.019
CACNG3	NM_006539	calcium channel, voltage-dependent, gamma subunit 3	0.39	2.49	0.019
HNRPF	IN_141		0.58	2.42	0.019
APG5L	NM_004849	APG5 autophagy 5-like (S. cerevisiae)	0.38	2.10	0.019
SOD3	NM_003102	superoxide dismutase 3, extracellular	0.61	2.12	0.019
RARB	NM_000965	retinoic acid receptor, beta	0.32	2.20	0.020
IFNA14	NM_002172	interferon, alpha 14	0.45	2.31	0.020
CSF3R	NM_000760	colony stimulating factor 3 receptor (granulocyte)	0.57	2.52	0.020
RAP1GA1	NM_002885	RAP1, GTPase activating protein 1	0.47	2.53	0.020
CHRM5	IN_122		0.48	2.42	0.020
ADMR	NM_007264	adrenomedullin receptor	0.49	2.08	0.020
LOC64145	NM_022340	zinc finger, FYVE domain containing 20	0.74	2.16	0.021
SWAP2	NM_007056	splicing factor, arginine/serine-rich 16 (suppressor-of-white-apricot homolog, Drosophila)	0.47	1.69	0.021
P12	NM_019896	polymerase (DNA-directed), epsilon 4 (p12 subunit)	0.65	2.25	0.021
DSCR4	IN_95		0.41	2.08	0.021
KLRA1	NM_006611	killer cell lectin-like receptor subfamily A, member 1	0.88	1.54	0.021
CASP4	NM_001225	caspase 4, apoptosis-related cysteine protease	0.28	2.05	0.022
APEG1	IN_100		0.41	2.53	0.022
PCSK1	NM_000439	proprotein convertase subtilisin/kexin type 1	0.58	1.46	0.022
CRYGS	NM_017541	crystallin, gamma S	0.40	2.39	0.022
FANCG	NM_004629	Fanconi anemia, complementation group G	0.35	2.40	0.022
SF3A2	IN_175		0.34	2.45	0.023
MYBBP1A	NM_014520	MYB binding protein (P160) 1a	1.13	1.72	0.023
CRYBA2	NM_057093	crystallin, beta A2	0.36	2.51	0.023
CDK2	NM_001798	cyclin-dependent kinase 2	0.63	2.35	0.023
IFNA21	NM_002175	interferon, alpha 21	0.80	1.42	0.023
POLQ	IN_92		0.64	1.74	0.023
KCNC4	NM_004978	potassium voltage-gated channel, Shaw-related subfamily, member 4	0.49	2.23	0.024
SPK	NM_004819	symplekin	0.34	2.57	0.024
NCYM	NM_006316		0.95	1.50	0.024
TACC3	IN_116		0.69	1.98	0.025
SUPT3H	NM_003599	suppressor of Ty 3 homolog (S. cerevisiae)	0.80	1.53	0.025
SLC2A3	IN_131		0.30	2.37	0.025

CHC1	IN_164		0.34	2.58	0.025
OXR1	IN_133		0.58	2.08	0.025
FLJ23311	IN_122		0.43	2.36	0.025
AP4B1	NM_006594	adaptor-related protein complex 4, beta 1 subunit	0.77	2.28	0.025
ATP6N1B	NM_020632	ATPase, H+ transporting, lysosomal V0 subunit a isoform 4	0.63	2.14	0.025
CRYBA1	NM_005208	crystallin, beta A1	0.67	2.22	0.026
RANBP7	UP_78		0.54	1.89	0.026
EFS2	NM_005864	embryonal Fyn-associated substrate	0.48	2.34	0.026
SPF30	NM_005871	survival motor neuron domain containing 1	0.92	2.03	0.026
JAZ	NM_012279	zinc finger protein 346	0.52	2.35	0.026
ELL2	UP_112		0.58	2.52	0.026
ZNF140	NM_003440	zinc finger protein 140 (clone pHZ-39)	0.44	2.39	0.027
TCF12	NM_003205	transcription factor 12 (HTF4, helix-loop-helix transcription factors 4)	0.32	2.42	0.027
LANCL1	NM_006055	LanC lantibiotic synthetase component C-like 1 (bacterial)	0.71	1.59	0.027
CLCN3	NM_001829	chloride channel 3	0.58	2.37	0.027
CHL1	NM_006614	cell adhesion molecule with homology to L1CAM (close homolog of L1)	0.38	2.12	0.027
GCHFR	NM_005258	GTP cyclohydrolase I feedback regulator	0.44	2.40	0.027
S100A3	NM_002960	S100 calcium binding protein A3	0.29	2.30	0.027
CUL4A	NM_003589	cullin 4A	0.34	2.24	0.027
NYX	NM_022567	nyctalopin	0.34	2.31	0.027
ICB-1	NM_004848	chromosome 1 open reading frame 38	0.26	2.39	0.028
CTRL	NM_001907	chymotrypsin-like	0.48	2.56	0.028
E2F3:500	NM_001949	E2F transcription factor 3	0.88	1.66	0.028
IMP-2	IN_56		0.43	2.04	0.028
MASA	NM_021204	E-1 enzyme	0.45	2.29	0.028
CRTL1	NM_001884	hyaluronan and proteoglycan link protein 1	0.52	2.33	0.029
PAK4	NM_005884	p21(CDKN1A)-activated kinase 4	0.31	2.04	0.029
YME1L1	NM_014263	YME1-like 1 (S. cerevisiae)	0.43	2.14	0.029
COPA	NM_004371	coatomer protein complex, subunit alpha	0.38	2.33	0.030
CFLAR	IN_161		0.44	2.49	0.030
GAS2	IN_152		0.93	1.96	0.030
CDIPT	NM_006319	CDP-diacylglycerol--inositol 3-phosphatidyltransferase (phosphatidylinositol synthase)	0.28	2.21	0.030
FMO2	NM_001460	flavin containing monooxygenase 2	0.29	1.84	0.030
E2F4	NM_001950	E2F transcription factor 4, p107/p130-binding	0.58	2.13	0.031
SORD	NM_003104	sorbitol dehydrogenase	1.00	2.28	0.031
CLCNKA	NM_004070	chloride channel Ka	0.53	2.40	0.031
DUSP10	IN_89		1.06	1.43	0.031
CFDP1	NTF_106		0.44	2.44	0.031
COX15	NM_078470	COX15 homolog, cytochrome c oxidase assembly protein (yeast)	0.73	1.61	0.032
RNASE4	IN_35		0.38	2.20	0.032
DNAJA2	NM_005880	DnaJ (Hsp40) homolog, subfamily A, member 2	0.30	1.94	0.032
SLC17A3	NM_006632	solute carrier family 17 (sodium phosphate), member 3	0.28	2.08	0.032
LNPEP	NM_005575	leucyl/cystinyl aminopeptidase	0.39	2.43	0.032
CGA	NM_000735	glycoprotein hormones, alpha polypeptide	0.43	2.52	0.033
CHD1L	NM_024568		0.83	1.88	0.033
TAB1	NM_006116	mitogen-activated protein kinase kinase kinase 7 interacting protein 1	0.68	2.16	0.033
LOC58511	NM_021233	DNase II-like acid DNase	0.35	2.17	0.033
EBP	NM_006579	emopamil binding protein (sterol isomerase)	0.61	2.30	0.033
CENPA	NM_001809	centromere protein A, 17kDa	0.41	2.53	0.033
SFPQ	NM_005066	splicing factor proline/glutamine rich (polypyrimidine tract binding protein associated)	0.27	2.18	0.034

SCAM-1	IN_142		0.24	2.68	0.034
CENPE	NM_001813	centromere protein E, 312kDa	0.28	2.41	0.034
DNAJC3	NM_006260	DnaJ (Hsp40) homolog, subfamily C, member 3	0.49	2.22	0.034
SF3B4	NM_005850	splicing factor 3b, subunit 4, 49kDa	0.40	2.05	0.034
LILRA2	NM_006866	leukocyte immunoglobulin-like receptor, subfamily A (with TM domain), member 2	1.02	1.58	0.034
ZFP95	NM_145102	zinc finger protein 95 homolog (mouse)	0.69	2.12	0.034
PNKP	NM_007254	polynucleotide kinase 3'-phosphatase	0.48	2.16	0.034
PET112L	NM_004564	PET112-like (yeast)	0.56	1.85	0.034
IL2	NM_000586	interleukin 2	0.69	2.42	0.034
RASSF1	NM_170712	Ras association (RalGDS/AF-6) domain family 1	0.68	1.78	0.035
CLDN16	NM_006580	claudin 16	0.49	2.30	0.035
MOV34-34KD	NM_006833	COP9 constitutive photomorphogenic homolog subunit 6 (Arabidopsis)	0.52	2.44	0.035
PABPN1	NM_004643	poly(A) binding protein, nuclear 1	0.83	2.11	0.035
RAD9	NM_004584	RAD9 homolog A (S. pombe)	0.51	2.41	0.035
BMP6	NM_001718	bone morphogenetic protein 6	1.21	0.95	0.035
CLIC3	NM_004669	chloride intracellular channel 3	0.34	2.49	0.036
TOPBP1	NM_007027	topoisomerase (DNA) II binding protein 1	0.46	2.14	0.036
IGFBP5	NM_000599	insulin-like growth factor binding protein 5	0.51	2.27	0.036
MAN2A1	NM_002372	mannosidase, alpha, class 2A, member 1	0.44	2.17	0.036
PAM	IN_178		0.29	2.32	0.036
CREBL2	NM_001310	cAMP responsive element binding protein-like 2	0.51	2.27	0.036
ARGBP2	NTF_106		1.00	1.18	0.036
IMAGE145052	UP_69		0.40	2.00	0.037
POLD2	NM_006230	polymerase (DNA directed), delta 2, regulatory subunit 50kDa	0.50	2.26	0.037
EIF5	NM_001969	eukaryotic translation initiation factor 5	0.45	2.49	0.037
PCCB	NM_000532	propionyl Coenzyme A carboxylase, beta polypeptide	0.81	2.38	0.037
POMT1	NM_007171	protein-O-mannosyltransferase 1	0.55	2.29	0.037
CC1.3	NM_004902	RNA-binding region (RNP1, RRM) containing 2	0.28	2.37	0.038
PPP2R3	NM_181897	protein phosphatase 2 (formerly 2A), regulatory subunit B'', alpha	0.29	2.47	0.038
CSTF3	NM_001326	cleavage stimulation factor, 3' pre-RNA, subunit 3, 77kDa	0.47	2.49	0.038
P23	NM_006601	unactive progesterone receptor, 23 kD	0.40	1.95	0.038
CPT1A	IN_169		0.78	2.20	0.038
CLPS	NM_001832	colipase, pancreatic	0.50	2.50	0.038
CD59	NM_000611	CD59 antigen p18-20 (antigen identified by monoclonal antibodies 16.3A5, EJ16, EJ30, EL32 and G344)	0.33	2.27	0.038
F11	NM_000128	coagulation factor XI (plasma thromboplastin antecedent)	0.27	2.34	0.039
SLC4A8	NM_004858	solute carrier family 4, sodium bicarbonate cotransporter, member 8	0.33	2.55	0.039
CHST2	NM_004267	carbohydrate (N-acetylglucosamine-6-O) sulfotransferase 2	0.41	2.47	0.039
ZWINT	NM_007057	ZW10 interactor	0.37	2.37	0.039
BAIAP3	NM_003933	BAI1-associated protein 3	0.27	2.45	0.040
RECQL5	NM_004259	RecQ protein-like 5	0.32	2.44	0.040
POLR2I	NM_006233	polymerase (RNA) II (DNA directed) polypeptide I, 14.5kDa	0.25	2.19	0.040
FLJ23476	NM_024640	ischemia/reperfusion inducible protein	0.31	2.41	0.040
H6PD	NM_004285	hexose-6-phosphate dehydrogenase (glucose 1-dehydrogenase)	0.40	2.43	0.040
ITGA6	IN_139		0.24	2.39	0.040
TGFA	NM_003236	transforming growth factor, alpha	0.32	2.58	0.041
HRH3	NM_007232	histamine receptor H3	0.30	2.52	0.041
TRA1	NM_003299	tumor rejection antigen (gp96) 1	0.31	2.38	0.041
RER1	IN_74		1.00	0.98	0.041
BLTR2	NM_019839	leukotriene B4 receptor 2	0.46	2.25	0.041
RB1:500	NM_000321	retinoblastoma 1 (including osteosarcoma)	0.28	2.58	0.041

MGEA5	NM_012215	meningioma expressed antigen 5 (hyaluronidase)	0.34	2.37	0.041
DO	NM_021071	Dombrock blood group	0.64	2.37	0.041
POP3	IN_128		0.75	2.26	0.042
TR	NM_006440	thioredoxin reductase 2	0.33	2.37	0.042
IL18	NM_001562	interleukin 18 (interferon-gamma-inducing factor)	0.51	2.22	0.043
DPEP1	NM_004413	dipeptidase 1 (renal)	1.86	1.03	0.043
POU2F1	NM_002697	POU domain, class 2, transcription factor 1	0.34	2.51	0.043
NASP	UP_169		0.53	2.60	0.043
RCN2	NM_002902	reticulocalbin 2, EF-hand calcium binding domain	0.30	2.41	0.044
GPR10	NM_004248	G protein-coupled receptor 10	0.40	2.37	0.044
ARSE	NM_000047	arylsulfatase E (chondrodysplasia punctata 1)	1.01	2.32	0.044
HSPB1	NM_001540	heat shock 27kDa protein 1	0.75	2.42	0.044
DR6	NM_014452	tumor necrosis factor receptor superfamily, member 21	0.54	2.54	0.044
SPR	NM_003124	sepiapterin reductase (7,8-dihydrobiopterin:NADP+ oxidoreductase)	0.44	2.37	0.044
SHC1	NM_183001	SHC (Src homology 2 domain containing) transforming protein 1	0.35	2.02	0.045
TNNI3	NM_000363	troponin I, cardiac	0.36	2.53	0.045
LOC59346	NM_176871	PDZ and LIM domain 2 (mystique)	0.50	2.33	0.045
DRD5	NM_000798	dopamine receptor D5	0.23	2.54	0.045
NUDT1	IN_142		0.37	2.49	0.046
DOC-1R	NM_005851	tumor suppressor deleted in oral cancer-related 1	0.48	1.78	0.046
CSTF2	NM_001325	cleavage stimulation factor, 3' pre-RNA, subunit 2, 64kDa	0.36	2.39	0.046
CRYAA	NM_000394	crystallin, alpha A	0.42	2.54	0.047
KIAA0118	NM_014999	RAB21, member RAS oncogene family	0.46	2.50	0.047
IRS4	NM_003604	insulin receptor substrate 4	0.84	1.14	0.047
SIX1	NM_005982	sine oculis homeobox homolog 1 (Drosophila)	0.28	2.43	0.048
TNFRSF12	NM_003790	tumor necrosis factor receptor superfamily, member 25	0.59	2.40	0.048
AGTRL2	UP_1		0.42	2.09	0.048
CPLX1	NM_006651	complexin 1	0.61	2.32	0.049
VAMP4	NM_003762	vesicle-associated membrane protein 4	0.35	2.33	0.049
HHLA2	IN_116		1.18	1.18	0.049
APC10	NM_014885	anaphase promoting complex subunit 10	0.44	2.35	0.049
SEC14L2	NM_012429	SEC14-like 2 (S. cerevisiae)	0.44	2.14	0.049
WASF1	IN_88		0.28	2.15	0.049
ETR101	NM_004907	immediate early response 2	0.38	2.49	0.049
UNRIP	NM_007178	serine/threonine kinase receptor associated protein	0.40	2.57	0.049
ZNF274	NM_016324	zinc finger protein 274	0.42	2.39	0.049
EN1	NM_001426	engrailed homolog 1	1.00	2.22	0.050
