# Supplementary material for: Protein-Binding Microarray Analysis of Tumor Suppressor AP2α Target Gene Specificity
Source: PLoS One. 2011 Aug 18;6(8):e22895. doi: 10.1371/journal.pone.0022895 (PMC3158074; doi:10.1371/journal.pone.0022895)
Supplement: Table S2 — Network associated functions generated by IPA using sequences ID from table S1. (PDF) [file pone.0022895.s007.pdf]

Table S2: Networks of recombinant AP2α-bound genes identified with the Ingenuity Pathway Analysis

Network	Molecules in Network	Score	Focus Molecules	Top Functions
1	Akt, AMPK, Caspase, **CD59, CDC37, CFLAR, CPT1A, CRYAA, CRYBA1, CSF3R, DBI, DNAJA2**, Estrogen Receptor, **FGF6**, Hsp70, Hsp90, **HSPB1**, IL1, **IL18**, Insulin, **IRS4, KLF10, LNPEP**, p85 (pik3r), **PCCB, PDPK1**, Pi3-kinase, PP2A, **RPS29,** Rsk, **S100A3, SEC14L2, SLC2A3, TAB1, TGFA**	39	23	Developmental Disorder, Skeletal and Muscular Disorders, Cellular Development
2	**ATG5 (includes EG:9474), CCL19, CENPA, CENPE, CLK2**, Cyclin E, **E2F4, ELL2, HSPE1, IFI30**, Ifn, IFN Beta, **IFNA14, IFNA21**, IgG, IL12 (complex), Immunoglobulin, Interferon alpha, IRG, **ITGA6, KLK8, KLRA1 (includes EG:10748)**, Laminin1, **LILRA2**, MHC CLASS I (family), MHC Class II (complex), NFkB (complex), **RARRES3, RRM1 (includes EG:6240), SMARCB1**, STAT5a/b, Tlr, **TNFRSF25, TOPBP1, ZNF274**	35	21	Cellular Development, Cell-To-Cell Signaling and Interaction, Embryonic Development
3	Alp, Alpha tubulin, Ap1, **BRCA2, CACNG3, CASP4, CDK2, CGA, COL5A3** Collagen type I, Creb, **CREBL2, CTRC, CTRL, EBP, F11, FANCG,** FSH, hCG, **IL2**, LDL, Lh, Mapk, peptidase, Pka, **POU2F1, RCC1 (includes EG:1104), SNAPC2, SOD3, STC1**, Tgf beta, **TRO, VAMP4,** Vegf, **YME1L1**	34	21	Cell Death, Cancer, Genetic Disorder
4	Calpain, **CBX5**, Cdc2, **CDH16, CDKN1B, CSTF2 (includes EG:1478)**, Cyclin A, Cyclin B, E2f, ERK1/2, GAS2, HIST1H4C, Histone H1, Histone h3, HSP90B1, IGFBP5, Importin alpha, IPO7, **KAT5**, MAP2K1/2, **NASP, NUP50, OPN1SW, PCYT1A, PNKP, PPP2R3A, RAP1GAP (includes EG:5909)**, Rar, **RARB, RASSF1**, Rb, Rxr, Smad2/3-Smad4, **TGIF1**, VitaminD3-VDR-RXR	32	20	Cancer, Cell Cycle, Embryonic Development
5	26s Proteasome, 1190003J15RIK, 4933425L06RIK, Actin, ATPase, CDADC1, Ck2, **CLNS1A,** CPPED1, **CUL4A**, DDX3Y (includes EG:26900), **EEF1G**, EPHX3, ERCC4L1, HDHD1A, HISTONE, hydrolase, **IMPA2,** MDN1 (includes EG:362498), **MGEA5, PAM**, PAPL, **PCSK1, POLR2I, PSMC5, RECQL5** RNA polymerase II, **RUVBL2**, SERHL2, **SFPQ, SIX1, SUPT3H (includes EG:8464), UBE2D2, UBE2D3 (includes EG:7323)**, Ubiquitin	24	16	Cell Morphology, Cellular Compromise, Developmental Disorder
6	14-3-3, **BMP6, CALCR, CLCN3, DNASE1, DUSP10, DVL2 (includes EG:1856), ELK4**, ERK, G-Actin, **HAX1**, Integrin, Jnk, **KCNC4, KIFAP3, L1CAM**, Mek, Mmp, Nfat (family), P38 MAPK, **PAK4**, Pdgf, PDGF BB, PI3K, Pkc(s), Rac, Rap1, Ras, Ras homolog, **SH3BP1, SHC1, SORD**, TCR, **TIAM1, WASF1**	23	17	Cellular Movement, Cardiovascular System Development and Function, Cell Morphology
7	**AP4B1**, C12ORF11, **CRHR2,** CSRP1, **CSTF3,** CYB561, GLRX2, **GPR182, H6PD,** Histone h4, **HOXD3,** LRBA, **NDUFB3,** NDUFC1, OAZ, **OAZ3 (includes EG:51686)**, ODC1, ODC1-OAZ, PAPD7, PCNA, PDZK1IP1, **POLD2,** POLD4, POLI, POLK, POLM, **POLQ,** PTEN, **RAD9A, SPEG, TCF12,** TGFB1, TNFAIP1, **UTP14A, ZWINT (includes EG:11130)**	22	15	Cell Morphology, DNA Replication, Recombination, and Repair, Small Molecule Biochemistry
8	ADCYAP1, **ATRN**, CDO1, CFTR, **CHRNE, CHST2**, CLDN8, **CLDN16, COPA, CRYBA2**, CYB5R2, DGCR6, dihydrotestosterone, **HIST1H3J, IDH3A, IDH3B**, IDH3G, IKBKG, **IL1RAPL1,** isocitrate dehydrogenase (NAD), LOC729505, NAGLU, **PEX11A, RCN2, RNASE4**, SEPW1, SLC1A4, SLC4A7, SVIL, TGM4, TJP1, TJP3, TNF, **TNNI3**, TRIP13	20	14	Carbohydrate Metabolism, Small Molecule Biochemistry, Cell-To-Cell Signaling and Interaction
9	BAI1, **BAIAP3 (includes EG:8938)**, CARM1, **CDIPT, CHD1L, CLCNKA**, DAG1, DNAJB4, **EIF5,** GRIK3, GRIP1, GRIP2, **HHLA2, HIST1H4B**, HNF4A, KIF1B, MIR133A-1, MIR186 (includes EG:406962), MIR291A (includes EG:100049715), MIRN326, NR1I3, NR2F1, **NUDT6, PABPN1**, PAPOLA, **PET112L,** PICK1, **POMT1,** POMT2, PPARGC1B, **PRLHR,** SNW1, SRR, XRCC4, **ZKSCAN5**	18	13	Genetic Disorder, Neurological Disease, Gene Expression
10	**ABCA8,** beta-estradiol, **CFI,** CHCHD8, **COX15,** COX7A2L, **CWC27,** DLEU2, **DNAJC3, DRD5,** E2F1, FKBP9, **FMO2,** GM7625, GM10117, GM10155, **HIST1H4H (includes EG:8365), HNRNPF, HRH3**, HTR5A, L-glutamic acid, LOC646483, MARCKS (includes EG:25603), **MYBBP1A,** MYC, **NUDT1,** ONECUT1, PHB (includes EG:25344), RPS15A (includes EG:267019), SCPEP1, SLC2A4, SPRR3 (includes EG:6707), TMEM126A, TMEM126B, TXNL4A (includes EG:10907)	16	12	Cell Death, Cell Cycle, Connective Tissue Development and Function
11	**ACSL4,** ATP5H (includes EG:10476), **ATP6V0A1,** ATP6V0A4, ATP6V0D1, ATP6V1B2, ATP6V1C1, ATP6V1G1, ATP6V1G3, CBY1, CD40LG, **CHRM5,** CTNNB1, **DPEP1,** EGF, **EPHB4,** FAM189B, FAS, **GCHFR,** GLG1, GPER, **HAPLN1,** HIF1A, **MANF,** MIR103-1 (includes EG:406895), MT1L, **PDCD10,** PGLYRP1, **RAD51L3, RAP2B,** SLC27A2, **SOX21, SPR**, TNFSF8 (includes EG:944), YWHAZ	16	13	Cell Death, Post-Translational Modification, Molecular Transport
12	APP, BACE2, CCDC99, CCNK, CDC14B, CHCHD3, **CPLX1,** DBF4, DNAJC11, DRAM1, **EN1,** FAM173A, **FKBP6,** HJURP, HPCA, HTT, IMMT, **LANCL1,** MAN1A1, **MAN2A1,** MIR124, **MTX2,** MTX1 (includes EG:4580), **ORC6L, PHEX,** POU2AF1, **RER1,** RNF20, **SDS,** SERPINA3K, SESN1, **SLC22A4 (includes EG:6583)**, SP1, **TACC3,** TP53	15	12	Neurological Disease, Inflammatory Disease, Cellular Compromise
13	**ARSE, ART4, CFDP1, CHL1, CLCN5**, FOXH1, GRB2, IK, LAX1, MLL4, PDLIM7, POP1, **POP4,** POP7, **PRG4 (includes EG:10216),** PTPN22, PTPRA, PTPRN2, RPP14, RPP21, RPP30, RPP38, RPP40, RPPH1 (includes EG:85495), **RPS25,** SHB, **SLC25A3,** SMAD3, SRP14, TNK2, TRAT1, UPF2, WWP2, ZAP70, **ZNF140**	13	10	Cell-To-Cell Signaling and Interaction, Cell-mediated Immune Response, Cellular Development
14	AKAP, AKAP1, AKAP2, AKAP4, AKAP6, AKAP7, **AKAP10,** AKAP11, amino acids, CHM, FUT1, **FUT2,** GSK3A, **PCGF3,** PDZK1, PKAr, **PMVK,** PRKACB, PRKAG1, PRKAR1A, PRKAR1B, PRKAR2B, PRKX, PTPN7, **RABGGTA,** RABGGTB, RNF2, SEC1, **SF3A2,** SF3B2, SF3B3, **SF3B4,** SMNDC1, SNRPB2, **STK19**	10	8	Amino Acid Metabolism, Post-Translational Modification, Small Molecule Biochemistry
15	**CKS1A,** PIN1	2	1	Cell Cycle, Cellular Assembly and Organization, Cellular Development
16	UBXN7, **WDR11 (includes EG:55717)**	2	1	Cancer, Neurological Disease
17	**CRYGS,** D-galactose	2	1	Organ Development, Visual System Development and Function, Tissue Development
18	**CLIC3,** MAPK15	2	1	Molecular Transport, Genetic Disorder, Neurological Disease
19	**BRD3,** SRF, ZNF143	2	1	Gene Expression, Cardiovascular Disease, Cellular Growth and Proliferation
20	CA2, **SLC4A8,** SLC9A3R1, sodium	1	1	Molecular Transport, Cardiovascular Disease, Genetic Disorder
21	BCHE, **CLPS,** GALACTOLIPASE, PNLIP	1	1	Small Molecule Biochemistry, Lipid Metabolism, Molecular Transport
