# Supplementary material for: Protein-Binding Microarray Analysis of Tumor Suppressor AP2α Target Gene Specificity
Source: PLoS One. 2011 Aug 18;6(8):e22895. doi: 10.1371/journal.pone.0022895 (PMC3158074; doi:10.1371/journal.pone.0022895)
Supplement: Table S3 — Human genomic sequences cloned into the eGFP reporter plasmid for experimental validation. Highest AP2α weight matrix score are indicated in the table and corresponding sequences are underlined. (PDF) [file pone.0022895.s008.pdf]

Table S3: DNA regulatory sequences identified as potential AP2α target sequences using the PBM and selected for experimental validation

Gene name	length	AP2α weight matrix score	Sequence
OCM	914 bp	27 (non significant)	5'–<br>GATCCCGGATGGGGTTTCACCATCTTGGCCAGGCTGGTCTCAAACTCCTGACCTCGTGATCCACCCACCTCAGCCTCCCAAAGTGCTAGGATTACAGGCGTGAGCCACTGCGTCTGGCCGCCTGGCCTACATTTTGAT<br>TTTGTGTTGCCTCGCTGGAGGGGCAAAGCTGTGAGGAAATCCCACCCCCGCCCCACGTCCCCTCTACCTTGGCATTCCTGGAGCGCTGCTGCAATGTCATCAGCACTGAGCACGTCCGTGATGCTCATTTTCTACCTA<br>CTCACACAGAATAAACGAGAGGCGATAAGCCACAAACAGGAACGTGCACATCCAGGGGAAACACATCTTCCCAGGCCCACTGAAACTGTATGTGTGTCTAGATTTTTTTTTTTTTTTTTTGAGATGGAGTCTCACTCT<br>GTCACCCAGGCTGGAGTGCAGTGGCACAATCTTGGCTCACTGCAACCTCCACCTCCTGGGCTCAAGTGATTCTCCTGCCTCAGCCTTCCGAGTAGCTGGGATTACAGGTGCGCACTACCAGGCCTGGCTGATTTTTGT<br>GTTTTTAGTAGAGATGGGGTTTTGCCATGTTTGCCAGCCTGGTCTCGAGCTCCTGACCTCATGTGATCTGCCTGCCTTGGCCTCACAAACTGCTGGGATTACAGGCATAAGCCACCAAGCCAGGCCTGTCTAGAATTT<br>TTAAAATGTGATAAGCATGGTTTTGTATTAAGTGACGGGACAGAATATCCAAATTAAAACCAGATACCAAAAGGACCCCAGCCCTTTTTGTCTCAGAGCCTTCTTTTGAATATATGCCAAAAATGAAAAAAAAAAACA<br>TGACCCAAAGAAGGATTCAATTGTTTTCTTATAAAAATTATACTGACATAATCAGTCATTTGCATAGGCAGCCGTAAACCATCCCA-3'
GAS2	935 bp	38	5'–<br>GATCCCGTTGGAGCTCTAGATGGCTCCTGGGCCTAGGGGTAAGTTGTTTGTTTTAAAAGACATATTCTACGATAATTAGGAAGAAAAAGAATAAAGAAAAACATAAATGTTTGCCTATTCAAAAACACCTTGCCTTCA<br>AGGGCTCAGACAATGACTGGGGAAAACTCCAATAATTTTTTTAGTGCACTTTCAATGTTATTTTGTATTGAGTATTTGTCTGACTCTTAGCATTGTAATTCAGTAGCAGACATTTCCATGGTCCTTAAAATCCTGATT<br>CAGAGGGGAATAACCCACTGGGAAAAATGGAGCACTAAGCTGAGTCAGTAGGCAGGAACCAGAGGCAGTAATAAGAACTGTAATAGAAGATCAGATGTCAGAGAGAAGCCTCCAGACACATTAAACCAAAGAATTATA<br>CGTAGGTGCTGGTTCCCATAGCACAGCAAAGCTGGGTTCTGGAGATGTTTATTCTTATCACTTCCACAGAAAAAAACAGGACTTCTTTCATAGACTATATGCTAACTCAGACATTTTGCACCAAGTAGAAAAACAATG<br>GGTCATGAAATACAAACCAAAACACTAATTTCACTAGAACCAGGGGTTGATTCAAGGTACTGTAAGAATTCTCGGAATTTAACACTTTTGATATTTTTTCATTTCAGATGCTGCACAACAAACATGTCATGGTCCGTG<br>TGGGAGGAGGCTGGGAAACTTTTGCAGGGTATTTGTTGAAACACGACCCCTGCCGAATGCTGCAGATCTCCCGTGTGGATGGCAAAACATCCCCTATCCAAAGCAAATCTCCAACTCTAAAGGACATGAATCCAGATA<br>ACTACTTGGTGGTCTCTGCCAGTTATAAGGCTAAGAAGGAAATTAAGTGAAACAAATTGGTCATGACAAGGGGACCCTCATAATGGCCTGTATCCACTTCTCCCCCA-3'
KLK5	927 bp	45	5'–<br>CCAGCTATGCTGGAGGCTGAGGCACGAGAGTCACTTGAACCCTGGAGGCGGAGGTTGCAGTGGGCCGAGATCACATCACCGCCCTCCAGCCTGGGCGACAGAGCAAGACTCTGTCTCAAATAAATAAATAAACAAACG<br>AACAAGCAGTTTGTTGTACCTTAGTTATACCTAAAAAAAAAAAATGCTGTCAACAAATAGAGCAGAAGTGAAATAAAGGAAAATAAATGGGCCAAGAACTCTAAGGTATATTTGACAAATCATTCAGAACCTTTAAAAA<br>AGAAAGAATCACAGAGGCATAGAAAGACAGGGAGGAACAGGGAGACAGAAACACCTGTGGCCCAAGGAGAACAAAACAAGGCTCCTAAGACAGACAGGAGGAGAGAGAGAGAGAGTGAGTGAGAGACAGACAGAGAAA<br>AAGACAGAGAGAGAGAGACAGAGACAGAGAGACAGAGAGGCGAGAGGGATAGAAAGAGAGAGAGGGGTGGAGAGAGACACGAGATATTGAGAAAGACTCAGAAAGATAGCCGAGGGAGAACCACAGAGAGATGGAAGA<br>AGACTCTGAGAAAAAACCAGAGACAAAGATGGAAAGAGGAGTATCGAGGGTGAACAGACAGTGGTGGAATGAGCAAAATGCAGAGAAGAAAGCAAGCAATCCAGGCGCCAAGAATAGTGACCCAGAGTTGGTGAGAAG<br>CCAGATCCTTAAGGCTGGGGGAGGCAGGGAAGGGGCTGGCCTGGCTTCCGGAGACCCCTCCCCATTCTCCGGGCCAGGGAGGTAGGGAGTGACATTCCGGGACTGGGTGGGGGGTGCTCTGGGGGTGGAGATAGGGGG<br>AGCAGGAGGAGCTATTGCTNAAGGCCCGATAGGCACCTCATTGCCCGGGAATGTGCCCCAGGGAGCAGTGGGTGGTTATAACTCAGGCCCGGTGCCCGG-3'

The human genomic sequences cloned into the eGFP reporter plasmid for exprimental validation are displayed. Sequences haviing the highest binding score using the position weight matrix are underlined.
