# Supplementary material for: Protein-Binding Microarray Analysis of Tumor Suppressor AP2α Target Gene Specificity
Source: PLoS One. 2011 Aug 18;6(8):e22895. doi: 10.1371/journal.pone.0022895 (PMC3158074; doi:10.1371/journal.pone.0022895)
Supplement: Table S5 — Potential AP2α target sequences identified by PBM using the nuclear protein extract from breast tumor tissues. Sequences bound by AP2α protein in nuclear cell extract from cancer breast tissues on hu6k microarray (P<0.05). (PDF) [file pone.0022895.s010.pdf]

Table S5: Potential AP2α target sequences identified by PBM using the nuclear protein extract from breast tumor tissues

Name	ID	Description	logFC	average AP2 binding	P.Value
IGFBP6	NM_002178	insulin-like growth factor binding protein 6	3.34	11.43	0.000
RNPS1	NM_080594	RNA binding protein S1, serine-rich domain	2.51	10.39	0.000
TRDN	IN_76		3.02	10.41	0.000
APG12L	NM_004707	APG12 autophagy 12-like (S. cerevisiae)	2.10	10.94	0.000
PSMF1	NM_006814	proteasome (prosome, macropain) inhibitor subunit 1 (PI31)	2.21	10.78	0.000
BAG5	NM_004873	BCL2-associated athanogene 5	2.06	10.76	0.000
DGUOK	NM_001929	deoxyguanosine kinase	2.11	10.95	0.000
SCYA26	NM_006072	chemokine (C-C motif) ligand 26	2.59	9.51	0.000
P-B	UP_99		3.49	9.86	0.000
SIX6	NM_007374	sine oculis homeobox homolog 6 (Drosophila)	2.87	11.12	0.000
GDF9	NM_005260	growth differentiation factor 9	2.44	11.19	0.000
SCAM-1	IN_142		2.24	10.48	0.000
SLC22A8	NM_004254	solute carrier family 22 (organic anion transporter), member 8	1.75	10.91	0.000
C1QA	NM_015991	complement component 1, q subcomponent, alpha polypeptide	2.08	10.85	0.000
S100A3	NM_002960	S100 calcium binding protein A3	1.88	10.06	0.000
MTAP	NM_002451	methylthioadenosine phosphorylase	1.68	10.86	0.000
CCNB2	NM_004701	cyclin B2	1.69	10.55	0.000
TBX19	NM_005149	T-box 19	2.46	10.62	0.000
RCN2	NM_002902	reticulocalbin 2, EF-hand calcium binding domain	2.38	10.06	0.000
MD-1	NM_004271	lymphocyte antigen 86	1.86	10.66	0.000
ZFP	NM_018651	zinc finger protein 167	1.98	10.42	0.000
SPP1	NM_000582	secreted phosphoprotein 1 (osteopontin, bone sialoprotein I, early T-lymphocyte activation 1)	2.24	10.07	0.000
CRSP3	NM_015979	cofactor required for Sp1 transcriptional activation, subunit 3, 130kDa	1.59	10.68	0.000
S100A9	NM_002965	S100 calcium binding protein A9 (calgranulin B)	1.98	10.90	0.000
SCAMP1	IN_116		1.77	10.62	0.000
PLAB	NM_004864	growth differentiation factor 15	1.87	10.56	0.000
TG	NM_003235	thyroglobulin	2.11	10.97	0.000
SLC9A3R1	NM_004252	solute carrier family 9 (sodium/hydrogen exchanger), isoform 3 regulator 1	1.51	10.62	0.000
LOC55868	NM_018485	G protein-coupled receptor 77	2.44	11.45	0.000
NXF2	IN_132		2.14	10.20	0.000
CCNA2:500	NM_001237	cyclin A2	1.76	10.70	0.000
TSC22	NM_006022	transforming growth factor beta 1 induced transcript 4	1.59	10.53	0.000
CRIPT	NM_014171	postsynaptic protein CRIPT	1.68	10.63	0.000
SIPA1	NM_006747	signal-induced proliferation-associated gene 1	1.72	10.62	0.000
LIPG	NM_006033	lipase, endothelial	1.49	10.81	0.000
EPB41	IN_26		1.77	10.81	0.000
KIAA0923	NM_014021	synovial sarcoma, X breakpoint 2 interacting protein	1.94	10.48	0.000
IMOGN38	IN_143		1.73	9.74	0.000
GLRA2	NM_002063	glycine receptor, alpha 2	1.64	10.88	0.000
OPRM1	NM_000914	opioid receptor, mu 1	1.87	11.26	0.000
GRAP2	NM_004810	GRB2-related adaptor protein 2	1.51	10.55	0.000
DDX18	IN_77		1.57	10.47	0.000
CST	NM_004861	galactose-3-O-sulfotransferase 1	1.61	10.48	0.000
DNAJA3	IN_66		1.52	10.91	0.000
DNASE1L2	NM_001374	deoxyribonuclease I-like 2	1.55	10.82	0.000
U3-55K	NM_004704	RNA, U3 small nucleolar interacting protein 2	1.90	10.67	0.000
ANXA6	IN_37		1.66	10.83	0.000

TNFRSF14	NM_003820	tumor necrosis factor receptor superfamily, member 14 (herpesvirus entry mediator)	2.05	11.06	0.000
NEURL	IN_93		1.47	10.51	0.000
SEC22C	IN_103		1.71	10.55	0.000
KLRG1	NM_005810	killer cell lectin-like receptor subfamily G, member 1	1.93	10.66	0.000
BRPF1	NM_004634	bromodomain and PHD finger containing, 1	1.85	10.66	0.000
HPR6.6	NM_006667	progesterone receptor membrane component 1	2.38	8.99	0.000
KCNK6	NM_004823	potassium channel, subfamily K, member 6	1.45	10.54	0.000
CGR11	IN_54		1.72	9.97	0.000
DNTT	NM_004088	deoxynucleotidyltransferase, terminal	1.78	9.92	0.000
SKD1	NM_004869	vacuolar protein sorting 4B (yeast)	1.61	10.37	0.000
CKTSF1B1	NM_013372	gremlin 1 homolog, cysteine knot superfamily (Xenopus laevis)	1.35	10.61	0.000
MATN4	NM_003833	matrilin 4	1.48	10.71	0.000
RAD51L1	IN_18		1.51	10.50	0.000
SUV39H1	NM_003173	suppressor of variegation 3-9 homolog 1 (Drosophila)	1.96	10.29	0.000
CNOT3	NM_014516	CCR4-NOT transcription complex, subunit 3	1.43	10.63	0.000
KRTHA6	NM_003771	keratin, hair, acidic, 6	1.43	10.84	0.000
TPO	IN_121		1.65	10.41	0.000
GBF1	NM_004193	golgi-specific brefeldin A resistance factor 1	1.40	10.67	0.000
SLC2A6	NM_017585	solute carrier family 2 (facilitated glucose transporter), member 6	2.92	9.87	0.000
U5-100K	NM_004818	DEAD (Asp-Glu-Ala-Asp) box polypeptide 23	1.29	10.54	0.000
CLDN6	NM_021195	claudin 6	1.37	10.71	0.000
CIAO1	NM_004804	WD40 protein Ciao1	1.62	10.56	0.000
CACNA1E	NM_000721	calcium channel, voltage-dependent, alpha 1E subunit	1.45	10.58	0.000
GPT	NM_005309	glutamic-pyruvate transaminase (alanine aminotransferase)	1.49	11.04	0.000
CLDN1	NM_021101	claudin 1	1.47	10.66	0.000
TACSTD2	NM_002353	tumor-associated calcium signal transducer 2	1.57	10.27	0.000
ZNF264	NM_003417		1.36	10.73	0.000
H3FJ	NM_003535	histone 1, H3j	1.79	10.26	0.000
TACR1	NM_001058	tachykinin receptor 1	1.44	10.79	0.000
GPX2	NM_002083	glutathione peroxidase 2 (gastrointestinal)	1.25	10.73	0.000
TNFSF13B	NM_006573	tumor necrosis factor (ligand) superfamily, member 13b	2.55	9.56	0.000
EEF1E1	NM_004280	eukaryotic translation elongation factor 1 epsilon 1	1.38	10.37	0.000
TNK1	NM_003985	tyrosine kinase, non-receptor, 1	1.42	10.69	0.000
EIF2S2	NM_003908	eukaryotic translation initiation factor 2, subunit 2 beta, 38kDa	1.53	10.19	0.000
CD6	NM_006725	CD6 antigen	1.46	10.89	0.000
PARG1	NM_004815	PTPL1-associated RhoGAP 1	1.36	10.62	0.000
NIFS	NM_021100	NFS1 nitrogen fixation 1 (S. cerevisiae)	1.45	10.94	0.000
PP	NM_021129	pyrophosphatase (inorganic)	1.30	11.09	0.000
RXRB	NM_021976	retinoid X receptor, beta	2.24	10.52	0.000
LY95	NM_004828	natural cytotoxicity triggering receptor 2	1.32	10.69	0.000
LOC55885	UP_142		1.50	11.21	0.000
CA1	NM_001738	carbonic anhydrase I	1.42	10.97	0.000
AKR1C3	NM_003739	aldo-keto reductase family 1, member C3 (3-alpha hydroxysteroid dehydrogenase, type II)	1.32	10.36	0.000
STX16	NM_003763	syntaxin 16	1.50	10.56	0.000
UCP4	NM_016593	cytochrome P450, family 39, subfamily A, polypeptide 1	1.64	10.52	0.000
SMC2L1	NM_006444	SMC2 structural maintenance of chromosomes 2-like 1 (yeast)	1.56	10.66	0.000
CDC2L5	NM_003718	cell division cycle 2-like 5 (cholinesterase-related cell division controller)	1.48	10.35	0.000
MAD2L2	NM_006341	MAD2 mitotic arrest deficient-like 2 (yeast)	1.54	10.42	0.000
SLC21A6	IN_109		2.43	9.09	0.000
PR48	IN_150		1.68	10.50	0.000

PAPSS1	NM_005443	3'-phosphoadenosine 5'-phosphosulfate synthase 1	1.29	10.74	0.000
FLT1	NM_002019	fms-related tyrosine kinase 1 (vascular endothelial growth factor/vascular permeability factor receptor)	1.49	10.69	0.000
SAM68	NM_006559	KH domain containing, RNA binding, signal transduction associated 1	1.39	9.92	0.000
E2F3	NM_001949	E2F transcription factor 3	1.15	10.74	0.000
H2AFP	NM_021064	histone 1, H2ag	1.26	10.70	0.000
KIF3A	IN_81		2.42	9.52	0.000
PROZ	NM_003891	protein Z, vitamin K-dependent plasma glycoprotein	1.35	10.47	0.000
PFN2	NM_053024	profilin 2	2.09	10.04	0.000
RAB33A	NM_004794	RAB33A, member RAS oncogene family	1.35	10.47	0.000
PPP1R7	NM_002712	protein phosphatase 1, regulatory subunit 7	1.26	11.24	0.000
SH3BP1	NM_018957	SH3-domain binding protein 1	1.36	10.05	0.000
PEX16	NM_057174	peroxisomal biogenesis factor 16	1.35	10.48	0.000
ADAM28	IN_94		1.76	9.90	0.000
PEX1	NM_000466	peroxisome biogenesis factor 1	1.25	9.28	0.000
GPRK2L	NM_182982	G protein-coupled receptor kinase 4	1.36	10.86	0.000
HSPA2	IN_141		2.08	10.64	0.000
EPB49	NM_001978	erythrocyte membrane protein band 4.9 (dematin)	1.24	10.45	0.000
ORCTL3	NM_004256	solute carrier family 22 (organic cation transporter), member 13	1.24	10.67	0.000
OCLM	IN_68		2.06	10.10	0.000
LTF	UP_128		1.68	10.52	0.000
SGCE	NTF_109		1.31	10.59	0.000
CALR	NM_004343	calreticulin	1.39	10.79	0.000
PPARGC1	NM_013261	peroxisome proliferative activated receptor, gamma, coactivator 1, alpha	2.26	9.51	0.000
MAOB	NM_000898	monoamine oxidase B	1.45	10.69	0.000
NMI	NM_004688	N-myc (and STAT) interactor	1.27	10.47	0.000
JCL-1	NM_177433	melanoma antigen, family D, 2	1.51	9.11	0.000
PLA2G4C	NM_003706	phospholipase A2, group IVC (cytosolic, calcium-independent)	1.36	10.49	0.000
MAP3K14	NM_003954	mitogen-activated protein kinase kinase kinase 14	1.35	10.61	0.000
CDH5	IN_0		1.68	10.59	0.000
TRAP-1	IN_116		1.43	10.54	0.000
MADHIP	NM_004799	zinc finger, FYVE domain containing 9	1.19	10.64	0.000
SCML2	NM_006089	sex comb on midleg-like 2 (Drosophila)	1.31	10.26	0.000
SLBP	NM_006527	stem-loop (histone) binding protein	2.40	10.07	0.000
PPT2	NM_138934	palmitoyl-protein thioesterase 2	1.28	10.88	0.000
EDNRB	NM_003991	endothelin receptor type B	1.24	10.63	0.000
MBD4	NM_003925	methyl-CpG binding domain protein 4	1.33	11.07	0.000
DLC1	NM_006094	deleted in liver cancer 1	1.90	9.81	0.000
GSTTLp28	NM_004832	glutathione S-transferase omega 1	1.42	10.38	0.000
CRH	NM_000756	corticotropin releasing hormone	1.22	10.88	0.000
CDC16	NM_003903	CDC16 cell division cycle 16 homolog (S. cerevisiae)	1.42	10.39	0.000
CES2	NM_198061	carboxylesterase 2 (intestine, liver)	1.47	10.47	0.000
SLC27A4	NM_005094	solute carrier family 27 (fatty acid transporter), member 4	1.81	10.21	0.000
GML	NM_002066	GPI anchored molecule like protein	1.45	11.00	0.000
MADH2	IN_124		2.23	10.59	0.000
PDIR	NM_006810	for protein disulfide isomerase-related	2.50	9.54	0.000
ARHGEF4	IN_98		1.77	9.97	0.000
SNRPC	NM_003093	small nuclear ribonucleoprotein polypeptide C	1.21	10.41	0.000
MAP7	NM_003980	microtubule-associated protein 7	1.37	10.61	0.000
AP1G2	NM_003917	adaptor-related protein complex 1, gamma 2 subunit	1.46	10.48	0.000
PRCP	NM_005040	prolylcarboxypeptidase (angiotensinase C)	1.20	10.44	0.000

PLK	NM_005030	polo-like kinase 1 (Drosophila)	1.13	10.52	0.000
NFAT5	NM_006599	nuclear factor of activated T-cells 5, tonicity-responsive	1.86	10.45	0.000
E48	NM_003695	lymphocyte antigen 6 complex, locus D	1.22	10.82	0.000
LPAAT-delta	NM_020133	1-acylglycerol-3-phosphate O-acyltransferase 4 (lysophosphatidic acid acyltransferase, delta)	1.13	11.02	0.000
SARCOSIN	NM_006063	kelch repeat and BTB (POZ) domain containing 10	1.64	9.45	0.000
AOE372	NM_006406	peroxiredoxin 4	1.96	9.13	0.000
TNS	UP_106		1.28	10.69	0.000
PCYT1B	NM_004845	phosphate cytidylyltransferase 1, choline, beta isoform	1.49	10.30	0.000
CRSP6	NM_004268	cofactor required for Sp1 transcriptional activation, subunit 6, 77kDa	1.09	10.43	0.000
LPL	NM_000237	lipoprotein lipase	1.64	10.18	0.000
ICB-1	NM_004848	chromosome 1 open reading frame 38	1.66	10.29	0.000
WISP1	NM_003882	WNT1 inducible signaling pathway protein 1	1.26	10.89	0.000
SLIT3	IN_40		1.08	10.86	0.000
KIT	NM_000222	v-kit Hardy-Zuckerman 4 feline sarcoma viral oncogene homolog	2.75	10.05	0.000
DKFZP434L1021	IN_132		1.71	10.89	0.000
CD84	NM_003874	CD84 antigen (leukocyte antigen)	1.10	10.56	0.000
CFTR	NM_000492	cystic fibrosis transmembrane conductance regulator, ATP-binding cassette (sub-family C, member 7)	1.62	10.69	0.000
CRYAA	NM_000394	crystallin, alpha A	1.23	10.64	0.000
APEX	NM_017807	O-sialoglycoprotein endopeptidase	1.05	10.76	0.000
FLOT1	NM_005803	flotillin 1	1.12	10.53	0.000
KRT2A	NM_000423	keratin 2A (epidermal ichthyosis bullosa of Siemens)	1.55	10.37	0.000
54TM	NM_020470	Yip1 interacting factor homolog (S. cerevisiae)	1.34	9.23	0.000
ART3	NM_001179	ADP-ribosyltransferase 3	2.10	10.62	0.000
TOM34	NM_006809	translocase of outer mitochondrial membrane 34	1.29	10.49	0.000
SMARCB1	NM_003073	SWI/SNF related, matrix associated, actin dependent regulator of chromatin, subfamily b, member 1	1.63	10.06	0.000
SNX3	NM_003795	sorting nexin 3	1.24	11.02	0.000
GRHPR	NM_012203	glyoxylate reductase/hydroxypyruvate reductase	1.54	10.53	0.000
TJP2	NM_004817	tight junction protein 2 (zona occludens 2)	1.14	10.60	0.000
VNN1	NM_004666	vanin 1	1.32	10.57	0.000
SMARCA5	IN_168		1.54	10.09	0.000
IL11RA	NM_004512	interleukin 11 receptor, alpha	1.18	10.62	0.000
TOP3B	IN_89		1.22	10.63	0.000
LOC56898	UP_135		1.20	11.01	0.000
F11	NM_000128	coagulation factor XI (plasma thromboplastin antecedent)	1.24	10.91	0.000
WISP3	NM_003880	WNT1 inducible signaling pathway protein 3	1.39	10.61	0.000
LOC55871	NM_172003		1.16	10.64	0.000
PROML1	NM_006017	prominin 1	1.18	10.65	0.000
IL12RB2	NM_001559	interleukin 12 receptor, beta 2	1.63	11.03	0.000
TBPL1	NM_004865	TBP-like 1	1.40	10.51	0.000
CCT8	NM_006585	chaperonin containing TCP1, subunit 8 (theta)	2.47	9.00	0.000
HSF2	NM_004506	heat shock transcription factor 2	1.63	10.25	0.000
PMPCB	NM_004279	peptidase (mitochondrial processing) beta	1.40	10.53	0.000
CGA	NM_000735	glycoprotein hormones, alpha polypeptide	1.76	10.37	0.000
PTPRS	NM_002850	protein tyrosine phosphatase, receptor type, S	1.64	10.68	0.000
SPATA2	NM_006038	spermatogenesis associated 2	1.08	10.86	0.000
DR6	NM_014452	tumor necrosis factor receptor superfamily, member 21	1.85	10.60	0.000
SYN47	IN_76		1.54	10.28	0.000
GCP2	NM_006659	tubulin, gamma complex associated protein 2	1.52	10.30	0.000
MCM3:500	NM_002388	MCM3 minichromosome maintenance deficient 3 (S. cerevisiae)	1.50	10.44	0.000
P2RX7	NM_002562	purinergic receptor P2X, ligand-gated ion channel, 7	1.33	10.74	0.000

TEGT	IN_100		1.13	10.74	0.000
CYP27A1	NM_000784	cytochrome P450, family 27, subfamily A, polypeptide 1	1.08	10.92	0.000
hklp2	NM_020242	kinesin-like 7	1.35	10.57	0.000
APOA4	NM_000482	apolipoprotein A-IV	1.30	11.08	0.000
TACC3	IN_116		1.92	9.56	0.000
ZYG	IN_76		2.38	9.29	0.000
PROX1	NM_002763	prospero-related homeobox 1	1.34	10.33	0.000
PRKCH	NM_006255	protein kinase C, eta	1.20	10.81	0.000
STX8	NM_004853	syntaxin 8	1.15	10.57	0.000
PSMB2	IN_19		1.18	10.39	0.000
GNL1	NM_005275	guanine nucleotide binding protein-like 1	1.19	11.08	0.000
SART1	IN_57		1.29	10.46	0.000
RB1:500	NM_000321	retinoblastoma 1 (including osteosarcoma)	1.50	10.18	0.000
DVL2	NM_004422	dishevelled, dsh homolog 2 (Drosophila)	1.29	10.55	0.000
PRCC	IN_20		1.12	10.49	0.000
PSMB5	NM_002797	proteasome (prosome, macropain) subunit, beta type, 5	1.54	10.36	0.000
APG5L	NM_004849	APG5 autophagy 5-like (S. cerevisiae)	1.76	10.60	0.000
PTPN3	NM_002829	protein tyrosine phosphatase, non-receptor type 3	1.09	10.65	0.000
HLA-DMA	NM_006120	major histocompatibility complex, class II, DM alpha	1.22	10.32	0.000
AP1GBP1	IN_118		1.21	10.42	0.000
INA	NM_032727	internexin neuronal intermediate filament protein, alpha	1.02	10.72	0.000
ARPC1A	IN_119		1.47	9.69	0.000
OXCT	NM_000436	3-oxoacid CoA transferase 1	1.35	10.57	0.000
TGFB1	NM_000660	transforming growth factor, beta 1 (Camurati-Engelmann disease)	1.56	10.75	0.000
HSD17B4	NM_000414	hydroxysteroid (17-beta) dehydrogenase 4	1.55	9.77	0.000
SPTLC2	NM_004863	serine palmitoyltransferase, long chain base subunit 2	1.62	10.66	0.000
MFNG	NM_002405	manic fringe homolog (Drosophila)	1.17	10.71	0.000
CAMK4	NM_001744	calcium/calmodulin-dependent protein kinase IV	1.23	10.77	0.000
GPSN2	NM_138501	glycoprotein, synaptic 2	1.52	10.62	0.000
NME1	NM_000269	non-metastatic cells 1, protein (NM23A) expressed in	1.21	10.72	0.000
EDF1	IN_106		1.21	10.65	0.000
CACNB1	NM_000723	calcium channel, voltage-dependent, beta 1 subunit	1.28	10.50	0.000
FMR2	NM_002025	fragile X mental retardation 2	1.16	10.61	0.000
FXYD7	NM_022006	FXYD domain containing ion transport regulator 7	1.12	10.56	0.000
STC1	NM_003155	stanniocalcin 1	1.65	10.04	0.000
SERPINB3	NM_006919	serine (or cysteine) proteinase inhibitor, clade B (ovalbumin), member 3	1.31	10.84	0.000
PSTPIP2	IN_65		1.20	10.61	0.000
GTF3C4	NM_012204	general transcription factor IIIC, polypeptide 4, 90kDa	1.00	10.52	0.000
GPCR150	NM_014373	G protein-coupled receptor 160	1.26	11.10	0.000
KIAA0737	NM_014828		1.79	9.83	0.000
OAZ3	NM_016178	ornithine decarboxylase antizyme 3	1.78	9.99	0.000
IFNA1	NM_024013	interferon, alpha 1	1.00	10.68	0.000
JRKL	NM_003772	jerky homolog-like (mouse)	1.53	10.21	0.000
PRDM1	NM_001198	PR domain containing 1, with ZNF domain	1.09	10.66	0.000
U3-55K	NM_004704	RNA, U3 small nucleolar interacting protein 2	1.43	10.81	0.000
CLN2	NM_000391	ceroid-lipofuscinosis, neuronal 2, late infantile (Jansky-Bielschowsky disease)	1.08	11.13	0.000
CDC2:500	NM_001786	cell division cycle 2, G1 to S and G2 to M	1.83	10.40	0.000
PIN4:304	UP_5		1.20	10.68	0.000
PDCD8	NM_004208	programmed cell death 8 (apoptosis-inducing factor)	2.03	10.43	0.000
PTGER3	NM_000957	prostaglandin E receptor 3 (subtype EP3)	1.86	10.31	0.000

ST3GALVI	NM_006100	sialyltransferase 10 (alpha-2,3-sialyltransferase VI)	2.10	9.62	0.000
HSP105B	NM_006644	heat shock 105kDa/110kDa protein 1	1.51	8.95	0.000
RTN1	NM_021136	reticulon 1	0.99	11.01	0.000
UBE2A	NM_003336	ubiquitin-conjugating enzyme E2A (RAD6 homolog)	1.04	10.35	0.000
LOC51678	NM_016447	membrane protein, palmitoylated 6 (MAGUK p55 subfamily member 6)	0.98	10.69	0.000
LILRA1	NM_006863	leukocyte immunoglobulin-like receptor, subfamily A (with TM domain), member 1	1.56	9.86	0.000
MAP3K3	NTF_151		1.41	10.26	0.000
NSF	UP_53		1.09	10.99	0.000
ARPC4	NM_005718	actin related protein 2/3 complex, subunit 4, 20kDa	1.28	11.35	0.000
VAMP1	NM_016830	vesicle-associated membrane protein 1 (synaptobrevin 1)	0.93	11.04	0.000
APOF	NM_001638	apolipoprotein F	1.03	11.01	0.000
ECH1	NM_001398	enoyl Coenzyme A hydratase 1, peroxisomal	1.01	10.57	0.000
ARHI	NM_004675	ras homolog gene family, member I	0.96	10.29	0.000
PRH2	NM_006250	proline-rich protein HaeIII subfamily 1	1.05	10.50	0.000
B4GALT2	NM_003780	UDP-Gal:betaGlcNAc beta 1,4- galactosyltransferase, polypeptide 2	1.34	10.65	0.000
BFSP1	NM_001195	beaded filament structural protein 1, filensin	1.03	10.72	0.000
E2F2:500	NM_004091	E2F transcription factor 2	1.55	10.52	0.000
POU2F1	NM_002697	POU domain, class 2, transcription factor 1	1.95	10.18	0.000
ZNF265	NM_005455	zinc finger protein 265	1.08	10.32	0.000
T-STAR	NM_006558	KH domain containing, RNA binding, signal transduction associated 3	1.14	9.84	0.000
ZNF76	NM_003427	zinc finger protein 76 (expressed in testis)	1.41	10.98	0.000
BAIAP3	NM_003933	BAI1-associated protein 3	1.18	11.04	0.000
ERCC4	NM_005236	excision repair cross-complementing rodent repair deficiency, complementation group 4	1.11	10.42	0.000
PRPSAP1	NM_002766	phosphoribosyl pyrophosphate synthetase-associated protein 1	1.12	10.96	0.000
ORCTL4	NM_004803	solute carrier family 22 (organic cation transporter), member 14	1.29	10.36	0.000
SERPINA4	IN_9		1.41	9.75	0.000
MDM2	NM_002392	Mdm2, transformed 3T3 cell double minute 2, p53 binding protein (mouse)	1.47	10.58	0.000
HSPE1	NM_002157	heat shock 10kDa protein 1 (chaperonin 10)	1.15	10.69	0.000
MAP4K5	NM_006575	mitogen-activated protein kinase kinase kinase kinase 5	1.63	9.80	0.000
MASP2	NM_006610	mannan-binding lectin serine protease 2	1.07	10.85	0.000
LOC51146	NM_016161	alpha-1,4-N-acetylglucosaminyltransferase	1.51	10.65	0.000
SILV	NM_006928	silver homolog (mouse)	1.28	10.29	0.000
ZNF259	NM_003904	zinc finger protein 259	1.09	10.79	0.000
RGS11	NM_003834	regulator of G-protein signalling 11	1.12	10.27	0.000
BAG3	NM_004281	BCL2-associated athanogene 3	1.08	11.15	0.000
SELENBP1	IN_54		1.42	10.41	0.000
MTMR4	IN_91		1.06	10.56	0.000
HAPIP	NM_003947	huntingtin-associated protein interacting protein (duo)	1.70	10.37	0.000
ATP6N1A	NM_005177	ATPase, H+ transporting, lysosomal V0 subunit a isoform 1	1.85	10.41	0.000
NRXN3	NM_004796	neurexin 3	1.42	10.59	0.000
LTA4H	NM_000895	leukotriene A4 hydrolase	1.08	10.47	0.000
EDN3	NM_000114	endothelin 3	1.13	10.74	0.000
INPP5A	IN_19		1.51	10.71	0.000
MPDU1	NM_004870	mannose-P-dolichol utilization defect 1	0.97	10.29	0.000
CNOT4	NM_013316	CCR4-NOT transcription complex, subunit 4	0.92	10.97	0.000
MADH9	NM_005905	SMAD, mothers against DPP homolog 9 (Drosophila)	1.05	10.76	0.000
EFNB3	NM_001406	ephrin-B3	0.96	10.64	0.000
RIPK2	NM_003821	receptor-interacting serine-threonine kinase 2	1.35	10.86	0.000
CCNE2:500	NM_004702	cyclin E2	1.29	10.21	0.000
PRSS8	NM_002773	protease, serine, 8 (prostasin)	1.06	10.55	0.000

NAP1L3	NM_004538	nucleosome assembly protein 1-like 3	1.54	10.70	0.000
SIGLEC5	NM_003830	sialic acid binding Ig-like lectin 5	1.24	10.51	0.000
STOML1	NM_004809	stomatin (EPB72)-like 1	1.18	10.58	0.000
CDYL	NM_004824	chromodomain protein, Y-like	0.86	10.55	0.000
NR6A1	NM_001489	nuclear receptor subfamily 6, group A, member 1	0.96	10.51	0.000
BST2	NM_004335	bone marrow stromal cell antigen 2	1.49	10.37	0.000
HNRPAB	NTF_4		1.27	10.76	0.000
CSX	NM_004387	NK2 transcription factor related, locus 5 (Drosophila)	1.03	10.78	0.000
GFRA4	NM_022139	GDNF family receptor alpha 4	1.66	9.85	0.000
BCKDHA	NM_000709	branched chain keto acid dehydrogenase E1, alpha polypeptide (maple syrup urine disease)	1.10	10.94	0.000
LOC51270	NM_016521	E2F-like protein	1.08	10.88	0.000
PKP2	UP_13		0.85	10.72	0.000
TBX4	NM_018488	T-box 4	1.28	10.51	0.000
MLANA	NM_005511	melan-A	0.89	10.73	0.000
PSG6	NM_002782	pregnancy specific beta-1-glycoprotein 6	0.98	10.62	0.000
TNFSF10	NM_003810	tumor necrosis factor (ligand) superfamily, member 10	1.12	10.41	0.000
CLIC1	NM_001288	chloride intracellular channel 1	1.10	10.02	0.000
STX10	NM_003765	syntaxin 10	1.32	10.51	0.000
RAP1GA1	NM_002885	RAP1, GTPase activating protein 1	1.72	9.98	0.000
NMT2	IN_106		1.11	10.43	0.000
NEUROD1	NM_002500	neurogenic differentiation 1	0.87	10.92	0.000
TAF2F	NM_005642	TAF7 RNA polymerase II, TATA box binding protein (TBP)-associated factor, 55kDa	1.19	10.19	0.000
SLC7A7	UP_66		1.33	10.46	0.000
VAMP4	NM_003762	vesicle-associated membrane protein 4	1.62	10.11	0.000
SIX2	NM_016932	sine oculis homeobox homolog 2 (Drosophila)	1.22	10.96	0.000
HE3-ALPHA	NM_006683	family with sequence similarity 12, member A	1.28	9.77	0.000
CDC23	NM_004661	CDC23 (cell division cycle 23, yeast, homolog)	1.52	10.63	0.000
PLAA	NM_004253	phospholipase A2-activating protein	1.34	10.35	0.000
REPRIMO	NM_019845	candidate mediator of the p53-dependent G2 arrest	1.07	10.96	0.000
MRC1	NM_002438	mannose receptor, C type 1	1.09	11.04	0.000
GLE1L	NM_001499	GLE1 RNA export mediator-like (yeast)	0.94	10.87	0.000
PRSS3	NM_002771	protease, serine, 3 (mesotrypsin)	1.02	10.60	0.000
NOL4	IN_106		1.32	10.24	0.000
C8A	NM_000562	complement component 8, alpha polypeptide	1.13	11.13	0.000
ZNF9	NM_003418	zinc finger protein 9 (a cellular retroviral nucleic acid binding protein)	1.06	11.04	0.000
PPYR1	NM_005972	pancreatic polypeptide receptor 1	0.98	10.78	0.000
GML	NM_002066	GPI anchored molecule like protein	1.03	10.93	0.000
RASGRP2	NM_005825	RAS guanyl releasing protein 2 (calcium and DAG-regulated)	1.47	10.17	0.000
NAP1	NM_004851	napsin A aspartic peptidase	1.10	10.29	0.000
SSA1	NM_003141	Sjogren syndrome antigen A1 (52kDa, ribonucleoprotein autoantigen SS-A/Ro)	1.20	10.68	0.000
PFKFB3	NM_004566	6-phosphofructo-2-kinase/fructose-2,6-biphosphatase 3	1.06	10.73	0.000
ZID	NM_006626	zinc finger protein 482	1.44	9.36	0.000
ITGB4BP	NM_002212	integrin beta 4 binding protein	1.11	10.63	0.000
PDE6B	IN_3		1.89	10.06	0.000
ABCB11	NM_003742	ATP-binding cassette, sub-family B (MDR/TAP), member 11	1.06	10.55	0.000
NID	NM_002508	nidogen (enactin)	1.03	10.92	0.000
SNFT	NM_018664	Jun dimerization protein p21SNFT	1.30	10.18	0.000
CRADD	NM_003805	CASP2 and RIPK1 domain containing adaptor with death domain	1.07	10.78	0.000
BCAS2	NM_005872	breast carcinoma amplified sequence 2	1.26	9.15	0.000
CAMK1	NM_003656	calcium/calmodulin-dependent protein kinase I	0.91	10.98	0.000

AZGP1	NM_001185	alpha-2-glycoprotein 1, zinc	1.01	10.79	0.000
LATS1	NM_004690	LATS, large tumor suppressor, homolog 1 (Drosophila)	0.94	10.44	0.000
MRG15	IN_57		1.33	9.49	0.000
MTF1	NM_005955	metal-regulatory transcription factor 1	1.19	10.71	0.000
IL18	NM_001562	interleukin 18 (interferon-gamma-inducing factor)	1.40	10.18	0.000
CDY2	NM_004825	chromodomain protein, Y-linked, 2	1.16	10.36	0.000
PIGF	NM_002643	phosphatidylinositol glycan, class F	1.38	9.59	0.000
STAG2	IN_65		2.22	10.00	0.000
GALC	NM_000153	galactosylceramidase (Krabbe disease)	0.93	10.51	0.000
RAB4	NM_004578	RAB4A, member RAS oncogene family	1.02	10.73	0.000
RMP	IN_116		0.85	10.59	0.000
K-ALPHA-1	NM_006082	tubulin, alpha, ubiquitous	1.11	8.92	0.000
LOC55831	NM_018447	30 kDa protein	1.46	10.67	0.000
SUDD	NM_003831	RIO kinase 3 (yeast)	1.40	10.96	0.000
ID3	NM_002167	inhibitor of DNA binding 3, dominant negative helix-loop-helix protein	1.04	10.54	0.000
NCK2	NM_003581	NCK adaptor protein 2	0.89	10.37	0.000
GMFG	NM_004877	glia maturation factor, gamma	0.94	10.45	0.000
EXTL1	NM_004455	exostoses (multiple)-like 1	0.94	10.53	0.000
OCM	NM_006188	oncomodulin	1.17	11.32	0.000
NNMT	NM_006169	nicotinamide N-methyltransferase	0.84	10.92	0.000
ANPEP	NM_001150	alanyl (membrane) aminopeptidase (aminopeptidase N, aminopeptidase M, microsomal aminopeptidase, CD13, p150)	1.05	11.08	0.000
MST1R	NM_002447	macrophage stimulating 1 receptor (c-met-related tyrosine kinase)	1.30	11.02	0.000
FUBP1	IN_119		1.03	10.23	0.000
TAF2H	NM_006284	TAF10 RNA polymerase II, TATA box binding protein (TBP)-associated factor, 30kDa	1.24	10.67	0.000
PIASX-BETA	IN_90		0.89	10.51	0.000
FBN1	NM_000138	fibrillin 1 (Marfan syndrome)	1.07	10.65	0.000
MAN1	NM_014319	integral inner nuclear membrane protein	1.05	10.68	0.000
BLM	NM_000057	Bloom syndrome	0.95	10.74	0.000
CHST2	NM_004267	carbohydrate (N-acetylglucosamine-6-O) sulfotransferase 2	0.98	10.30	0.000
SLC25A12	IN_80		1.09	10.74	0.000
RPP20	NM_005837	processing of precursor 7, ribonuclease P subunit (S. cerevisiae)	0.94	9.97	0.000
RAB11A	NM_004663	RAB11A, member RAS oncogene family	1.33	10.97	0.000
DLEU1	NM_005887	deleted in lymphocytic leukemia, 1	1.65	8.64	0.000
GROS1	NM_022356	leucine proline-enriched proteoglycan (leprecan) 1	0.95	10.57	0.000
LOC55871	UP_141		0.77	10.79	0.000
GOLGA1	NM_002077	golgi autoantigen, golgin subfamily a, 1	0.94	10.78	0.000
TAGLN	NM_003186	transgelin	0.82	10.69	0.000
LOC80298	NM_025198	transcription termination factor-like protein	0.95	10.38	0.000
ATP6B1	NM_001692	ATPase, H+ transporting, lysosomal 56/58kDa, V1 subunit B, isoform 1 (Renal tubular acidosis with deafness)	1.22	10.78	0.000
USP18	NM_017414	ubiquitin specific protease 18	1.26	10.21	0.000
BAZ1B	NM_023005	bromodomain adjacent to zinc finger domain, 1B	1.24	10.55	0.000
MBD2	NM_003927	methyl-CpG binding domain protein 2	1.03	10.93	0.000
RAB27A	NM_183236	RAB27A, member RAS oncogene family	1.21	10.44	0.000
CAT	NM_001752	catalase	1.76	10.07	0.000
SF3A3	UP_62		1.40	9.96	0.000
SMS	NM_004595	spermine synthase	1.27	9.81	0.000
TECTA	NM_005422	tectorin alpha	1.42	10.50	0.000
SSR3	NM_007107	signal sequence receptor, gamma (translocon-associated protein gamma)	1.03	10.84	0.000
BCDO	IN_120		0.95	10.62	0.000
SERPINI1	NM_005025	serine (or cysteine) proteinase inhibitor, clade I (neuroserpin), member 1	1.17	11.02	0.000

PLOD3	NM_001084	procollagen-lysine, 2-oxoglutarate 5-dioxygenase 3	1.25	10.85	0.000
NEU3	NM_006656	sialidase 3 (membrane sialidase)	1.15	9.29	0.000
PRTN3	NM_002777	proteinase 3 (serine proteinase, neutrophil, Wegener granulomatosis autoantigen)	1.20	10.92	0.000
SERPINB7	NM_003784	serine (or cysteine) proteinase inhibitor, clade B (ovalbumin), member 7	1.24	10.61	0.000
CALM1	NM_006888	calmodulin 1 (phosphorylase kinase, delta)	1.37	10.51	0.000
MT3	NM_005954	metallothionein 3 (growth inhibitory factor (neurotrophic))	1.00	11.20	0.000
SLC12A6	NM_005135	solute carrier family 12 (potassium/chloride transporters), member 6	1.10	10.53	0.000
DYRK1B	NM_004714	dual-specificity tyrosine-(Y)-phosphorylation regulated kinase 1B	1.09	10.41	0.000
ATP6H	NM_003945	ATPase, H+ transporting, lysosomal 9kDa, V0 subunit e	1.42	10.32	0.000
SNX10	NM_013322	sorting nexin 10	0.82	10.81	0.000
KCNK3	IN_19		0.96	11.16	0.000
ENPEP	NM_001977	glutamyl aminopeptidase (aminopeptidase A)	1.15	10.92	0.000
LTC4S	NM_000897	leukotriene C4 synthase	0.83	10.55	0.000
RGS9	NM_003835	regulator of G-protein signalling 9	1.01	10.52	0.000
PLAA	NM_004253	phospholipase A2-activating protein	0.91	10.37	0.000
KRTHA8	NM_006771	keratin, hair, acidic, 8	0.94	10.44	0.000
TMOD	NM_003275	tropomodulin 1	1.02	9.72	0.000
GALR2	NM_003857	galanin receptor 2	1.02	10.49	0.000
SRCAP	NM_006662	Snf2-related CBP activator protein	1.45	10.08	0.000
PPP1R5	NM_005398	protein phosphatase 1, regulatory (inhibitor) subunit 3C	0.94	10.77	0.000
GALNT4	NM_003774	UDP-N-acetyl-alpha-D-galactosamine:polypeptide N-acetylgalactosaminyltransferase 4 (GalNAc-T4)	1.59	10.39	0.000
RES4-22	NM_003704	chromosome 4 open reading frame 8	1.26	10.22	0.000
FGF17	NM_003867	fibroblast growth factor 17	1.75	10.03	0.000
SNAP23	IN_106		0.89	10.58	0.000
VCY	NM_004679	variable charge, Y-linked	1.07	10.61	0.000
FBXO7	NM_012179	F-box protein 7	0.91	10.80	0.000
M83	NM_021259	transmembrane protein 8 (five membrane-spanning domains)	0.92	10.25	0.000
UBE2D2	NM_003339	ubiquitin-conjugating enzyme E2D 2 (UBC4/5 homolog, yeast)	1.37	9.89	0.000
K6HF	NM_004693	cytokeratin type II	1.19	10.80	0.000
HSA6591	NM_014487	zinc finger protein 330	0.90	10.99	0.000
NAKAP95	NM_014371	A kinase (PRKA) anchor protein 8-like	0.88	10.84	0.000
GABRR2	NM_002043	gamma-aminobutyric acid (GABA) receptor, rho 2	0.96	10.50	0.000
SLC7A6	NM_003983		0.87	10.67	0.000
UBE2H	NM_003344	ubiquitin-conjugating enzyme E2H (UBC8 homolog, yeast)	0.83	11.03	0.000
LTB4R	NM_181657	leukotriene B4 receptor	1.22	10.18	0.000
SLC20A1	NM_005415	solute carrier family 20 (phosphate transporter), member 1	1.64	10.21	0.000
MYG1	NM_021640	chromosome 12 open reading frame 10	0.97	10.30	0.000
SUCLA2	NM_003850	succinate-CoA ligase, ADP-forming, beta subunit	0.95	10.56	0.000
OAS3	NM_006187	2'-5'-oligoadenylate synthetase 3, 100kDa	0.83	11.06	0.000
EIF3S2	NM_003757	eukaryotic translation initiation factor 3, subunit 2 beta, 36kDa	1.03	10.44	0.000
SCA2	NM_002973	spinocerebellar ataxia 2 (olivopontocerebellar ataxia 2, autosomal dominant, ataxin 2)	0.79	10.96	0.000
NDUFC1	NM_002494	NADH dehydrogenase (ubiquinone) 1, subcomplex unknown, 1, 6kDa	1.08	10.20	0.000
UBQLN1	NM_013438	ubiquilin 1	1.02	10.58	0.000
CHRAC1	NM_017444	chromatin accessibility complex 1	0.97	10.81	0.000
CD2	NM_001767	CD2 antigen (p50), sheep red blood cell receptor	1.49	10.04	0.000
CD164	NM_006016	CD164 antigen, sialomucin	1.55	10.22	0.000
CD97	NM_001784	CD97 antigen	1.03	9.57	0.000
DSCR2	NM_003720	Down syndrome critical region gene 2	1.11	10.60	0.000
USP6	UP_101		1.04	10.99	0.000
FLJ23476	NM_024640	ischemia/reperfusion inducible protein	1.00	10.35	0.000

PRPS2	NM_002765	phosphoribosyl pyrophosphate synthetase 2	0.96	10.78	0.000
HCK	NM_002110	hemopoietic cell kinase	1.16	10.67	0.000
DUOX1	NM_017434	dual oxidase 1	0.83	10.57	0.000
ABH	NM_006020	alkB, alkylation repair homolog (E. coli)	1.11	10.64	0.000
NDUFB1	NM_004545	NADH dehydrogenase (ubiquinone) 1 beta subcomplex, 1, 7kDa	0.83	10.72	0.000
MCC	IN_29		0.84	10.79	0.000
PRKCD	NTF_19		0.84	10.60	0.000
CREG	NM_003851	cellular repressor of E1A-stimulated genes	0.92	10.67	0.000
LOC51226	NM_016429	coatomer protein complex, subunit zeta 2	0.94	10.26	0.000
DAPK3	NM_001348	death-associated protein kinase 3	0.79	10.65	0.000
AIRE	NM_000383	autoimmune regulator (autoimmune polyendocrinopathy candidiasis ectodermal dystrophy)	1.13	10.63	0.000
IFIT4	NM_001549	interferon-induced protein with tetratricopeptide repeats 4	1.05	10.48	0.000
TP53TG1	NM_007233	TP53 activated protein 1	1.24	10.34	0.000
NDUFB8	NM_005004	NADH dehydrogenase (ubiquinone) 1 beta subcomplex, 8, 19kDa	1.78	9.32	0.000
TAF2N	NM_003487	TAF15 RNA polymerase II, TATA box binding protein (TBP)-associated factor, 68kDa	0.85	10.81	0.000
RSC1A1	NM_006511	regulatory solute carrier protein, family 1, member 1	0.94	10.63	0.000
ASK	NM_006716	activator of S phase kinase	0.98	11.33	0.000
ANKTM1	NM_007332	transient receptor potential cation channel, subfamily A, member 1	1.04	10.50	0.000
SERPINB9	NM_004155	serine (or cysteine) proteinase inhibitor, clade B (ovalbumin), member 9	0.86	10.78	0.000
FHL2	IN_35		1.02	10.98	0.000
GGH	NM_003878	gamma-glutamyl hydrolase (conjugase, folylpolygammaglutamyl hydrolase)	0.82	10.70	0.000
BPLP	NTF_147		1.67	9.63	0.000
GUCY2C	NM_004963	guanylate cyclase 2C (heat stable enterotoxin receptor)	0.99	10.70	0.000
MIC2	NM_002414	CD99 antigen	0.81	10.29	0.000
STAT5A	NM_003152	signal transducer and activator of transcription 5A	1.42	10.62	0.000
RANBP3	NM_003624	RAN binding protein 3	0.99	10.56	0.000
BASP1	IN_86		0.83	10.54	0.000
IL18RAP	NM_003853	interleukin 18 receptor accessory protein	0.72	10.66	0.000
CDW52	NM_001803	CDW52 antigen (CAMPATH-1 antigen)	1.13	9.79	0.000
MAGOH	NM_002370	mago-nashi homolog, proliferation-associated (Drosophila)	1.12	10.87	0.000
SLC16A4	NM_004696	solute carrier family 16 (monocarboxylic acid transporters), member 4	1.03	10.64	0.000
NFYA	NM_002505	nuclear transcription factor Y, alpha	0.92	10.47	0.000
E2F3:500	NM_001949	E2F transcription factor 3	1.35	9.33	0.000
TNFRSF18	NM_004195	tumor necrosis factor receptor superfamily, member 18	1.38	10.52	0.000
PDHA2	NM_005390	pyruvate dehydrogenase (lipoamide) alpha 2	0.94	11.04	0.000
IFRD1	IN_134		1.08	10.18	0.000
CAPN1	NTF_45		0.99	10.45	0.000
IL8	NM_000584	interleukin 8	0.82	10.52	0.000
GNB3	NM_002075	guanine nucleotide binding protein (G protein), beta polypeptide 3	0.79	10.74	0.000
DNAJA2	NM_005880	DnaJ (Hsp40) homolog, subfamily A, member 2	1.29	9.41	0.000
RARS	NM_002887	arginyl-tRNA synthetase	0.80	10.76	0.000
DDC	NM_000790	dopa decarboxylase (aromatic L-amino acid decarboxylase)	0.78	10.76	0.000
ADAM20	NM_003814	a disintegrin and metalloproteinase domain 20	0.96	10.51	0.000
HPRP8BP	NM_004814	U5 snRNP-specific 40 kDa protein (hPrp8-binding)	0.94	10.30	0.000
CUL4B	IN_70		0.76	10.48	0.000
PLA2G4B	NM_005090	phospholipase A2, group IVB (cytosolic)	1.01	10.64	0.000
TRIP15	NM_004236	thyroid receptor interacting protein 15	0.80	11.06	0.000
PPP1CB	NM_002709	protein phosphatase 1, catalytic subunit, beta isoform	1.50	9.91	0.000
TAS2R5	NM_018980	taste receptor, type 2, member 5	0.88	10.93	0.000
LOXL2	NM_002318	lysyl oxidase-like 2	0.81	10.84	0.000

SLC4A4	IN_106		1.04	10.60	0.000
CYP4F3	NM_000896	cytochrome P450, family 4, subfamily F, polypeptide 3	1.10	10.73	0.000
CD22	NM_001771	CD22 antigen	0.96	9.87	0.000
SYBL1	NM_005638	synaptobrevin-like 1	0.86	10.81	0.000
PYY	NM_004160	peptide YY	1.88	9.46	0.000
PTPN11	NM_002834	protein tyrosine phosphatase, non-receptor type 11 (Noonan syndrome 1)	0.78	10.71	0.000
SIRT1	NM_012238	sirtuin (silent mating type information regulation 2 homolog) 1 (S. cerevisiae)	1.51	10.16	0.000
NUBP1	NM_002484	nucleotide binding protein 1 (MinD homolog, E. coli)	0.88	10.80	0.000
PRB1	NM_005039	proline-rich protein BstNI subfamily 1	1.05	10.40	0.000
CCK	NM_000729	cholecystokinin	0.79	10.70	0.000
FSHPRH1	NM_006733	FSH primary response (LRPR1 homolog, rat) 1	0.76	10.61	0.000
PSMB4	NM_002796	proteasome (prosome, macropain) subunit, beta type, 4	1.04	10.60	0.000
RAB2L	NM_004761	ral guanine nucleotide dissociation stimulator-like 2	1.37	9.52	0.000
BLMH	NM_000386	bleomycin hydrolase	0.94	10.72	0.000
POH1	NM_005805	proteasome (prosome, macropain) 26S subunit, non-ATPase, 14	1.12	10.79	0.000
GTF2F2	NM_004128	general transcription factor IIF, polypeptide 2, 30kDa	1.13	10.64	0.000
WASL	NM_003941	Wiskott-Aldrich syndrome-like	0.98	10.79	0.000
LSR7	NM_018559	KIAA1704	1.37	10.11	0.000
CA7	NM_005182	carbonic anhydrase VII	0.82	10.79	0.000
GLE1L	NM_001499	GLE1 RNA export mediator-like (yeast)	0.79	11.14	0.000
P11	NM_006025	26 serine protease	0.75	10.19	0.000
H2BFR	NM_021058	histone 1, H2bj	1.13	10.61	0.000
IDS	NM_000202	iduronate 2-sulfatase (Hunter syndrome)	0.88	10.66	0.000
C1QB	NM_000491	complement component 1, q subcomponent, beta polypeptide	0.81	10.73	0.000
GATA4	IN_19		0.83	10.67	0.000
PHEMX	NM_139022	pan-hematopoietic expression	0.77	10.88	0.000
MYPT1	NM_002480	protein phosphatase 1, regulatory (inhibitor) subunit 12A	1.16	10.60	0.000
SEPP1	NM_005410	selenoprotein P, plasma, 1	0.90	10.09	0.000
GLP1R	NM_002062	glucagon-like peptide 1 receptor	0.78	10.76	0.000
CACNB2	NM_000724	calcium channel, voltage-dependent, beta 2 subunit	0.80	10.61	0.000
SLC6A13	IN_37		0.71	10.80	0.000
GHSR	NM_004122	growth hormone secretagogue receptor	0.81	10.93	0.000
HOXB7	NM_004502	homeo box B7	1.03	10.55	0.000
LOC51283	IN_79		1.10	10.80	0.000
PARVA	NM_018222	parvin, alpha	1.11	10.66	0.000
CPA1	NM_001868	carboxypeptidase A1 (pancreatic)	0.95	10.44	0.000
MPP3	NM_001932	membrane protein, palmitoylated 3 (MAGUK p55 subfamily member 3)	1.32	9.17	0.000
GPX2	NM_002083	glutathione peroxidase 2 (gastrointestinal)	1.00	10.55	0.000
ARPP-21	NM_016300	cyclic AMP-regulated phosphoprotein, 21 kD	1.21	10.06	0.000
NYX	NM_022567	nyctalopin	1.09	10.23	0.000
NEFL	NM_006158	neurofilament, light polypeptide 68kDa	0.93	11.08	0.000
POLA:500	NM_016937	polymerase (DNA directed), alpha	1.46	10.67	0.000
FIBP	NM_004214	fibroblast growth factor (acidic) intracellular binding protein	0.93	10.94	0.000
EDIL3	NM_005711	EGF-like repeats and discoidin I-like domains 3	0.70	10.94	0.000
trp7	NM_020389	transient receptor potential cation channel, subfamily C, member 7	1.04	10.67	0.000
B3GALT2	IN_76		1.08	10.50	0.000
CABP1	IN_106		1.23	9.97	0.000
ARTN	NM_003976	artemin	0.94	10.67	0.000
PKD1	NM_000296	polycystic kidney disease 1 (autosomal dominant)	0.72	10.61	0.000
BLM	NM_000057	Bloom syndrome	1.05	11.07	0.000

HRH3	NM_007232	histamine receptor H3	1.23	9.97	0.000
USP1	NM_003368	ubiquitin specific protease 1	0.86	11.17	0.000
TREH	IN_113		0.88	9.39	0.000
BS69	NM_006624	zinc finger, MYND domain containing 11	1.13	9.96	0.000
PMS2L11	UP_37		0.79	10.84	0.000
IL1RL2	NM_003854	interleukin 1 receptor-like 2	0.77	10.45	0.000
PIG7	IN_106		0.98	10.26	0.000
PRSS16	NM_005865	protease, serine, 16 (thymus)	1.42	9.72	0.000
PKMYT1	NM_004203	membrane-associated tyrosine- and threonine-specific cdc2-inhibitory kinase	0.72	10.33	0.000
NR4A1	NM_002135	nuclear receptor subfamily 4, group A, member 1	0.78	10.57	0.000
CTNNA2	IN_38		0.94	10.62	0.000
MAP1B	NM_032010	microtubule-associated protein 1B	0.88	10.86	0.000
STX4A	NTF_52		0.77	10.87	0.000
PPFIA1	NTF_94		2.04	8.88	0.000
STK11	NM_000455	serine/threonine kinase 11 (Peutz-Jeghers syndrome)	1.63	10.60	0.000
AKR1B1	NM_001628	aldo-keto reductase family 1, member B1 (aldose reductase)	0.88	10.62	0.000
LOC51205	NM_016361	lysophosphatidic acid phosphatase	1.03	10.99	0.000
SCYA25	NM_005624	chemokine (C-C motif) ligand 25	1.30	8.94	0.000
RGN	NM_004683	regucalcin (senescence marker protein-30)	1.21	10.22	0.000
RBMS2	NM_002898	RNA binding motif, single stranded interacting protein 2	0.99	10.10	0.000
ZNF219	NM_016423	zinc finger protein 219	1.35	10.73	0.000
MYH13	NM_003802	myosin, heavy polypeptide 13, skeletal muscle	0.85	10.81	0.000
TOM	NM_012469	chromosome 20 open reading frame 14	0.71	10.63	0.000
SLC13A2	NM_003984	solute carrier family 13 (sodium-dependent dicarboxylate transporter), member 2	0.79	10.39	0.000
FGF8	NM_006119	fibroblast growth factor 8 (androgen-induced)	0.90	10.29	0.000
SRPR	NM_003139	signal recognition particle receptor ('docking protein')	0.70	10.57	0.000
ABCG2	NM_004827	ATP-binding cassette, sub-family G (WHITE), member 2	1.37	10.21	0.000
PDXK	NM_003681	pyridoxal (pyridoxine, vitamin B6) kinase	0.94	10.78	0.000
PSG4	NM_002780	pregnancy specific beta-1-glycoprotein 4	1.25	10.93	0.000
IGFBP1	NM_000596	insulin-like growth factor binding protein 1	0.71	10.98	0.000
TGIF	NM_173209	TGFB-induced factor (TALE family homeobox)	1.24	9.45	0.000
MSH2	NM_000251	mutS homolog 2, colon cancer, nonpolyposis type 1 (E. coli)	1.41	10.53	0.000
RASA3	NTF_87		1.08	8.73	0.000
HSPB1	NM_001540	heat shock 27kDa protein 1	0.99	10.44	0.000
PIN1	NM_006221	protein (peptidyl-prolyl cis/trans isomerase) NIMA-interacting 1	0.77	10.46	0.000
APPBP1	NM_003905	amyloid beta precursor protein binding protein 1, 59kDa	1.20	10.22	0.000
STX3A	NM_004177	syntaxin 3A	0.89	10.77	0.000
DEEPEST	NM_006461	sperm associated antigen 5	1.11	9.20	0.000
RAP2B	NM_002886	RAP2B, member of RAS oncogene family	1.36	9.79	0.000
MAOA	UP_8		1.13	10.75	0.000
CRYM	NM_001888	crystallin, mu	0.82	10.47	0.000
THOP1	NM_003249	thimet oligopeptidase 1	0.81	11.08	0.000
T	NM_003181	T, brachyury homolog (mouse)	1.12	10.16	0.000
MMP2	NM_004530	matrix metalloproteinase 2 (gelatinase A, 72kDa gelatinase, 72kDa type IV collagenase)	0.67	10.90	0.000
LRP2	NM_004525	low density lipoprotein-related protein 2	0.83	10.72	0.000
HAT	NM_004262	airway trypsin-like protease	1.21	9.90	0.000
DSP	NM_004415	desmoplakin	0.88	10.37	0.000
TPM1	UP_121		0.83	10.26	0.000
TIEG	NM_005655	TGFB inducible early growth response	0.94	9.72	0.000
APOBEC2	NM_006789	apolipoprotein B mRNA editing enzyme, catalytic polypeptide-like 2	1.42	9.51	0.000

RBL1	NM_002895	retinoblastoma-like 1 (p107)	0.70	10.59	0.000
PPAP2C	IN_83		1.05	10.29	0.000
SH2D2A	NM_003975	SH2 domain protein 2A	0.96	10.38	0.000
CCNE2	NM_004702	cyclin E2	1.22	10.04	0.000
BMPR1B	IN_48		0.86	9.97	0.000
TLOC1	NM_003262	translocation protein 1	0.70	10.57	0.000
GUCY2F	NM_001522	guanylate cyclase 2F, retinal	0.92	10.67	0.000
FLJ10314	IN_127		0.70	10.55	0.000
THPO	NM_000460	thrombopoietin (myeloproliferative leukemia virus oncogene ligand, megakaryocyte growth and development factor)	0.78	10.49	0.000
NDUFS5	NM_004552	NADH dehydrogenase (ubiquinone) Fe-S protein 5, 15kDa (NADH-coenzyme Q reductase)	1.10	10.41	0.000
ALAS2	NM_000032	aminolevulinate, delta-, synthase 2 (sideroblastic/hypochromic anemia)	0.86	10.52	0.000
LAMB3	NM_000228	laminin, beta 3	0.69	10.71	0.000
FGF9	NM_002010	fibroblast growth factor 9 (glia-activating factor)	0.88	10.31	0.000
SSA1	NM_003141	Sjogren syndrome antigen A1 (52kDa, ribonucleoprotein autoantigen SS-A/Ro)	1.00	9.80	0.000
PIG11	IN_77		0.77	10.80	0.000
PCMT1	NM_005389	protein-L-isoaspartate (D-aspartate) O-methyltransferase	0.80	10.52	0.000
TIAM1	NM_003253	T-cell lymphoma invasion and metastasis 1	1.11	10.66	0.000
ACVR2	IN_10		0.82	10.87	0.000
IGF2R	NM_000876	insulin-like growth factor 2 receptor	1.20	10.49	0.000
CADPS	IN_83		0.80	10.57	0.000
T-STAR	NM_006558	KH domain containing, RNA binding, signal transduction associated 3	0.79	11.12	0.000
SHC1	NM_183001	SHC (Src homology 2 domain containing) transforming protein 1	1.03	9.73	0.000
CRYBA2	NM_057093	crystallin, beta A2	1.00	10.33	0.000
HPN	NM_002151	hepsin (transmembrane protease, serine 1)	0.79	10.51	0.000
CYP46	NM_006668	cytochrome P450, family 46, subfamily A, polypeptide 1	1.90	8.78	0.000
TAF1A	NM_005681	TATA box binding protein (TBP)-associated factor, RNA polymerase I, A, 48kDa	0.71	10.50	0.000
CLDN12	IN_90		0.94	10.94	0.000
SCYA27	NM_006664	chemokine (C-C motif) ligand 27	1.65	9.35	0.000
MCM3AP	IN_87		0.81	10.29	0.000
RECQL5	NM_004259	RecQ protein-like 5	1.13	10.09	0.000
PKD2L1	NM_016112	polycystic kidney disease 2-like 1	1.09	10.65	0.000
LY94	NM_004829	natural cytotoxicity triggering receptor 1	0.71	10.56	0.000
MPP1	NM_002436	membrane protein, palmitoylated 1, 55kDa	0.95	10.82	0.000
WARS2	IN_85		1.43	8.99	0.000
SF3B4	NM_005850	splicing factor 3b, subunit 4, 49kDa	0.89	9.46	0.000
HREV107	NM_007069	HRAS-like suppressor 3	1.79	9.13	0.000
CCNI	IN_66		0.87	9.97	0.000
TG	NM_003235	thyroglobulin	1.03	10.70	0.000
DPM2	NM_152690	dolichyl-phosphate mannosyltransferase polypeptide 2, regulatory subunit	0.97	10.20	0.000
VRP	NM_007063	TBC1 domain family, member 8 (with GRAM domain)	1.06	8.77	0.000
NXF1	NM_006362	nuclear RNA export factor 1	0.82	9.94	0.000
POU4F3	NM_002700	POU domain, class 4, transcription factor 3	0.72	10.66	0.000
TMEPAI	NM_020182	transmembrane, prostate androgen induced RNA	0.80	10.82	0.000
ENTPD5	NM_001249	ectonucleoside triphosphate diphosphohydrolase 5	0.83	10.60	0.000
GFRA1	NM_005264	GDNF family receptor alpha 1	0.71	10.82	0.000
STC2	NM_003714	stanniocalcin 2	0.89	10.42	0.000
SIGLEC6	NM_001245	sialic acid binding Ig-like lectin 6	1.10	10.95	0.000
PGM5	UP_34		0.93	8.61	0.000
DD96	NM_005764	membrane-associated protein 17	0.66	10.86	0.000
INPP5A	IN_19		0.77	10.86	0.000

PTOV1	NM_017432	prostate tumor overexpressed gene 1	0.85	10.88	0.000
RPS6KA4	NM_003942	ribosomal protein S6 kinase, 90kDa, polypeptide 4	1.02	10.60	0.000
HDAC2	NM_001527	histone deacetylase 2	0.83	10.20	0.000
LMAN1	NM_005570	lectin, mannose-binding, 1	0.79	10.78	0.000
FLOT2	NM_004475	flotillin 2	1.03	10.34	0.000
MLN51	NM_007359	cancer susceptibility candidate 3	0.76	10.68	0.000
TNF	NM_000594	tumor necrosis factor (TNF superfamily, member 2)	0.83	10.72	0.000
ARHE	NM_005168	ras homolog gene family, member E	0.69	10.81	0.000
HPRP4P	NM_004697	PRP4 pre-mRNA processing factor 4 homolog (yeast)	0.84	10.68	0.000
TCEB3	NM_003198	transcription elongation factor B (SIII), polypeptide 3 (110kDa, elongin A)	0.92	10.47	0.000
APG5L	NM_004849	APG5 autophagy 5-like (S. cerevisiae)	0.99	9.50	0.000
IL4R	NM_000418	interleukin 4 receptor	1.23	10.78	0.000
MEF2C	IN_10		0.74	10.81	0.000
FASTK	NM_025096	FAST kinase	1.73	8.79	0.000
RANBP9	NM_005493	RAN binding protein 9	0.68	10.61	0.000
LCN2	NM_005564	lipocalin 2 (oncogene 24p3)	0.79	10.61	0.000
TPST1	IN_96		0.76	10.85	0.000
C4BPB	NM_000716	complement component 4 binding protein, beta	0.95	10.80	0.000
SFN	NM_006142	stratifin	0.99	10.89	0.000
PMS1	NM_000534	PMS1 postmeiotic segregation increased 1 (S. cerevisiae)	0.78	11.15	0.000
SCO2	NM_005138	SCO cytochrome oxidase deficient homolog 2 (yeast)	1.07	10.73	0.000
PHYH	NM_006214	phytanoyl-CoA hydroxylase (Refsum disease)	0.96	10.63	0.000
EIF3S3	IN_85		0.99	10.28	0.000
CSN10	NM_005212	casein kappa	0.74	10.72	0.000
GNAZ	NM_002073	guanine nucleotide binding protein (G protein), alpha z polypeptide	1.09	10.92	0.000
STAT2	NM_005419	signal transducer and activator of transcription 2, 113kDa	0.88	10.85	0.000
SFRS3	IN_139		0.69	10.58	0.000
GCLC	NM_001498	glutamate-cysteine ligase, catalytic subunit	0.85	11.03	0.000
LAMR1	NTF_17		1.19	9.02	0.000
CDC42BPB	NM_006035	CDC42 binding protein kinase beta (DMPK-like)	0.73	10.20	0.000
BST2	NM_004335	bone marrow stromal cell antigen 2	1.10	11.14	0.000
HTR3A	NM_000869	5-hydroxytryptamine (serotonin) receptor 3A	0.98	10.71	0.000
RBBP2	NM_005056	Jumonji, AT rich interactive domain 1A (RBBP2-like)	0.88	10.87	0.000
DLEU2	NM_006021	deleted in lymphocytic leukemia, 2	0.88	10.33	0.000
CD209L	NM_014257	CD209 antigen-like	1.08	9.76	0.000
CACNA1I	NM_021096	calcium channel, voltage-dependent, alpha 1I subunit	0.88	10.17	0.000
P2RXL1	NM_005446	purinergic receptor P2X-like 1, orphan receptor	1.13	10.60	0.000
MTP	NM_000253	microsomal triglyceride transfer protein (large polypeptide, 88kDa)	0.92	10.81	0.000
GLUD1	NTF_37		0.65	10.63	0.000
RBP3	NM_002900	retinol binding protein 3, interstitial	0.75	10.89	0.000
PIK4CB	NM_002651	phosphatidylinositol 4-kinase, catalytic, beta polypeptide	1.42	8.48	0.000
TNFSF9	NM_003811	tumor necrosis factor (ligand) superfamily, member 9	0.84	10.74	0.000
TAC1	NM_003182	tachykinin, precursor 1 (substance K, substance P, neurokinin 1, neurokinin 2, neuromedin L, neurokinin alpha, neuropeptide K, neuropeptide gamma)	0.80	10.47	0.000
CLU	NM_001831	clusterin (complement lysis inhibitor, SP-40,40, sulfated glycoprotein 2, testosterone-repressed prostate message 2, apolipoprotein J)	0.84	10.66	0.000
LOC57116	NM_020394	zinc finger protein SBZF3	0.85	10.67	0.000
AMELY	NM_001143	amelogenin, Y-linked	0.64	10.94	0.000
AKAP8	NM_005858	A kinase (PRKA) anchor protein 8	0.92	10.58	0.000
ZMPSTE24	NM_005857	zinc metalloproteinase (STE24 homolog, yeast)	1.08	9.24	0.000
GTF3C1	NM_001520	general transcription factor IIIC, polypeptide 1, alpha 220kDa	0.69	11.09	0.000
SLC22A7	NM_006672	solute carrier family 22 (organic anion transporter), member 7	1.29	8.32	0.000

SQLE	NM_003129	squalene epoxidase	0.67	10.43	0.000
NR0B2	NM_021969	nuclear receptor subfamily 0, group B, member 2	0.84	10.46	0.000
SYNJ1	NM_003895	synaptojanin 1	0.93	10.38	0.000
LOC51194	IN_72		0.80	10.81	0.000
TTID	NM_006790	titin immunoglobulin domain protein (myotilin)	1.25	9.87	0.000
TRAF3	NM_003300	TNF receptor-associated factor 3	0.79	10.55	0.000
HCFC1	NM_005334	host cell factor C1 (VP16-accessory protein)	0.78	10.72	0.000
POLA	NM_016937	polymerase (DNA directed), alpha	0.62	11.09	0.000
MAN1A2	NM_006699	mannosidase, alpha, class 1A, member 2	1.22	9.31	0.000
LBP	NM_004139	lipopolysaccharide binding protein	0.63	10.63	0.000
PTPRH	NM_002842	protein tyrosine phosphatase, receptor type, H	0.70	10.58	0.000
KIAA0175	NM_014791	maternal embryonic leucine zipper kinase	0.65	10.71	0.000
CALM2	NM_001743	calmodulin 2 (phosphorylase kinase, delta)	0.69	10.88	0.000
KRT14	NM_000526	keratin 14 (epidermolysis bullosa simplex, Dowling-Meara, Koebner)	0.79	10.78	0.000
ADAM19	IN_117		0.82	10.82	0.000
DDX11	UP_47		0.65	10.42	0.000
SDHA	NM_004168	succinate dehydrogenase complex, subunit A, flavoprotein (Fp)	0.97	10.62	0.000
SCDGF-B	NM_025208	platelet derived growth factor D	0.76	10.62	0.000
TGN51	NM_006464	trans-golgi network protein 2	1.41	9.59	0.000
TP53	NM_000546	tumor protein p53 (Li-Fraumeni syndrome)	0.69	10.32	0.000
NFE2L2	IN_37		0.63	10.90	0.000
CCNA2:950	NM_001237	cyclin A2	0.81	10.42	0.000
DDR1	NM_001954	discoidin domain receptor family, member 1	0.82	10.20	0.000
AOC3	NM_003734	amine oxidase, copper containing 3 (vascular adhesion protein 1)	0.82	10.69	0.000
RAD17	NM_133343	RAD17 homolog (S. pombe)	0.79	10.38	0.000
ASNS	NM_183356	asparagine synthetase	0.79	10.55	0.000
RFPL2	NM_006605	ret finger protein-like 2	2.36	9.61	0.000
RAI3	NM_003979	retinoic acid induced 3	0.81	10.78	0.000
AP4B1	NM_006594	adaptor-related protein complex 4, beta 1 subunit	1.06	10.06	0.000
BTF3L1	NM_001208	basic transcription factor 3, like 1	0.72	10.78	0.000
LOC51702	NM_016233	peptidyl arginine deiminase, type III	0.64	10.79	0.000
KCNS1	NM_002251	potassium voltage-gated channel, delayed-rectifier, subfamily S, member 1	0.80	10.88	0.000
SFTPA1	NM_005411	surfactant, pulmonary-associated protein A1	0.78	10.78	0.000
ZAK	NM_016653	sterile alpha motif and leucine zipper containing kinase AZK	0.61	11.11	0.000
DNAJC3	NM_006260	DnaJ (Hsp40) homolog, subfamily C, member 3	0.91	10.13	0.000
ISL1	NM_002202	ISL1 transcription factor, LIM/homeodomain, (islet-1)	0.76	10.76	0.000
SMAP	NM_139199	bromodomain containing 8	1.52	9.30	0.000
PTS	NM_000317	6-pyruvoyltetrahydropterin synthase	1.32	9.51	0.000
HHCM	NM_006543		0.88	10.27	0.000
LOC64174	NM_022355	dipeptidase 2	0.77	10.61	0.000
CGRP-RCP	NM_014478	calcitonin gene-related peptide-receptor component protein	0.75	10.70	0.000
SVIL	IN_55		0.85	10.66	0.000
SUPT4H1	NM_003168	suppressor of Ty 4 homolog 1 (S. cerevisiae)	0.63	10.94	0.000
REG1B	NM_006507	regenerating islet-derived 1 beta (pancreatic stone protein, pancreatic thread protein)	1.03	10.59	0.000
CYP11B1	NM_000497	cytochrome P450, family 11, subfamily B, polypeptide 1	0.67	10.72	0.000
GUCA2B	NM_007102	guanylate cyclase activator 2B (uroguanylin)	0.60	10.74	0.000
C1QR	NM_012072	complement component 1, q subcomponent, receptor 1	1.00	10.13	0.000
MYBPC2	NM_004533	myosin binding protein C, fast type	0.73	10.78	0.000
TEB4	IN_76		1.11	9.73	0.000
DHFR:500	NM_000791	dihydrofolate reductase	0.84	10.85	0.000

HNRPF	IN_141		0.76	10.47	0.000
SQSTM1	NM_003900	sequestosome 1	0.80	10.60	0.000
MYOM2:253	NM_003970	myomesin (M-protein) 2, 165kDa	2.40	11.16	0.000
P85SPR	NM_003899	Rho guanine nucleotide exchange factor (GEF) 7	1.55	10.34	0.000
STX5A	NM_003164	syntaxin 5A	1.03	9.96	0.000
CCNA2:500	NM_001237	cyclin A2	0.96	10.70	0.000
TNFAIP2	NM_006291	tumor necrosis factor, alpha-induced protein 2	0.89	10.63	0.000
SERPINB8	NM_002640	serine (or cysteine) proteinase inhibitor, clade B (ovalbumin), member 8	0.80	10.63	0.000
MGAT2	NM_002408	mannosyl (alpha-1,6-)-glycoprotein beta-1,2-N-acetylglucosaminyltransferase	0.84	10.56	0.000
PRKRA	NM_003690	protein kinase, interferon-inducible double stranded RNA dependent activator	0.96	10.99	0.000
MAGEA10	IN_30		0.73	11.05	0.000
TNFRSF6B	NM_003823	tumor necrosis factor receptor superfamily, member 6b, decoy	0.84	10.40	0.000
GSBS	IN_103		1.08	9.68	0.000
SMARCD2	NM_003077	SWI/SNF related, matrix associated, actin dependent regulator of chromatin, subfamily d, member 2	1.30	9.52	0.000
LOC55902	NM_139274	acetyl-Coenzyme A synthetase 2 (ADP forming)	1.23	10.87	0.000
PSMD4	NM_002810	proteasome (prosome, macropain) 26S subunit, non-ATPase, 4	0.63	10.77	0.000
MEIS1	IN_128		0.73	10.68	0.000
SERPINA3	NM_001085	serine (or cysteine) proteinase inhibitor, clade A (alpha-1 antiproteinase, antitrypsin), member 3	0.70	10.36	0.000
ANGPT2	NM_001147	angiopoietin 2	0.80	11.02	0.000
IFNGR1	NM_000416	interferon gamma receptor 1	0.82	10.75	0.000
IFNA21	NM_002175	interferon, alpha 21	0.70	10.74	0.000
PIGC	NM_153747	phosphatidylinositol glycan, class C	1.27	8.99	0.000
ENO2	NM_001975	enolase 2 (gamma, neuronal)	0.72	10.37	0.000
KIAA0905	NM_014933	SEC31-like 1 (S. cerevisiae)	0.57	10.50	0.000
MMP27	NM_022122	matrix metalloproteinase 27	0.98	10.43	0.000
GDF1	NM_001492	growth differentiation factor 1	0.64	10.55	0.000
NDUFA6	NM_002490	NADH dehydrogenase (ubiquinone) 1 alpha subcomplex, 6, 14kDa	0.76	10.43	0.000
BICD1	NM_001714	Bicaudal D homolog 1 (Drosophila)	0.91	10.77	0.000
GRIN2B	NM_000834	glutamate receptor, ionotropic, N-methyl D-aspartate 2B	0.84	10.71	0.000
MPG	NM_002434	N-methylpurine-DNA glycosylase	0.67	10.84	0.000
C3AR1	IN_41		0.73	10.74	0.000
CLCN3	NM_001829	chloride channel 3	0.81	9.91	0.000
CALM3	NM_005184	calmodulin 3 (phosphorylase kinase, delta)	0.61	11.01	0.000
PIK3CB	NM_006219	phosphoinositide-3-kinase, catalytic, beta polypeptide	1.54	9.55	0.000
SDCBP	NM_005625	syndecan binding protein (syntenin)	0.55	11.05	0.000
MYC	NM_002467	v-myc myelocytomatosis viral oncogene homolog (avian)	0.78	11.05	0.000
PVR	NM_006505	poliovirus receptor	1.16	9.24	0.000
KIR2DL4	NM_002255	killer cell immunoglobulin-like receptor, two domains, long cytoplasmic tail, 4	0.73	10.77	0.000
PAPSS2	NM_004670	3'-phosphoadenosine 5'-phosphosulfate synthase 2	0.76	10.34	0.000
HSCP1	NM_021626	serine carboxypeptidase 1	0.63	11.25	0.000
FXYD3	NM_005971	FXYD domain containing ion transport regulator 3	0.66	11.10	0.000
PMF1	NM_007221	polyamine-modulated factor 1	1.28	9.60	0.000
DHFR	NM_000791	dihydrofolate reductase	0.60	10.85	0.000
PEX11B	NM_003846	peroxisomal biogenesis factor 11B	0.71	10.58	0.000
CA6	NM_001215	carbonic anhydrase VI	0.64	10.56	0.000
GUCY1A3	NM_000856	guanylate cyclase 1, soluble, alpha 3	0.62	10.94	0.000
MARK3	NM_002376	MAP/microtubule affinity-regulating kinase 3	0.55	10.83	0.000
AIM2	NM_004833	absent in melanoma 2	0.76	10.24	0.000
NSP1	NM_005490	SH2 domain containing 3A	0.72	10.66	0.000
AKAP9	NM_005751	A kinase (PRKA) anchor protein (yotiao) 9	0.65	10.75	0.000

RBBP1	NM_002892	AT rich interactive domain 4A (RBP1-like)	0.70	10.82	0.000
PPP5C	NM_006247	protein phosphatase 5, catalytic subunit	0.83	10.60	0.000
LGALS9	UP_46		0.64	11.12	0.000
PTPRB	NM_002837	protein tyrosine phosphatase, receptor type, B	1.01	10.35	0.000
ZNF189	NM_197977	zinc finger protein 189	0.72	10.69	0.000
HERC3	NM_014606		0.71	11.12	0.000
CAPZA2	NM_006136	capping protein (actin filament) muscle Z-line, alpha 2	0.70	10.71	0.000
RAD51C	NM_058216	RAD51 homolog C (S. cerevisiae)	1.11	9.92	0.000
RRAS	NM_006270	related RAS viral (r-ras) oncogene homolog	0.93	10.45	0.000
BLVRA	NM_000712	biliverdin reductase A	0.81	10.61	0.000
SFRS8	NM_004592	splicing factor, arginine/serine-rich 8 (suppressor-of-white-apricot homolog, Drosophila)	1.11	10.56	0.000
SFRP4	NM_003014	secreted frizzled-related protein 4	1.05	10.34	0.000
PGD	IN_26		0.81	10.73	0.000
LANCL1	NM_006055	LanC lantibiotic synthetase component C-like 1 (bacterial)	0.70	10.43	0.000
OSMR	NM_003999	oncostatin M receptor	0.68	10.86	0.000
IKBKAP	NM_003640	inhibitor of kappa light polypeptide gene enhancer in B-cells, kinase complex-associated protein	0.73	10.55	0.000
BST1	NM_004334	bone marrow stromal cell antigen 1	0.76	10.53	0.000
GSPT1	NM_002094	G1 to S phase transition 1	0.62	10.55	0.000
CACNA1C	IN_53		0.70	10.57	0.000
VASP	NM_003370	vasodilator-stimulated phosphoprotein	1.45	10.13	0.000
TP63	IN_106		0.64	10.19	0.000
QPCT	NM_012413	glutaminyl-peptide cyclotransferase (glutaminyl cyclase)	0.67	10.71	0.000
LAMA4	NM_002290	laminin, alpha 4	0.70	10.64	0.000
LEFTB	NM_020997	left-right determination, factor B	1.30	8.83	0.000
AQP8	NM_001169	aquaporin 8	0.61	10.99	0.000
DRG2	IN_45		0.70	10.71	0.000
AKR1A1	NM_006066	aldo-keto reductase family 1, member A1 (aldehyde reductase)	1.10	8.33	0.000
FADD	IN_115		0.73	10.65	0.000
SCYD1	NM_002996	chemokine (C-X3-C motif) ligand 1	0.84	11.08	0.000
ABCC3	NM_003786	ATP-binding cassette, sub-family C (CFTR/MRP), member 3	0.92	10.48	0.000
ARHGAP4	NM_001666	Rho GTPase activating protein 4	0.74	10.72	0.000
KRT15	NM_002275	keratin 15	0.75	10.72	0.000
TGFB3	NM_003239	transforming growth factor, beta 3	0.63	10.31	0.000
PNLIPRP2	NM_005396	pancreatic lipase-related protein 2	0.61	10.60	0.000
NOTCH2	IN_40		0.81	10.99	0.000
RBM4	NM_002896	RNA binding motif protein 4	0.64	10.87	0.000
CDK2	NM_001798	cyclin-dependent kinase 2	1.11	10.02	0.000
PAF65A	IN_109		0.60	9.63	0.000
CDC25A:950	NM_001789	cell division cycle 25A	0.85	10.76	0.000
PLA2G2A	NM_000300	phospholipase A2, group IIA (platelets, synovial fluid)	0.61	10.65	0.000
ANK1	NM_000037	ankyrin 1, erythrocytic	0.56	10.88	0.000
TCTE1L	NM_006520	t-complex-associated-testis-expressed 1-like	0.63	11.00	0.000
NAGLU	NM_000263	N-acetylglucosaminidase, alpha- (Sanfilippo disease IIIB)	0.61	10.87	0.000
KCNJ15	IN_14		0.63	11.05	0.000
THY1	IN_90		0.65	10.88	0.000
PSMA1	NM_002786	proteasome (prosome, macropain) subunit, alpha type, 1	0.57	10.44	0.000
GATM	NM_001482	glycine amidinotransferase (L-arginine:glycine amidinotransferase)	0.71	10.65	0.000
BTN3A2	NM_007047	butyrophilin, subfamily 3, member A2	0.84	10.26	0.000
NMB	NM_021077	neuromedin B	0.71	10.71	0.000
DGKE	NM_003647	diacylglycerol kinase, epsilon 64kDa	0.67	10.50	0.000

DLG4	NM_001365	discs, large homolog 4 (Drosophila)	0.90	10.92	0.000
PTHR2	NM_005048	parathyroid hormone receptor 2	0.58	10.71	0.000
SUPT5H	NM_003169	suppressor of Ty 5 homolog (S. cerevisiae)	0.76	10.78	0.000
LSP1	NM_002339	lymphocyte-specific protein 1	0.78	10.58	0.000
BTD	NM_000060	biotinidase	0.80	10.53	0.000
NPM1	IN_17		0.61	10.74	0.000
DR6	NM_014452	tumor necrosis factor receptor superfamily, member 21	1.10	10.11	0.000
CSRP1	NM_004078	cysteine and glycine-rich protein 1	0.83	10.79	0.000
MEST	NM_177525	mesoderm specific transcript homolog (mouse)	0.78	10.54	0.000
CTCF	IN_101		0.82	9.02	0.000
EFNA2	NM_001405	ephrin-A2	1.05	10.63	0.000
JAK2	NM_004972	Janus kinase 2 (a protein tyrosine kinase)	0.87	9.99	0.000
PFN1	NM_005022	profilin 1	0.61	10.73	0.000
MAGEB3	NM_002365	melanoma antigen, family B, 3	0.70	11.04	0.000
CLK2	IN_154		0.94	10.07	0.000
AOX1	IN_6		0.88	10.60	0.000
RODH-4	NM_003708	microsomal NAD+-dependent retinol dehydrogenase 4	0.99	10.35	0.000
BPHL	IN_19		0.81	10.62	0.000
CABP5	NM_019855	calcium binding protein 5	0.70	10.73	0.000
ERO1-L(BETA)	NM_019891	ERO1-like beta (S. cerevisiae)	0.75	10.51	0.000
CAPG	NM_001747	capping protein (actin filament), gelsolin-like	0.74	10.66	0.000
GSTT2	NM_000854	glutathione S-transferase theta 2	0.54	10.71	0.000
HCS	NM_018947	cytochrome c, somatic	0.69	10.56	0.000
SERPINB10	NM_005024	serine (or cysteine) proteinase inhibitor, clade B (ovalbumin), member 10	0.61	10.50	0.000
CD59	NM_000611	CD59 antigen p18-20 (antigen identified by monoclonal antibodies 16.3A5, EJ16, EJ30, EL32 and G344)	1.09	9.96	0.000
CLIC3	NM_004669	chloride intracellular channel 3	0.85	10.35	0.000
PP5395	NM_021732	arginine vasopressin-induced 1	0.60	10.76	0.000
SMPD2	NM_003080	sphingomyelin phosphodiesterase 2, neutral membrane (neutral sphingomyelinase)	1.43	9.05	0.000
MGEA6	NM_005930	meningioma expressed antigen 6 (coiled-coil proline-rich)	0.68	10.45	0.000
SNAPC3	NM_003084	small nuclear RNA activating complex, polypeptide 3, 50kDa	1.29	8.66	0.000
PSEN2	NM_000447	presenilin 2 (Alzheimer disease 4)	1.02	10.35	0.000
RAB7L1	NM_003929	RAB7, member RAS oncogene family-like 1	1.23	10.89	0.000
ASAH	NM_004315	N-acylsphingosine amidohydrolase (acid ceramidase) 1	0.67	10.76	0.000
AP1G2	NM_003917	adaptor-related protein complex 1, gamma 2 subunit	0.62	10.80	0.000
TAF3B2	NM_001519	BRF1 homolog, subunit of RNA polymerase III transcription initiation factor IIIB (S. cerevisiae)	1.29	10.79	0.000
DNCI1	NM_004411	dynein, cytoplasmic, intermediate polypeptide 1	0.67	10.83	0.000
STAC	NTF_53		0.68	10.56	0.000
LOC51216	UP_114		0.66	10.91	0.000
SNW1	NM_012245	SKI interacting protein	0.64	10.13	0.000
SLC1A7	NM_006671	solute carrier family 1 (glutamate transporter), member 7	0.69	10.49	0.000
MINK	NM_015716	misshapen/NIK-related kinase	0.74	10.57	0.000
MMP20	NM_004771	matrix metalloproteinase 20 (enamelysin)	0.62	11.03	0.000
G6E	NM_024123	lymphocyte antigen 6 complex, locus G6E	0.73	10.79	0.000
PRKCL1	NM_002741	protein kinase C-like 1	0.61	10.52	0.000
HAN11	IN_71		0.81	9.28	0.000
SORD	NM_003104	sorbitol dehydrogenase	0.72	10.71	0.000
OPN1LW	NM_020061	opsin 1 (cone pigments), long-wave-sensitive (color blindness, protan)	0.73	10.50	0.000
SLC26A4	NM_000441	solute carrier family 26, member 4	0.57	10.94	0.000
GAD2	NM_000818	glutamate decarboxylase 2 (pancreatic islets and brain, 65kDa)	0.78	10.55	0.000
TRIP12	IN_68		0.57	10.93	0.000

ITIH3	NM_002217	inter-alpha (globulin) inhibitor H3	0.66	11.15	0.000
FOLR3	NM_000804	folate receptor 3 (gamma)	0.92	10.51	0.000
VAMP5	IN_65		0.79	10.28	0.000
KCNA3	NM_002232	potassium voltage-gated channel, shaker-related subfamily, member 3	0.64	10.83	0.000
IF	NM_000204	I factor (complement)	0.62	10.67	0.000
SP100	NM_003113	nuclear antigen Sp100	0.62	10.88	0.000
CALU	NM_001219	calumenin	0.60	10.70	0.000
RPC32	NM_006467	polymerase (RNA) III (DNA directed) polypeptide G (32kD)	0.90	9.94	0.000
NOX3	NM_015718	NADPH oxidase 3	0.80	10.34	0.000
ARHGEF4	IN_98		0.71	10.74	0.000
RAB5C	NM_004583	RAB5C, member RAS oncogene family	1.02	10.31	0.000
MDFI	NM_005586	MyoD family inhibitor	0.66	10.92	0.000
CA4	NM_000717	carbonic anhydrase IV	0.73	10.74	0.000
HDAC8	NM_018486	histone deacetylase 8	1.05	10.48	0.000
CHC1	IN_164		0.89	10.26	0.000
PYCR1	NM_006907	pyrroline-5-carboxylate reductase 1	0.77	9.93	0.000
GAK	NM_005255	cyclin G associated kinase	0.84	10.67	0.000
ACT	NM_020482	four and a half LIM domains 5	1.07	9.62	0.000
COCH	NM_004086	coagulation factor C homolog, cochlin (Limulus polyphemus)	0.59	10.76	0.000
PHEX	NM_000444	phosphate regulating endopeptidase homolog, X-linked (hypophosphatemia, vitamin D resistant rickets)	1.53	9.07	0.000
LOC55833	IN_128		0.97	10.95	0.000
PPGB	NM_000308	protective protein for beta-galactosidase (galactosialidosis)	0.85	10.87	0.000
PCOLN3	NM_002768	procollagen (type III) N-endopeptidase	0.60	10.64	0.000
ARL5	NM_012097	ADP-ribosylation factor-like 5	0.65	11.31	0.000
CBARA1	NM_006077	calcium binding atopy-related autoantigen 1	0.70	9.55	0.000
LOC51720	IN_125		0.92	10.45	0.000
IGLL1	NM_020070	immunoglobulin lambda-like polypeptide 1	0.62	10.82	0.000
HPR	UP_32		0.64	10.88	0.000
RTN3	NM_006054	reticulon 3	0.70	10.70	0.000
KCNK5	NM_003740	potassium channel, subfamily K, member 5	0.83	10.54	0.000
GUK1	IN_54		0.65	10.60	0.000
FLJ10971	IN_120		0.55	10.61	0.000
NCOA1	IN_117		0.97	10.10	0.000
AKAP6	NM_004274	A kinase (PRKA) anchor protein 6	0.65	10.61	0.000
CTSF	NM_003793	cathepsin F	0.74	10.56	0.000
TNFRSF9	NM_001561	tumor necrosis factor receptor superfamily, member 9	0.61	10.70	0.000
F9	NM_000133	coagulation factor IX (plasma thromboplastic component, Christmas disease, hemophilia B)	0.57	10.28	0.000
NGB	NM_012341	GTP binding protein 4	0.64	10.62	0.000
RNF24	NM_007219	ring finger protein 24	1.03	10.15	0.000
RBMY1A1	NM_152585	RNA binding motif protein, Y-linked, family 1	0.77	10.82	0.000
PENK	NM_006211	proenkephalin	0.51	10.76	0.000
MGST1	NM_145764	microsomal glutathione S-transferase 1	0.62	10.73	0.000
ABCB6	NM_005689	ATP-binding cassette, sub-family B (MDR/TAP), member 6	0.75	10.88	0.000
CASP4	NM_001225	caspase 4, apoptosis-related cysteine protease	1.02	9.82	0.000
HSPA1B	NM_005346	heat shock 70kDa protein 1B	0.59	10.77	0.000
SOX13	NM_005686	SRY (sex determining region Y)-box 13	0.56	10.34	0.000
HOXB8	NM_024016	homeo box B8	0.72	10.52	0.000
PHF2	IN_0		1.00	9.78	0.000
POMC	NM_000939	proopiomelanocortin (adrenocorticotropin/ beta-lipotropin/ alpha-melanocyte stimulating hormone/ beta-melanocyte stimulating hormone/ beta-endorphin)	0.63	10.82	0.000
TDRKH	NM_006862	tudor and KH domain containing	1.04	8.38	0.000

LPXN	NM_004811	leupaxin	0.57	10.67	0.000
NR1I2	NM_003889	nuclear receptor subfamily 1, group I, member 2	0.57	10.34	0.000
KCNAB2	IN_57		0.67	10.78	0.000
CRYBA4	NM_001886	crystallin, beta A4	0.57	10.54	0.000
PFKFB4	NM_004567	6-phosphofructo-2-kinase/fructose-2,6-biphosphatase 4	0.57	10.41	0.000
PDE4C	NM_000923	phosphodiesterase 4C, cAMP-specific (phosphodiesterase E1 dunce homolog, Drosophila)	0.90	10.58	0.000
CHST6	IN_35		0.59	10.60	0.000
AMH	NM_000479	anti-Mullerian hormone	0.53	10.66	0.000
ABCF2	NM_005692	ATP-binding cassette, sub-family F (GCN20), member 2	0.61	10.82	0.000
HEXB	NM_000521	hexosaminidase B (beta polypeptide)	0.70	10.67	0.000
CNTN2	NM_005076	contactin 2 (axonal)	0.73	10.43	0.000
GNAL	NM_002071	guanine nucleotide binding protein (G protein), alpha activating activity polypeptide, olfactory type	0.65	11.09	0.000
RAB18	NM_021252	RAB18, member RAS oncogene family	0.65	10.87	0.000
WDR3	IN_57		0.56	9.09	0.000
C4B	NM_000592	complement component 4B	0.62	11.12	0.000
ILT10	NM_024317	leukocyte immunoglobulin-like receptor, subfamily A (without TM domain), member 5	0.80	10.65	0.000
TMG4	NM_024081	transmembrane gamma-carboxyglutamic acid protein 4	0.69	11.11	0.000
SLC22A3	NM_021977	solute carrier family 22 (extraneuronal monoamine transporter), member 3	0.97	9.54	0.000
NMBR	NM_002511	neuromedin B receptor	0.73	11.37	0.000
RBL1:500	NM_002895	retinoblastoma-like 1 (p107)	0.87	10.56	0.000
SUCLG1	NM_003849	succinate-CoA ligase, GDP-forming, alpha subunit	0.81	10.74	0.000
SMC1L1	NM_006306	SMC1 structural maintenance of chromosomes 1-like 1 (yeast)	0.53	10.69	0.000
MYF6	NM_002469	myogenic factor 6 (herculin)	0.56	10.91	0.000
RPC	NM_003729	RNA terminal phosphate cyclase domain 1	0.64	10.38	0.000
ATOX1	NM_004045	ATX1 antioxidant protein 1 homolog (yeast)	0.66	10.71	0.000
KRT7	NM_005556	keratin 7	0.62	10.72	0.000
SNTA1	NM_003098	syntrophin, alpha 1 (dystrophin-associated protein A1, 59kDa, acidic component)	0.85	10.05	0.000
MADH3	NM_005902	SMAD, mothers against DPP homolog 3 (Drosophila)	0.60	10.16	0.000
PDC	NM_002597	phosducin	1.03	8.11	0.000
TIMELESS	NM_003920	timeless homolog (Drosophila)	0.69	10.00	0.000
GLRA2	NM_002063	glycine receptor, alpha 2	0.64	10.85	0.000
TNFAIP6	NM_007115	tumor necrosis factor, alpha-induced protein 6	0.74	10.41	0.000
ARF4L	NM_001661	ADP-ribosylation factor 4-like	0.66	10.88	0.000
RUVBL2	NM_006666	RuvB-like 2 (E. coli)	0.98	8.67	0.000
KLK1	NM_002257	kallikrein 1, renal/pancreas/salivary	0.62	10.64	0.000
SERPINE1	NM_000602	serine (or cysteine) proteinase inhibitor, clade E (nexin, plasminogen activator inhibitor type 1), member 1	1.01	10.29	0.000
MST1	NTF_13		0.67	10.83	0.000
SERPIND1	NM_000185	serine (or cysteine) proteinase inhibitor, clade D (heparin cofactor), member 1	0.82	10.38	0.000
HNRPH2	IN_23		0.49	10.43	0.000
DFFA	NM_004401	DNA fragmentation factor, 45kDa, alpha polypeptide	0.65	10.69	0.000
RYR3	NM_001036	ryanodine receptor 3	0.76	10.78	0.000
HCN4	NM_005477	hyperpolarization activated cyclic nucleotide-gated potassium channel 4	0.57	10.89	0.000
GDF8	NM_005259	growth differentiation factor 8	0.67	10.53	0.000
CNOT2	UP_38		0.76	10.81	0.000
TYRO3	IN_126		0.64	10.43	0.000
LOC51278	NM_016545	immediate early response 5	0.71	10.19	0.000
PTTG2	NM_006607	pituitary tumor-transforming 2	0.56	10.68	0.000
SLC15A2	NM_021082	solute carrier family 15 (H+/peptide transporter), member 2	0.82	10.38	0.000
FOXI1	NM_012188	forkhead box I1	0.75	10.39	0.000
PIST	NM_020399	golgi associated PDZ and coiled-coil motif containing	0.62	10.49	0.000

ATP6F	NM_004047	ATPase, H+ transporting, lysosomal 21kDa, V0 subunit c''	0.63	10.70	0.000
HP	NM_005143	haptoglobin	0.70	10.51	0.000
TRRAP	NM_003496	transformation/transcription domain-associated protein	0.73	10.80	0.000
SYK	NM_003177	spleen tyrosine kinase	0.62	10.95	0.000
RAB3IL1	IN_46		0.52	10.83	0.000
RABGGTB	NM_004582	Rab geranylgeranyltransferase, beta subunit	0.57	10.84	0.000
NEB	IN_52		0.55	10.70	0.000
HOXB3	NM_002146	homeo box B3	0.61	10.68	0.000
IRF1	NM_002198	interferon regulatory factor 1	0.62	10.77	0.000
BPGM	NM_001724	2,3-bisphosphoglycerate mutase	0.64	10.57	0.000
CTNND2	IN_49		0.60	10.24	0.000
ITIH4	NM_002218	inter-alpha (globulin) inhibitor H4 (plasma Kallikrein-sensitive glycoprotein)	0.53	11.02	0.000
ALOX15	NM_001140	arachidonate 15-lipoxygenase	0.64	10.65	0.000
FGR	NM_005248	Gardner-Rasheed feline sarcoma viral (v-fgr) oncogene homolog	0.56	10.51	0.001
FGF5	NM_004464	fibroblast growth factor 5	0.53	10.55	0.001
TIP30	NM_006410	HIV-1 Tat interactive protein 2, 30kDa	1.13	9.02	0.001
RARRES3	NM_004585	retinoic acid receptor responder (tazarotene induced) 3	0.64	10.54	0.001
T1A-2	IN_118		1.08	8.84	0.001
MOG	NM_002433	myelin oligodendrocyte glycoprotein	0.68	10.45	0.001
LIM	NM_006457	LIM protein (similar to rat protein kinase C-binding enigma)	0.54	10.81	0.001
NLVCF	NM_003776	mitochondrial ribosomal protein L40	0.78	10.09	0.001
SLAP	NM_007159	sarcolemma associated protein	0.62	10.68	0.001
MIF	NM_002415	macrophage migration inhibitory factor (glycosylation-inhibiting factor)	1.64	8.45	0.001
TRIP4	NM_016213	thyroid hormone receptor interactor 4	0.54	11.19	0.001
EEF1B2	NM_021121	eukaryotic translation elongation factor 1 beta 2	0.91	10.58	0.001
SLC6A5	NM_004211	solute carrier family 6 (neurotransmitter transporter, glycine), member 5	0.58	10.17	0.001
CRABP1	NM_004378	cellular retinoic acid binding protein 1	0.69	10.33	0.001
ZNF254	IN_77		0.79	10.65	0.001
FPGS	NM_004957	folylpolyglutamate synthase	0.50	10.78	0.001
BET3	NM_014408	trafficking protein particle complex 3	0.72	10.80	0.001
NMT1	IN_20		0.51	10.84	0.001
RALB	IN_22		0.51	10.26	0.001
CNOT8	NM_004779	CCR4-NOT transcription complex, subunit 8	1.14	11.42	0.001
GP5	NM_004488	glycoprotein V (platelet)	0.67	10.87	0.001
NKTR	NM_005385	natural killer-tumor recognition sequence	0.89	10.59	0.001
CHM-I	NM_007015	leukocyte cell derived chemotaxin 1	0.56	10.17	0.001
RAP1GDS1	IN_44		0.56	10.53	0.001
RAB3A	NM_002866	RAB3A, member RAS oncogene family	0.63	10.62	0.001
NFRKB	UP_40		0.59	10.52	0.001
ZNF236	NM_007345	zinc finger protein 236	0.59	10.62	0.001
KNSL3	IN_44		0.61	10.80	0.001
RARB	NM_000965	retinoic acid receptor, beta	0.57	10.71	0.001
SHC3	NM_016848	src homology 2 domain containing transforming protein C3	0.62	10.82	0.001
GATA2	IN_15		0.58	10.78	0.001
NUP155	NM_153485	nucleoporin 155kDa	0.62	10.51	0.001
MOCS2	NM_176806	molybdenum cofactor synthesis 2	0.55	10.64	0.001
LYPLA1	UP_81		0.58	10.47	0.001
KRT5	NM_000424	keratin 5 (epidermolysis bullosa simplex, Dowling-Meara/Kobner/Weber-Cockayne types)	0.77	10.74	0.001
DMAP1	NM_019100	DNA methyltransferase 1 associated protein 1	0.70	11.08	0.001
MAGEA2	NM_153488	melanoma antigen, family A, 2B	0.68	10.99	0.001

HYAL2	NM_003773	hyaluronoglucosaminidase 2	1.19	9.97	0.001
SDHB	NM_003000	succinate dehydrogenase complex, subunit B, iron sulfur (Ip)	0.63	10.62	0.001
NFKBIL1	NM_005007	nuclear factor of kappa light polypeptide gene enhancer in B-cells inhibitor-like 1	1.01	10.62	0.001
RPL36	NM_033643	ribosomal protein L36	0.73	10.19	0.001
S100A4	NM_002961	S100 calcium binding protein A4 (calcium protein, calvasculin, metastasin, murine placental homolog)	0.50	11.10	0.001
PAX8	NM_003466	paired box gene 8	0.68	10.47	0.001
LCAT	NM_000229	lecithin-cholesterol acyltransferase	0.70	10.69	0.001
GNG3	NM_012202	guanine nucleotide binding protein (G protein), gamma 3	0.55	10.77	0.001
P12	NM_019896	polymerase (DNA-directed), epsilon 4 (p12 subunit)	1.14	9.98	0.001
PNR	NM_003967	putative neurotransmitter receptor	0.84	10.66	0.001
MYCN	NM_005378	v-myc myelocytomatosis viral related oncogene, neuroblastoma derived (avian)	1.03	10.84	0.001
ITGB3BP	NM_014288	integrin beta 3 binding protein (beta3-endonexin)	0.59	10.78	0.001
HNRPL	UP_26		1.07	9.86	0.001
CTSC	NM_001814	cathepsin C	0.70	11.31	0.001
CSE1L	NM_001316	CSE1 chromosome segregation 1-like (yeast)	0.88	10.84	0.001
PCSK1	NM_000439	proprotein convertase subtilisin/kexin type 1	1.07	9.48	0.001
PPBP	NM_002704	pro-platelet basic protein (chemokine (C-X-C motif) ligand 7)	0.50	10.62	0.001
H2BFS	NM_017445	H2B histone family, member S	0.57	10.66	0.001
CIRBP	NM_001280	cold inducible RNA binding protein	1.27	10.37	0.001
ATP2B3	NM_021949	ATPase, Ca++ transporting, plasma membrane 3	0.57	10.85	0.001
IVL	NM_005547	involucrin	0.52	10.97	0.001
VAMP8	NM_003761	vesicle-associated membrane protein 8 (endobrevin)	0.88	10.29	0.001
PTGES	NM_004878	prostaglandin E synthase	0.61	10.81	0.001
CSF2	NM_000758	colony stimulating factor 2 (granulocyte-macrophage)	0.68	10.56	0.001
TUBB	NM_178012	tubulin, beta polypeptide paralog	0.67	10.47	0.001
C4BPA	NM_000715	complement component 4 binding protein, alpha	0.56	10.50	0.001
MAPK7	NM_139033	mitogen-activated protein kinase 7	0.76	10.68	0.001
CACNA1B	IN_155		0.67	10.50	0.001
CALCYON	IN_105		0.68	10.24	0.001
PCDHB6	NM_018939	protocadherin beta 6	0.58	10.50	0.001
RQCD1	NM_005444	RCD1 required for cell differentiation1 homolog (S. pombe)	0.74	10.45	0.001
TOPK	NM_018492	T-LAK cell-originated protein kinase	0.45	10.89	0.001
GUCY1B3	NM_000857	guanylate cyclase 1, soluble, beta 3	0.59	10.45	0.001
PHLDA3	NM_012396	pleckstrin homology-like domain, family A, member 3	0.86	11.04	0.001
TMPRSS3	NM_024022	transmembrane protease, serine 3	0.52	11.03	0.001
SPAG6	NM_012443	sperm associated antigen 6	0.61	10.69	0.001
ARPC1A	IN_119		0.48	11.06	0.001
TRA1	NM_003299	tumor rejection antigen (gp96) 1	1.11	10.37	0.001
MAPK8IP1	NM_005456	mitogen-activated protein kinase 8 interacting protein 1	0.88	9.68	0.001
VNN2	NM_004665	vanin 2	0.52	10.43	0.001
SYT1	NM_005639	synaptotagmin I	0.55	10.79	0.001
SLC1A3	NM_004172	solute carrier family 1 (glial high affinity glutamate transporter), member 3	0.49	10.88	0.001
INPPL1	IN_20		0.50	10.89	0.001
PHF1	NM_024165	PHD finger protein 1	0.57	10.74	0.001
CPZ	NM_003652	carboxypeptidase Z	0.65	10.88	0.001
ING1	NM_005537	inhibitor of growth family, member 1	0.53	10.89	0.001
SFRS2	NM_003016	splicing factor, arginine/serine-rich 2	0.67	10.49	0.001
COL17A1	NM_000494	collagen, type XVII, alpha 1	0.49	10.49	0.001
ELK4	IN_170		0.67	10.51	0.001
VPS33B	NM_018668	vacuolar protein sorting 33B (yeast)	0.49	10.67	0.001

CSHL1	NM_001318	chorionic somatomammotropin hormone-like 1	0.54	10.73	0.001
H6PD	NM_004285	hexose-6-phosphate dehydrogenase (glucose 1-dehydrogenase)	0.69	9.85	0.001
USP14	NTF_90		0.62	11.12	0.001
EIF3S7	NM_003753	eukaryotic translation initiation factor 3, subunit 7 zeta, 66/67kDa	0.65	10.22	0.001
SMC4L1	NM_005496	SMC4 structural maintenance of chromosomes 4-like 1 (yeast)	0.57	10.28	0.001
PCDH11	NM_032968	protocadherin 11 X-linked	0.59	11.04	0.001
KCND2	IN_16		0.53	11.01	0.001
HCNGP	NM_013260	transcriptional regulator protein	0.84	10.34	0.001
STAT4	IN_179		0.63	10.63	0.001
PSMD8	NM_002812	proteasome (prosome, macropain) 26S subunit, non-ATPase, 8	0.52	10.59	0.001
SEC24D	NM_014822	SEC24 related gene family, member D (S. cerevisiae)	0.56	10.40	0.001
ENC1	NM_003633	ectodermal-neural cortex (with BTB-like domain)	0.64	10.71	0.001
PCDH8	NM_002590	protocadherin 8	0.54	10.39	0.001
FCAR	NM_133269	Fc fragment of IgA, receptor for	0.55	10.32	0.001
CHEK1	NM_001274	CHK1 checkpoint homolog (S. pombe)	1.02	10.28	0.001
SIRP-BETA-1	NM_006065	signal-regulatory protein beta 1	0.53	10.35	0.001
SGCD	IN_6		0.56	11.12	0.001
ADK	NM_001123	adenosine kinase	0.70	10.99	0.001
RBT1	NM_013368	SERTA domain containing 3	0.70	10.82	0.001
MDH1	NM_005917	malate dehydrogenase 1, NAD (soluble)	0.55	10.62	0.001
CYP2D6	NM_000106	cytochrome P450, family 2, subfamily D, polypeptide 6	0.59	10.32	0.001
SLC12A1	NM_000338	solute carrier family 12 (sodium/potassium/chloride transporters), member 1	0.61	9.70	0.001
FBXO24	NM_012172	F-box protein 24	0.61	10.81	0.001
LOR	NM_000427	loricrin	0.51	10.73	0.001
PDE4D	IN_0		0.72	10.13	0.001
GCN5L1	NM_001487	biogenesis of lysosome-related organelles complex-1, subunit 1	0.58	10.40	0.001
SSX1	IN_22		0.51	10.83	0.001
LAF4	IN_53		0.64	10.59	0.001
TEP1	IN_100		0.53	10.69	0.001
HF1	NM_000186	H factor 1 (complement)	0.86	10.45	0.001
LMO7	IN_44		0.45	10.66	0.001
HBQ1	NM_005331	hemoglobin, theta 1	1.22	10.87	0.001
PFDN1	NTF_2		0.64	10.44	0.001
RPP14	NTF_113		0.81	9.97	0.001
PRKCL2	NM_006256	protein kinase C-like 2	0.61	11.18	0.001
DDX10	NM_004398	DEAD (Asp-Glu-Ala-Asp) box polypeptide 10	0.78	10.51	0.001
NAPG	NM_003826	N-ethylmaleimide-sensitive factor attachment protein, gamma	0.59	10.56	0.001
PTP4A1	NM_003463	protein tyrosine phosphatase type IVA, member 1	0.59	10.48	0.001
SYCP2	NM_014258	synaptonemal complex protein 2	0.91	9.52	0.001
ITGB8	NM_002214	integrin, beta 8	0.56	11.16	0.001
FACVL1	NM_003645	solute carrier family 27 (fatty acid transporter), member 2	0.56	10.51	0.001
SPAG11	NM_016512	sperm associated antigen 11	0.97	8.99	0.001
MC5R	NM_005913	melanocortin 5 receptor	0.60	10.84	0.001
TCAP	NM_003673	titin-cap (telethonin)	0.60	10.95	0.001
H2AFO	NM_003517	histone 2, H2ac	0.47	10.62	0.001
C6ORF9	NM_022107	G-protein signalling modulator 3 (AGS3-like, C. elegans)	0.61	10.64	0.001
CLCA2	NM_006536	chloride channel, calcium activated, family member 2	0.56	10.77	0.001
PNOC	NM_006228	prepronociceptin	0.54	10.43	0.001
TCEB1L	NM_006930	S-phase kinase-associated protein 1A (p19A)	0.60	10.41	0.001
PSMD1	NM_002807	proteasome (prosome, macropain) 26S subunit, non-ATPase, 1	0.75	10.21	0.001

DLST	NM_001933	dihydrolipoamide S-succinyltransferase (E2 component of 2-oxo-glutarate complex)	0.74	10.15	0.001
LDB2	IN_54		0.69	10.87	0.001
KCNH4	NM_012285	potassium voltage-gated channel, subfamily H (eag-related), member 4	0.52	10.29	0.001
RPGRIP1	IN_138		0.83	9.79	0.001
NDUFB8	NM_005004	NADH dehydrogenase (ubiquinone) 1 beta subcomplex, 8, 19kDa	0.50	10.73	0.001
IL1RL1	NM_003856	interleukin 1 receptor-like 1	0.63	9.75	0.001
TCF4	NM_003199	transcription factor 4	0.57	10.55	0.001
CEACAM8	NM_001816	carcinoembryonic antigen-related cell adhesion molecule 8	0.69	10.96	0.001
UK114	NM_005836	heat-responsive protein 12	1.19	10.27	0.001
GOLGA4:649	UP_48		0.58	10.74	0.001
STXBP1	NM_003165	syntaxin binding protein 1	0.59	10.34	0.001
AMPD3	NM_000480	adenosine monophosphate deaminase (isoform E)	1.01	10.28	0.001
SMUG1	NM_014311	single-strand selective monofunctional uracil DNA glycosylase	0.52	10.68	0.001
MTM1	NM_000252	myotubular myopathy 1	0.67	9.37	0.001
PGLYRP	NM_005091	peptidoglycan recognition protein 1	0.90	11.17	0.001
TEAD1	IN_146		0.65	10.18	0.001
BRD1	NM_014577	bromodomain containing 1	0.44	10.60	0.001
CDC25A:500	NM_001789	cell division cycle 25A	0.73	10.53	0.001
C6	IN_22		0.57	10.36	0.001
INPP4B	NM_003866	inositol polyphosphate-4-phosphatase, type II, 105kDa	0.59	10.25	0.001
TGFBR3	NM_003243	transforming growth factor, beta receptor III (betaglycan, 300kDa)	0.44	10.81	0.001
DGKQ	IN_46		0.46	10.75	0.001
TCP10	UP_139		0.64	10.13	0.001
LIPC	NM_000236	lipase, hepatic	0.47	10.95	0.001
FUT1	NM_000148	fucosyltransferase 1 (galactoside 2-alpha-L-fucosyltransferase)	0.58	10.56	0.001
CYP3A4	NM_017460	cytochrome P450, family 3, subfamily A, polypeptide 4	0.69	10.57	0.001
TIMP2	IN_148		0.58	10.46	0.001
CPNE6	NM_006032	copine VI (neuronal)	0.66	10.63	0.001
PCDH9	NM_020403	protocadherin 9	0.70	10.22	0.001
SPAG8	NM_012436	sperm associated antigen 8	0.69	11.17	0.001
EIF2S1	NM_004094	eukaryotic translation initiation factor 2, subunit 1 alpha, 35kDa	0.50	10.65	0.001
DBY	NM_004660	DEAD (Asp-Glu-Ala-Asp) box polypeptide 3, Y-linked	0.82	9.83	0.001
RAD51L3	NM_002878	RAD51-like 3 (S. cerevisiae)	1.05	9.82	0.001
MSX1	NM_002448	msh homeo box homolog 1 (Drosophila)	0.54	10.68	0.001
KCNG1	NM_002237	potassium voltage-gated channel, subfamily G, member 1	0.46	10.92	0.001
MMP28	NM_024302	matrix metalloproteinase 28	0.65	10.77	0.001
CCND2	NM_001759	cyclin D2	0.63	10.79	0.001
MCAM	IN_158		0.73	10.83	0.001
JAM1	NM_144503	F11 receptor	0.66	10.51	0.001
GFAP	NM_002055	glial fibrillary acidic protein	0.52	10.49	0.001
DDX1	NM_004939	DEAD (Asp-Glu-Ala-Asp) box polypeptide 1	0.57	10.57	0.001
SP140	NM_007237	SP140 nuclear body protein	0.59	10.53	0.001
CGB	NM_033142	chorionic gonadotropin, beta polypeptide 7	0.87	10.59	0.001
NUDT1	IN_142		0.52	10.48	0.001
PROC	NM_000312	protein C (inactivator of coagulation factors Va and VIIIa)	0.58	10.94	0.001
MOS	NM_005372	v-mos Moloney murine sarcoma viral oncogene homolog	0.53	10.98	0.001
CTF1	NM_001330	cardiotrophin 1	0.67	10.70	0.001
S100A5	UP_7		0.67	10.97	0.001
EFNA5	NM_001962	ephrin-A5	0.65	10.63	0.001
SFRS11	NM_004768	splicing factor, arginine/serine-rich 11	0.56	10.43	0.001

CLDN2	NM_020384	claudin 2	0.61	10.30	0.001
MLN64	NM_006804	START domain containing 3	0.55	10.35	0.001
FRAG1	NTF_119		0.70	10.98	0.001
MASA	NM_021204	E-1 enzyme	0.56	10.75	0.001
CHD1	NM_001270	chromodomain helicase DNA binding protein 1	0.65	11.02	0.001
EDNRA	NM_001957	endothelin receptor type A	0.73	10.49	0.001
GSTA4	NM_001512	glutathione S-transferase A4	0.53	10.85	0.001
TAF2I	NM_005643	TAF11 RNA polymerase II, TATA box binding protein (TBP)-associated factor, 28kDa	1.29	9.62	0.001
CLDN8	NM_012132		0.61	10.29	0.001
HRAS	NM_176795	v-Ha-ras Harvey rat sarcoma viral oncogene homolog	0.48	10.77	0.002
PIK3CG	NM_002649	phosphoinositide-3-kinase, catalytic, gamma polypeptide	0.47	10.41	0.002
ELL	NM_006532	elongation factor RNA polymerase II	0.53	10.59	0.002
BRCA2	NM_000059	breast cancer 2, early onset	0.56	10.70	0.002
SLC5A1	NM_000343	solute carrier family 5 (sodium/glucose cotransporter), member 1	0.56	11.14	0.002
SERF2	NTF_95		0.56	10.74	0.002
ITGB4BP	NM_002212	integrin beta 4 binding protein	0.50	10.51	0.002
TBX1	NM_005992	T-box 1	0.45	10.80	0.002
H4FI	NM_003544	histone 1, H4b	0.54	9.49	0.002
XYLB	NM_005108	xylulokinase homolog (H. influenzae)	0.53	11.26	0.002
MLH1	NM_000249	mutL homolog 1, colon cancer, nonpolyposis type 2 (E. coli)	0.62	9.99	0.002
ITGA2	NM_002203	integrin, alpha 2 (CD49B, alpha 2 subunit of VLA-2 receptor)	0.65	10.81	0.002
PRB3	NM_006249	proline-rich protein BstNI subfamily 3	0.54	10.37	0.002
MGP	NM_000900	matrix Gla protein	0.51	11.00	0.002
LOC51673	NM_016140	brain specific protein	0.53	10.80	0.002
PPM1G	NM_177983	protein phosphatase 1G (formerly 2C), magnesium-dependent, gamma isoform	0.61	10.93	0.002
UNC119	NM_005148	unc-119 homolog (C. elegans)	0.57	10.91	0.002
DGAT	NM_012079	diacylglycerol O-acyltransferase homolog 1 (mouse)	1.03	10.12	0.002
SEC4L	NM_006822	RAB40B, member RAS oncogene family	0.69	9.83	0.002
POU4F2	NM_004575	POU domain, class 4, transcription factor 2	0.48	10.32	0.002
DPM1	NM_003859	dolichyl-phosphate mannosyltransferase polypeptide 1, catalytic subunit	0.60	10.48	0.002
PPP2R5C	NM_015512		0.57	10.59	0.002
F10	NM_000504	coagulation factor X	0.52	10.50	0.002
GNA14	NM_004297	guanine nucleotide binding protein (G protein), alpha 14	0.48	10.88	0.002
TLR4	NM_003266	toll-like receptor 4	0.49	11.00	0.002
MGST3	NM_004528	microsomal glutathione S-transferase 3	0.75	10.57	0.002
KHSRP	NM_003685	KH-type splicing regulatory protein (FUSE binding protein 2)	0.59	10.74	0.002
LGALS1	NM_002305	lectin, galactoside-binding, soluble, 1 (galectin 1)	0.55	10.55	0.002
LOC56242	NM_021047	zinc finger protein 253	0.46	11.04	0.002
TTK	NM_003318	TTK protein kinase	0.46	10.61	0.002
ABCC5	NM_005688	ATP-binding cassette, sub-family C (CFTR/MRP), member 5	0.50	11.00	0.002
MBL2	NM_000242	mannose-binding lectin (protein C) 2, soluble (opsonic defect)	0.60	10.80	0.002
IFNW1	NM_002177	interferon, omega 1	0.56	10.94	0.002
NUMB	IN_132		0.68	9.90	0.002
ICSBP1	NM_002163	interferon consensus sequence binding protein 1	0.54	10.28	0.002
L1CAM	NM_000425	L1 cell adhesion molecule (hydrocephalus, stenosis of aqueduct of Sylvius 1, MASA (mental retardation, aphasia, shuffling gait and adducted thumbs) syndrome, spastic parap	0.60	10.44	0.002
TPT	NM_014317	trans-prenyltransferase	0.46	10.81	0.002
KCND3	IN_20		0.50	10.73	0.002
MSH3	NM_002439	mutS homolog 3 (E. coli)	0.55	10.89	0.002
LOC58511	NM_021233	DNase II-like acid DNase	0.63	9.87	0.002
FSHR	NM_000145	follicle stimulating hormone receptor	0.49	10.44	0.002

WASF1	IN_88		0.95	9.60	0.002
NUP153	NM_005124	nucleoporin 153kDa	0.55	10.44	0.002
HCRT	NM_001524	hypocretin (orexin) neuropeptide precursor	0.67	10.67	0.002
CNR1	NM_001840	cannabinoid receptor 1 (brain)	1.84	6.48	0.002
GPHN	NM_020806	gephyrin	0.56	10.97	0.002
ELL2	UP_112		0.84	10.06	0.002
HCRTR1	NM_001525	hypocretin (orexin) receptor 1	0.73	10.64	0.002
AP1S2	IN_108		0.57	9.94	0.002
DRD3	IN_53		0.52	10.97	0.002
TAZ	UP_171		0.90	9.93	0.002
MPZL1	NM_003953	myelin protein zero-like 1	0.61	10.21	0.002
SEMA3B	NM_004636	sema domain, immunoglobulin domain (Ig), short basic domain, secreted, (semaphorin) 3B	0.56	10.53	0.002
BCL2L10	NM_020396	BCL2-like 10 (apoptosis facilitator)	0.47	10.88	0.002
KIR2DS2	NM_012312	killer cell immunoglobulin-like receptor, two domains, short cytoplasmic tail, 2	0.51	10.94	0.002
CEP2	NM_006779	CDC42 effector protein (Rho GTPase binding) 2	0.71	9.81	0.002
CTRL	NM_001907	chymotrypsin-like	0.68	10.47	0.002
LOC55907	NM_018686	cytidine monophosphate N-acetylneuraminic acid synthetase	0.65	10.76	0.002
SRPK1	NM_003137	SFRS protein kinase 1	0.70	10.31	0.002
MAP3K5	NM_005923	mitogen-activated protein kinase kinase kinase 5	0.46	10.73	0.002
SSTR3	NM_001051	somatostatin receptor 3	0.55	10.74	0.002
RABAC1	NM_006423	Rab acceptor 1 (prenylated)	1.11	8.57	0.002
DBCCR1	NM_014618	deleted in bladder cancer 1	0.48	10.84	0.002
LLGL1	NM_004140	lethal giant larvae homolog 1 (Drosophila)	0.47	10.39	0.002
SRM300	NM_016333	serine/arginine repetitive matrix 2	0.65	10.84	0.002
DNAJB1	NM_006145	DnaJ (Hsp40) homolog, subfamily B, member 1	0.46	10.43	0.002
AVIL	NM_006576	advillin	0.82	9.43	0.002
CLK1	NM_004071	CDC-like kinase 1	0.73	10.31	0.002
HS3ST3A1	NM_006042	heparan sulfate (glucosamine) 3-O-sulfotransferase 3A1	0.50	10.87	0.002
CNN1	NM_001299	calponin 1, basic, smooth muscle	2.08	6.75	0.002
HNRPC	UP_11		0.52	10.58	0.002
INDO	NM_002164	indoleamine-pyrrole 2,3 dioxygenase	0.59	10.29	0.002
MC3R	NM_019888	melanocortin 3 receptor	0.45	10.62	0.002
ZFPL1	NM_006782	zinc finger protein-like 1	0.55	11.14	0.002
FCGBP	NM_003890	Fc fragment of IgG binding protein	0.70	10.04	0.002
HHLA1	NM_005712	HERV-H LTR-associating 1	0.55	10.47	0.002
G1P3	NM_002038	interferon, alpha-inducible protein (clone IFI-6-16)	0.65	10.94	0.002
IL4R	NM_000418	interleukin 4 receptor	0.45	10.78	0.002
PRSC1	NM_005606	legumain	0.54	11.03	0.002
LRPAP1	NM_002337	low density lipoprotein receptor-related protein associated protein 1	1.37	10.13	0.002
P125	NM_007190	SEC23 interacting protein	0.69	10.18	0.002
NEF3	NM_005382	neurofilament 3 (150kDa medium)	0.44	10.66	0.002
RB1	NM_000321	retinoblastoma 1 (including osteosarcoma)	0.45	10.84	0.002
NME3	NM_002513	non-metastatic cells 3, protein expressed in	0.64	10.60	0.002
H2AFL	NM_003512	histone 1, H2ac	0.51	10.56	0.002
DF	NM_001928	D component of complement (adipsin)	0.47	10.80	0.002
GNS	NM_002076	glucosamine (N-acetyl)-6-sulfatase (Sanfilippo disease IIID)	0.48	10.68	0.002
GRM1	NM_000838	glutamate receptor, metabotropic 1	0.46	10.82	0.002
CMRF35	NM_006678	CMRF35 leukocyte immunoglobulin-like receptor	0.75	9.61	0.002
CLDN16	NM_006580	claudin 16	0.72	9.86	0.002
CILP	NM_003613	cartilage intermediate layer protein, nucleotide pyrophosphohydrolase	0.61	10.61	0.002

GCH1	NM_000161	GTP cyclohydrolase 1 (dopa-responsive dystonia)	0.43	10.87	0.002
STX7	NM_003569	syntaxin 7	0.52	10.93	0.002
CPNE3	IN_79		0.71	10.01	0.002
SMARCA1	IN_51		0.44	10.48	0.002
UBE2G2	NM_003343	ubiquitin-conjugating enzyme E2G 2 (UBC7 homolog, yeast)	0.67	10.39	0.002
SNRPG	NM_003096	small nuclear ribonucleoprotein polypeptide G	0.58	10.42	0.003
RDS	NM_000322	retinal degeneration, slow	0.41	10.87	0.003
NNT	IN_142		0.59	9.40	0.003
RPS5	NM_001009	ribosomal protein S5	0.51	10.74	0.003
KCNA6	NM_002235	potassium voltage-gated channel, shaker-related subfamily, member 6	0.41	10.70	0.003
DEPP	NM_007021	chromosome 10 open reading frame 10	0.59	9.90	0.003
CDC6:500	NM_001254	CDC6 cell division cycle 6 homolog (S. cerevisiae)	0.67	10.76	0.003
CSDA	NM_003651	cold shock domain protein A	0.69	9.82	0.003
SERPINF2	NM_000934	serine (or cysteine) proteinase inhibitor, clade F (alpha-2 antiplasmin, pigment epithelium derived factor), member 2	0.52	10.70	0.003
GGT1	UP_20		0.49	10.65	0.003
ZNF211	NM_006385	zinc finger protein 211	0.72	9.85	0.003
POU5F1	NM_002701	POU domain, class 5, transcription factor 1	0.51	10.49	0.003
KNG	NM_000893	kininogen 1	0.62	10.80	0.003
H4FB	NM_003539	histone 1, H4d	0.42	10.72	0.003
HAS2	IN_141		0.57	10.27	0.003
EGF	NM_001963	epidermal growth factor (beta-urogastrone)	0.70	10.73	0.003
LAMC1	IN_20		0.73	10.57	0.003
MATN2	NM_002380	matrilin 2	0.43	10.79	0.003
SAE1	NM_005500	SUMO-1 activating enzyme subunit 1	0.47	10.76	0.003
EEF1D	UP_22		0.42	10.57	0.003
STK16	NM_003691	serine/threonine kinase 16	0.54	10.45	0.003
SEC14L2	NM_012429	SEC14-like 2 (S. cerevisiae)	0.72	10.37	0.003
MSRA	IN_7		0.45	10.80	0.003
TMSB4Y	NM_004202	thymosin, beta 4, Y-linked	0.68	10.11	0.003
G3BP	NM_005754	Ras-GTPase-activating protein SH3-domain-binding protein	0.50	10.69	0.003
HSD3B1	NM_000862	hydroxy-delta-5-steroid dehydrogenase, 3 beta- and steroid delta-isomerase 1	0.43	10.72	0.003
LOC51189	NM_016311	ATPase inhibitory factor 1	0.43	10.60	0.003
ANKRD3	NM_020639	ankyrin repeat domain 3	0.50	10.84	0.003
PSME1	NM_006263	proteasome (prosome, macropain) activator subunit 1 (PA28 alpha)	0.59	10.68	0.003
KIF4A	NM_012310	kinesin family member 4A	0.60	10.70	0.003
LHCGR	IN_53		0.54	10.65	0.003
ABCF1	NM_001090	ATP-binding cassette, sub-family F (GCN20), member 1	0.51	10.95	0.003
RAD9	NM_004584	RAD9 homolog A (S. pombe)	0.67	9.29	0.003
KIAA0751	NM_014677	regulating synaptic membrane exocytosis 2	0.42	10.90	0.003
RAGE	NM_014226	renal tumor antigen	0.49	10.60	0.003
RGS2	NM_002923	regulator of G-protein signalling 2, 24kDa	0.67	10.63	0.003
NEU2	NM_005383	sialidase 2 (cytosolic sialidase)	0.47	10.66	0.003
KPNB1	NM_002265	karyopherin (importin) beta 1	0.54	10.90	0.003
PF4	NM_002619	platelet factor 4 (chemokine (C-X-C motif) ligand 4)	0.94	9.29	0.003
ADRA1D	NM_000678	adrenergic, alpha-1D-, receptor	0.65	10.58	0.003
LOC51668	NM_016126	chromosome 1 open reading frame 41	0.61	10.96	0.003
HUM2DD	NM_014475	dihydrodiol dehydrogenase (dimeric)	0.46	10.56	0.003
SUPT6H	IN_143		0.49	10.83	0.003
GALT	NM_000155	galactose-1-phosphate uridylyltransferase	0.43	10.35	0.003
HSPC141	NM_014172	phosphohistidine phosphatase 1	0.81	9.82	0.003

PLAGL1	IN_35		0.39	11.07	0.003
PSMD5	NM_005047	proteasome (prosome, macropain) 26S subunit, non-ATPase, 5	0.39	10.51	0.003
ECHS1	IN_53		0.49	10.46	0.003
SC65	NM_006455	synaptonemal complex protein SC65	0.64	10.16	0.003
SIX4	NM_017420	sine oculis homeobox homolog 4 (Drosophila)	0.41	10.75	0.003
IL12A	NM_000882	interleukin 12A (natural killer cell stimulatory factor 1, cytotoxic lymphocyte maturation factor 1, p35)	0.47	10.74	0.003
NCK1	IN_37		0.59	10.17	0.003
CSPG6	NM_005445	chondroitin sulfate proteoglycan 6 (bamacan)	0.58	10.37	0.003
ARHGEF2	NM_004723	rho/rac guanine nucleotide exchange factor (GEF) 2	0.58	11.06	0.003
IL1RAP	NM_002182	interleukin 1 receptor accessory protein	0.66	11.24	0.003
CSTF3	NM_001326	cleavage stimulation factor, 3' pre-RNA, subunit 3, 77kDa	0.61	10.38	0.003
PPIF	NM_005729	peptidylprolyl isomerase F (cyclophilin F)	0.65	10.42	0.003
ZNF267	NM_003414	zinc finger protein 267	0.46	10.93	0.003
CHL1	NM_006614	cell adhesion molecule with homology to L1CAM (close homolog of L1)	0.67	9.91	0.003
SLC9A6	NM_006359	solute carrier family 9 (sodium/hydrogen exchanger), isoform 6	0.58	9.27	0.003
HMBS	NM_000190	hydroxymethylbilane synthase	0.87	10.54	0.003
COMP	NM_000095	cartilage oligomeric matrix protein	0.40	10.36	0.003
RNAC	NM_005772	RNA terminal phosphate cyclase-like 1	0.51	10.46	0.003
SRM160	NM_005839	serine/arginine repetitive matrix 1	0.65	9.85	0.003
DDEF2	IN_139		0.47	10.52	0.003
REM	NM_014012	RAS (RAD and GEM)-like GTP-binding 1	0.44	10.58	0.003
KLRC1	NM_002259	killer cell lectin-like receptor subfamily C, member 1	0.47	11.06	0.003
SWAP2	NM_007056	splicing factor, arginine/serine-rich 16 (suppressor-of-white-apricot homolog, Drosophila)	0.99	8.47	0.003
KLRB1	NM_002258	killer cell lectin-like receptor subfamily B, member 1	0.50	10.62	0.003
UBTF	NM_014233	upstream binding transcription factor, RNA polymerase I	0.60	10.51	0.003
LECT2	NM_002302	leukocyte cell-derived chemotaxin 2	0.55	10.40	0.003
TFEC	IN_87		0.51	10.13	0.003
INPP1	NM_002194	inositol polyphosphate-1-phosphatase	0.44	11.07	0.003
PTH	NM_000315	parathyroid hormone	0.75	10.02	0.003
TOP1	NM_003286	topoisomerase (DNA) I	0.38	10.84	0.003
HNRPM	IN_19		0.50	10.35	0.004
TAB1	NM_006116	mitogen-activated protein kinase kinase kinase 7 interacting protein 1	0.94	8.13	0.004
ARPC3	NM_005719	actin related protein 2/3 complex, subunit 3, 21kDa	0.42	10.89	0.004
EPX	NM_000502	eosinophil peroxidase	0.54	10.67	0.004
OCIA	NM_017830	ovarian carcinoma immunoreactive antigen	0.54	10.94	0.004
PDE2A	NM_002599	phosphodiesterase 2A, cGMP-stimulated	0.49	10.51	0.004
POLR2E	NM_002695	polymerase (RNA) II (DNA directed) polypeptide E, 25kDa	0.43	10.70	0.004
NCBP1	NM_002486	nuclear cap binding protein subunit 1, 80kDa	0.51	10.75	0.004
TRAF2	NM_021138	TNF receptor-associated factor 2	0.99	9.35	0.004
NOS1	NM_000620	nitric oxide synthase 1 (neuronal)	0.54	10.72	0.004
RNF9	NM_052828	tripartite motif-containing 10	0.44	10.74	0.004
MB	NM_005368	myoglobin	0.49	10.63	0.004
ATP8B1	NM_005603	ATPase, Class I, type 8B, member 1	0.51	10.61	0.004
CRYBB3	NM_004076	crystallin, beta B3	0.49	10.33	0.004
FOXH1	NM_003923	forkhead box H1	0.57	10.44	0.004
13CDNA73	NM_023037	hypothetical protein CG003	0.40	10.75	0.004
MAD	NM_002357	MAX dimerization protein 1	0.60	10.55	0.004
RABEX5	NM_014504	RAB guanine nucleotide exchange factor (GEF) 1	0.46	10.32	0.004
HXB	NM_002160	tenascin C (hexabrachion)	0.60	9.79	0.004
TCEAL1	NM_004780	transcription elongation factor A (SII)-like 1	0.60	10.38	0.004

TMEM5	NM_014254	transmembrane protein 5	0.43	10.76	0.004
PCOLCE	NM_002593	procollagen C-endopeptidase enhancer	0.37	10.69	0.004
SLC4A8	NM_004858	solute carrier family 4, sodium bicarbonate cotransporter, member 8	0.90	9.86	0.004
IKBKG	IN_58		0.40	10.68	0.004
FLJ11126	NM_018332	hypothetical protein FLJ11126	0.68	10.42	0.004
EEF1A1L14	NTF_20		0.50	10.75	0.004
SSI-1	NM_003745	suppressor of cytokine signaling 1	0.88	9.98	0.004
PGAM1	NM_002629	phosphoglycerate mutase 1 (brain)	0.50	10.89	0.004
HOXB5	NM_002147	homeo box B5	1.07	10.78	0.004
PCM1	NM_006197	pericentriolar material 1	0.44	10.65	0.004
CTSS	NM_004079	cathepsin S	0.42	11.03	0.004
EIF3S9	NM_003751	eukaryotic translation initiation factor 3, subunit 9 eta, 116kDa	0.79	10.05	0.004
BASP1	IN_86		0.52	10.54	0.004
ADORA2A	NM_000675	adenosine A2a receptor	0.46	10.94	0.004
BRDT	NM_001726	bromodomain, testis-specific	0.45	10.49	0.004
MAPK6	IN_37		0.42	10.70	0.004
PSA	NM_021154	phosphoserine aminotransferase 1	0.59	10.90	0.004
TIMM13B	NM_012458	translocase of inner mitochondrial membrane 13 homolog (yeast)	0.50	11.36	0.004
SREC	NM_003693	scavenger receptor class F, member 1	0.44	10.66	0.004
GAS11	NM_001481	growth arrest-specific 8	0.46	10.77	0.004
PTPN1	NM_002827	protein tyrosine phosphatase, non-receptor type 1	0.63	10.47	0.004
TAF2B	NM_003184	TAF2 RNA polymerase II, TATA box binding protein (TBP)-associated factor, 150kDa	0.49	10.82	0.004
SCN4A	NM_000334	sodium channel, voltage-gated, type IV, alpha	0.44	10.57	0.004
SUOX	NM_000456	sulfite oxidase	0.36	10.41	0.004
MPHOSPH6	NM_005792	M-phase phosphoprotein 6	0.53	11.15	0.004
EIF3S4	NM_003755	eukaryotic translation initiation factor 3, subunit 4 delta, 44kDa	0.50	10.41	0.004
MC4R	NM_005912	melanocortin 4 receptor	0.40	10.43	0.004
CD2AP	NM_012120	CD2-associated protein	0.46	10.81	0.004
GOLTC1	NM_006348	component of oligomeric golgi complex 5	0.71	8.26	0.004
AMY	NM_014279	olfactomedin 1	0.54	10.48	0.004
RNF3	IN_83		0.48	9.42	0.005
SNRPG	IN_145		0.48	10.39	0.005
ASGR1	NM_001671	asialoglycoprotein receptor 1	0.56	10.40	0.005
CUGBP1	IN_100		0.61	10.38	0.005
KRTHA4	NM_021013	keratin, hair, acidic, 4	0.49	10.62	0.005
PEX7	NM_000288	peroxisomal biogenesis factor 7	0.44	10.72	0.005
DUOX1	NM_017434	dual oxidase 1	0.44	10.54	0.005
PRKCE	IN_14		0.46	10.52	0.005
MAGEA8	IN_10		0.45	10.64	0.005
KLRC3	NM_002261	killer cell lectin-like receptor subfamily C, member 3	0.48	10.64	0.005
PTPRU	IN_127		0.51	10.61	0.005
KCNJ3	IN_49		0.41	10.79	0.005
RNASE6	NM_005615	ribonuclease, RNase A family, k6	0.47	10.70	0.005
ANGPTL1	IN_100		0.38	10.56	0.005
RCD-8	IN_106		0.43	10.54	0.005
ODZ1	NM_014253	odz, odd Oz/ten-m homolog 1(Drosophila)	0.42	10.86	0.005
BLZF1	NM_003666	basic leucine zipper nuclear factor 1 (JEM-1)	0.37	10.56	0.005
B3GAT3	NM_012200	beta-1,3-glucuronyltransferase 3 (glucuronosyltransferase I)	0.39	10.91	0.005
KRTHA2	NM_002278	keratin, hair, acidic, 2	0.37	11.03	0.005
MTHFD1	NM_005956	methylenetetrahydrofolate dehydrogenase (NADP+ dependent), methenyltetrahydrofolate cyclohydrolase, formyltetrahydrofolate synthetase	0.56	10.80	0.005

LOC55830	NM_018446	glycosyltransferase AD-017	0.62	10.56	0.005
MGAT4A	IN_87		0.58	10.07	0.005
THRB	IN_57		0.51	10.68	0.005
CHGB	NM_001819	chromogranin B (secretogranin 1)	0.48	11.10	0.005
G10	NM_003910	maternal G10 transcript	0.59	9.95	0.005
DMD	NM_004006	dystrophin (muscular dystrophy, Duchenne and Becker types)	0.40	10.70	0.005
HTR1B	NM_000863	5-hydroxytryptamine (serotonin) receptor 1B	0.50	10.35	0.005
RAB35	NM_006861	RAB35, member RAS oncogene family	0.48	9.80	0.005
CCNK	IN_107		0.63	10.05	0.005
LIM2	NM_030657	lens intrinsic membrane protein 2, 19kDa	0.53	10.89	0.005
KCNJ1	NM_000220	potassium inwardly-rectifying channel, subfamily J, member 1	0.46	10.80	0.005
NDUFA2	NM_002488	NADH dehydrogenase (ubiquinone) 1 alpha subcomplex, 2, 8kDa	0.46	10.72	0.005
USP9Y	NM_004654	ubiquitin specific protease 9, Y-linked (fat facets-like, Drosophila)	0.36	10.68	0.005
BIN2	NM_016293	bridging integrator 2	0.39	10.74	0.005
BSG	NM_001728	basigin (OK blood group)	0.51	10.84	0.005
BTG3	NM_006806	BTG family, member 3	0.39	10.40	0.005
DRD5	NM_000798	dopamine receptor D5	0.45	10.77	0.005
TPSD1	NM_012217	tryptase delta 1	0.49	10.23	0.005
KCNE3	IN_65		0.56	11.06	0.005
MAB21L1	NM_005584	mab-21-like 1 (C. elegans)	0.56	10.11	0.005
LILRB3	IN_106		0.46	9.13	0.005
MAPKAPK5	NM_003668	mitogen-activated protein kinase-activated protein kinase 5	0.44	10.57	0.005
PGR	NM_000926	progesterone receptor	0.76	9.43	0.005
IL12RB1	NM_005535	interleukin 12 receptor, beta 1	0.58	10.66	0.005
PSMA5	NM_002790	proteasome (prosome, macropain) subunit, alpha type, 5	0.41	10.46	0.005
KLRD1	NM_002262	killer cell lectin-like receptor subfamily D, member 1	0.43	10.83	0.006
SSB	NM_003142	Sjogren syndrome antigen B (autoantigen La)	0.55	10.65	0.006
SECTM1	NM_003004	secreted and transmembrane 1	0.64	10.49	0.006
ARPC1B	NM_005720	actin related protein 2/3 complex, subunit 1B, 41kDa	0.42	10.68	0.006
ERCC4	NM_005236	excision repair cross-complementing rodent repair deficiency, complementation group 4	0.45	10.36	0.006
LOXL1	NM_005576	lysyl oxidase-like 1	0.57	10.46	0.006
ISGF3G	NM_006084	interferon-stimulated transcription factor 3, gamma 48kDa	0.64	8.83	0.006
F8	IN_10		0.64	10.40	0.006
SNRPB2	NM_003092	small nuclear ribonucleoprotein polypeptide B''	0.42	10.76	0.006
SCEL	NM_003843	sciellin	0.42	10.45	0.006
HAT1	UP_59		0.40	10.34	0.006
IGSF2	NM_004258	immunoglobulin superfamily, member 2	0.45	10.33	0.006
ARHGEF1	NM_004706	Rho guanine nucleotide exchange factor (GEF) 1	0.77	10.27	0.006
SEC63L	NM_007214	SEC63-like (S. cerevisiae)	0.48	10.18	0.006
NFX1	IN_49		0.54	11.02	0.006
PDHA1	NM_000284	pyruvate dehydrogenase (lipoamide) alpha 1	0.48	10.56	0.006
MCM3	NM_002388	MCM3 minichromosome maintenance deficient 3 (S. cerevisiae)	0.47	10.71	0.006
GTF3C5	NM_012087	general transcription factor IIIC, polypeptide 5, 63kDa	0.50	10.69	0.006
EIF4B	NM_001417	eukaryotic translation initiation factor 4B	0.52	10.30	0.006
KCNN2	NM_021614	potassium intermediate/small conductance calcium-activated channel, subfamily N, member 2	0.40	10.76	0.006
TRAF1	NM_005658	TNF receptor-associated factor 1	0.63	10.00	0.006
HIVEP1	NTF_4		0.59	10.48	0.006
PP35	NM_181581	protein similar to E.coli yhdg and R. capsulatus nifR3	0.61	9.39	0.006
D12S2489E	NM_007360	killer cell lectin-like receptor subfamily K, member 1	0.42	10.20	0.006
NUMA1	IN_16		0.42	10.94	0.006

LRP6	NM_002336	low density lipoprotein receptor-related protein 6	0.42	10.38	0.006
PDE9A	NM_002606	phosphodiesterase 9A	0.44	10.89	0.006
YES1	NM_005433	v-yes-1 Yamaguchi sarcoma viral oncogene homolog 1	0.35	11.04	0.006
RAB13	IN_16		0.45	10.53	0.006
TNRC11	NM_005120	trinucleotide repeat containing 11 (THR-associated protein, 230kDa subunit)	0.43	10.99	0.006
GAS7	NM_005890	growth arrest-specific 7	0.35	10.70	0.006
TGM2	NM_004613	transglutaminase 2 (C polypeptide, protein-glutamine-gamma-glutamyltransferase)	0.57	10.73	0.006
LTBR	NM_002342	lymphotoxin beta receptor (TNFR superfamily, member 3)	0.57	10.40	0.006
NMOR2	NM_000904	NAD(P)H dehydrogenase, quinone 2	0.43	11.24	0.006
LOC56891	NM_020129	lectin, galactoside-binding, soluble, 14	0.61	11.23	0.006
GPR17	NM_005291	G protein-coupled receptor 17	0.36	10.58	0.006
EVA1	NM_005797	epithelial V-like antigen 1	0.50	11.23	0.006
PON1	NM_000446	paraoxonase 1	0.52	10.46	0.006
DEFA5	NM_021010	defensin, alpha 5, Paneth cell-specific	0.39	10.53	0.007
GNPAT	NM_014236	glyceronephosphate O-acyltransferase	0.42	10.76	0.007
APEH	NM_001640	N-acylaminoacyl-peptide hydrolase	0.54	10.54	0.007
ASCL2	NM_005170	achaete-scute complex-like 2 (Drosophila)	0.41	10.54	0.007
FXYD1	NM_021902	FXYD domain containing ion transport regulator 1 (phospholemman)	0.42	10.35	0.007
COX7A1	NM_001864	cytochrome c oxidase subunit VIIa polypeptide 1 (muscle)	0.51	10.49	0.007
SFPQ	NM_005066	splicing factor proline/glutamine rich (polypyrimidine tract binding protein associated)	0.54	10.45	0.007
PIG3	NM_004881	tumor protein p53 inducible protein 3	0.38	10.81	0.007
MICB	NM_005931	MHC class I polypeptide-related sequence B	0.45	10.62	0.007
SLC25A14	NM_003951	solute carrier family 25 (mitochondrial carrier, brain), member 14	0.52	10.74	0.007
LOC51052	NM_015893	prolactin releasing hormone	0.39	10.73	0.007
LOC51148	NM_016174	cerebral endothelial cell adhesion molecule 1	0.42	11.07	0.007
CLN3	NM_000086	ceroid-lipofuscinosis, neuronal 3, juvenile (Batten, Spielmeyer-Vogt disease)	0.42	10.90	0.007
GJA3	NM_021954	gap junction protein, alpha 3, 46kDa (connexin 46)	0.47	10.98	0.007
NAB1	NM_005966	NGFI-A binding protein 1 (EGR1 binding protein 1)	0.44	10.41	0.007
LILRA2	NM_006866	leukocyte immunoglobulin-like receptor, subfamily A (with TM domain), member 2	0.85	10.19	0.007
MMP10	NM_002425	matrix metalloproteinase 10 (stromelysin 2)	0.39	10.73	0.007
MUTYH	NM_012222	mutY homolog (E. coli)	0.63	10.59	0.007
ALPI	NM_001631	alkaline phosphatase, intestinal	0.58	10.80	0.007
UGT2B11	UP_102		0.56	10.07	0.007
POU1F1	NM_000306	POU domain, class 1, transcription factor 1 (Pit1, growth hormone factor 1)	0.41	10.08	0.007
MLF2	NM_005439	myeloid leukemia factor 2	0.60	10.54	0.008
LTB	NM_002341	lymphotoxin beta (TNF superfamily, member 3)	0.56	10.00	0.008
SPOP	NM_003563	speckle-type POZ protein	0.34	10.55	0.008
RPLP1	NM_001003	ribosomal protein, large, P1	0.39	10.79	0.008
PIR51	NM_006479	RAD51-interacting protein	0.55	9.21	0.008
GLA	NM_000169	galactosidase, alpha	0.80	10.81	0.008
PLAUR	NM_002659	plasminogen activator, urokinase receptor	0.47	10.41	0.008
FY	NM_002036	Duffy blood group	0.40	10.50	0.008
A14GALT	IN_148		0.35	11.06	0.008
UNG2	NM_021147	uracil-DNA glycosylase 2	0.83	8.82	0.008
HDAC4	IN_119		0.50	10.95	0.008
SECRET	NM_006998	secretagogin, EF-hand calcium binding protein	0.74	8.89	0.008
RBM7	NM_016090	RNA binding motif protein 7	0.40	10.66	0.008
NR1D1	NM_021724	nuclear receptor subfamily 1, group D, member 1	0.49	10.24	0.008
SCYC1	NM_002995	chemokine (C motif) ligand 1	0.31	10.94	0.008
CCS	NM_005125	copper chaperone for superoxide dismutase	0.38	11.03	0.008

EZH2	IN_172		0.42	10.70	0.008
IMP-2	IN_56		0.41	10.31	0.008
LRN	NM_002319	leucine-rich repeats and calponin homology (CH) domain containing 4	0.74	11.10	0.008
RECQL	NM_002907	RecQ protein-like (DNA helicase Q1-like)	0.46	10.50	0.008
SHBG	NM_001040	sex hormone-binding globulin	0.44	10.68	0.008
MEP1B	NM_005925	meprin A, beta	0.48	10.78	0.008
HP1-BP74	NM_016287	HP1-BP74	0.46	10.40	0.008
COL3A1	NM_000090	collagen, type III, alpha 1 (Ehlers-Danlos syndrome type IV, autosomal dominant)	0.99	7.62	0.008
FOXD1	NM_004472	forkhead box D1	0.45	9.83	0.008
EVI2B	IN_27		0.36	10.81	0.008
GPR21	NM_005294	G protein-coupled receptor 21	0.52	10.92	0.008
HTN3	NM_000200	histatin 3	0.43	10.57	0.008
CYP3A5	NM_000777	cytochrome P450, family 3, subfamily A, polypeptide 5	0.80	10.43	0.008
NPAT	NM_002519	nuclear protein, ataxia-telangiectasia locus	0.59	10.63	0.008
HB-1	NM_021182	minor histocompatibility antigen HB-1	0.51	10.65	0.008
FNTB	NM_002028	farnesyltransferase, CAAX box, beta	0.50	10.82	0.008
PPP1CC	IN_30		0.50	10.59	0.009
MIPEP	NM_005932	mitochondrial intermediate peptidase	0.47	10.54	0.009
TLR9	NM_017442	toll-like receptor 9	0.39	10.80	0.009
GCHFR	NM_005258	GTP cyclohydrolase I feedback regulator	0.55	9.83	0.009
MUF1	IN_161		0.38	11.00	0.009
STAG3	NM_012447	stromal antigen 3	0.58	10.00	0.009
LTK	NM_002344	leukocyte tyrosine kinase	0.39	10.56	0.009
TIM44	NM_006351	translocase of inner mitochondrial membrane 44 homolog (yeast)	0.48	9.97	0.009
KIAA0851	NM_014016	SAC1 suppressor of actin mutations 1-like (yeast)	0.57	10.43	0.009
IGSF4	IN_71		0.38	10.64	0.009
OSRF	NM_012382	osmosis responsive factor	0.48	10.33	0.009
MAGEA12	IN_24		0.36	10.84	0.009
TACR2	NM_001057	tachykinin receptor 2	0.59	10.69	0.009
GP1BA	NM_000173	glycoprotein Ib (platelet), alpha polypeptide	0.59	10.49	0.009
H3FI	NM_021018	histone 1, H3f	0.48	10.72	0.009
CCT7	NM_006429	chaperonin containing TCP1, subunit 7 (eta)	0.67	10.16	0.009
H3FL	NM_003537	histone 1, H3b	0.42	9.73	0.009
HSPD1	UP_36		0.43	10.49	0.009
PAX1	NM_006192	paired box gene 1	0.43	10.69	0.009
PRAX-1	NM_004758	benzodiazapine receptor (peripheral) associated protein 1	0.46	10.78	0.009
GALK2	NM_002044	galactokinase 2	0.43	11.04	0.010
FMO5	NM_001461	flavin containing monooxygenase 5	0.58	10.38	0.010
GRID2	IN_53		0.51	10.92	0.010
MTRR	NM_024010	5-methyltetrahydrofolate-homocysteine methyltransferase reductase	0.52	10.74	0.010
IL10RB	NM_000628	interleukin 10 receptor, beta	0.43	10.58	0.010
LZLP	NM_013344		0.37	10.81	0.010
SRP9	NM_003133	signal recognition particle 9kDa	0.36	10.68	0.010
C8B	NM_000066	complement component 8, beta polypeptide	0.50	10.41	0.010
HIF1A	NM_001530	hypoxia-inducible factor 1, alpha subunit (basic helix-loop-helix transcription factor)	0.45	10.29	0.010
UPK2	NM_006760	uroplakin 2	0.60	9.66	0.010
SGK2	NM_170693	serum/glucocorticoid regulated kinase 2	0.37	10.63	0.010
NEU1	NM_000434	sialidase 1 (lysosomal sialidase)	0.41	10.85	0.010
PDK1	NM_002610	pyruvate dehydrogenase kinase, isoenzyme 1	0.62	8.57	0.010
UGTREL1	NM_005827	solute carrier family 35, member B1	0.73	8.42	0.010

PXF	NM_002857	peroxisomal biogenesis factor 19	0.41	10.27	0.010
IFNA14	NM_002172	interferon, alpha 14	0.66	10.21	0.010
KIAA0623	NM_014683	unc-51-like kinase 2 (C. elegans)	0.40	10.80	0.010
JAK1	NM_002227	Janus kinase 1 (a protein tyrosine kinase)	0.42	10.61	0.011
POLR2K	NM_005034	polymerase (RNA) II (DNA directed) polypeptide K, 7.0kDa	0.32	10.95	0.011
ORC2L	NM_006190	origin recognition complex, subunit 2-like (yeast)	0.48	10.58	0.011
SFRS1	NM_006924	splicing factor, arginine/serine-rich 1 (splicing factor 2, alternate splicing factor)	0.38	10.16	0.011
CRHR1	IN_129		0.52	11.05	0.011
SARS	NM_006513	seryl-tRNA synthetase	0.39	11.04	0.011
CD5	NTF_17		0.43	10.51	0.011
PC4	UP_100		0.64	9.03	0.011
CKM	NM_001824	creatine kinase, muscle	0.54	9.27	0.011
MAPRE2	NM_014268	microtubule-associated protein, RP/EB family, member 2	0.71	8.71	0.011
STX6	NM_005819	syntaxin 6	0.42	11.00	0.011
MID1	IN_124		0.40	10.94	0.011
PIN1L	NM_006222	protein (peptidyl-prolyl cis/trans isomerase) NIMA-interacting 1-like	0.96	8.70	0.011
SLC22A4	NM_003059	solute carrier family 22 (organic cation transporter), member 4	0.48	8.94	0.011
KIAA0132	NM_012289	kelch-like ECH-associated protein 1	0.51	10.78	0.011
PLG	NM_000301	plasminogen	0.40	10.25	0.011
M6PR	NM_002355	mannose-6-phosphate receptor (cation dependent)	0.35	10.46	0.011
PPP2R4	NM_021131	protein phosphatase 2A, regulatory subunit B' (PR 53)	0.49	10.31	0.011
GNLY	NM_006433	granulysin	0.43	10.80	0.011
MTA1L1	NM_004739	metastais-associated gene family, member 2	0.33	10.85	0.011
ZP2	NM_003460	zona pellucida glycoprotein 2 (sperm receptor)	0.37	10.33	0.011
PAK4	NM_005884	p21(CDKN1A)-activated kinase 4	0.42	9.98	0.011
NDUFA8	NM_014222	NADH dehydrogenase (ubiquinone) 1 alpha subcomplex, 8, 19kDa	0.34	10.44	0.011
PEGASUS	NM_022466	zinc finger protein, subfamily 1A, 5	0.39	10.80	0.012
TERF2	NM_005652	telomeric repeat binding factor 2	0.42	11.01	0.012
IL1RAPL1	IN_113		0.74	8.40	0.012
MEF2A	NM_005587	MADS box transcription enhancer factor 2, polypeptide A (myocyte enhancer factor 2A)	0.39	10.94	0.012
HBS1L	NM_006620	HBS1-like (S. cerevisiae)	0.36	10.52	0.012
EPB49	NM_001978	erythrocyte membrane protein band 4.9 (dematin)	0.54	10.09	0.012
POLI	NM_007195	polymerase (DNA directed) iota	0.43	10.31	0.012
HMBS	NM_000190	hydroxymethylbilane synthase	0.42	10.55	0.012
RXRG	NM_006917	retinoid X receptor, gamma	0.38	10.58	0.012
TIMM8B	NM_012459	translocase of inner mitochondrial membrane 8 homolog B (yeast)	0.43	10.47	0.012
TSC2	NM_000548	tuberous sclerosis 2	0.34	11.01	0.012
GAD1	NM_000817	glutamate decarboxylase 1 (brain, 67kDa)	0.43	10.48	0.012
TSA1902	IN_109		0.38	10.82	0.013
MTX2	NM_006554	metaxin 2	0.32	8.99	0.013
IGFBP5	NM_000599	insulin-like growth factor binding protein 5	0.44	10.58	0.013
SOX20	NM_006942	SRY (sex determining region Y)-box 15	0.46	10.16	0.013
CXCR4	IN_118		0.37	10.29	0.013
ARHGDIB	NM_001175	Rho GDP dissociation inhibitor (GDI) beta	0.45	10.72	0.013
PSMC5	NM_002805	proteasome (prosome, macropain) 26S subunit, ATPase, 5	0.40	9.69	0.013
MAP1A	IN_9		0.45	10.67	0.013
KCNMB1	NM_004137	potassium large conductance calcium-activated channel, subfamily M, beta member 1	0.34	10.93	0.013
GALR3	NM_003614	galanin receptor 3	0.40	10.34	0.013
BRD3	IN_118		0.58	9.49	0.013
KLKB1	NM_000892	kallikrein B, plasma (Fletcher factor) 1	0.40	10.74	0.013

GLP2R	NM_004246	glucagon-like peptide 2 receptor	0.34	10.85	0.013
NLP_1	NM_007342	nucleoporin like 2	0.65	8.48	0.013
IL1B	NTF_169		0.33	10.56	0.014
TSSC3	NM_003311	pleckstrin homology-like domain, family A, member 2	0.49	10.67	0.014
SMARCF1	IN_103		0.47	9.93	0.014
E2F1:500	NM_005225	E2F transcription factor 1	0.42	10.40	0.014
PFKL	NM_002626	phosphofructokinase, liver	0.35	10.29	0.014
GAS41	NM_006530	glioma-amplified sequence-41	0.42	10.60	0.014
LIFR	NM_002310	leukemia inhibitory factor receptor	0.51	10.81	0.014
NME2	NM_002512	non-metastatic cells 2, protein (NM23B) expressed in	0.42	11.04	0.014
DNAI1	NM_012144	dynein, axonemal, intermediate polypeptide 1	0.34	10.73	0.014
SF3A1	NM_005877	splicing factor 3a, subunit 1, 120kDa	0.75	8.25	0.014
HOXC11	NM_014212	homeo box C11	0.32	10.64	0.014
HPS	NM_000195	Hermansky-Pudlak syndrome 1	0.39	10.72	0.014
RAGA	NM_006570	Ras-related GTP binding A	0.70	10.71	0.014
GNGT1	NM_021955	guanine nucleotide binding protein (G protein), gamma transducing activity polypeptide 1	0.42	10.61	0.014
PITX3	IN_6		0.37	10.81	0.014
KCNB1	NM_004975	potassium voltage-gated channel, Shab-related subfamily, member 1	0.36	10.92	0.014
HSPE1	NM_002157	heat shock 10kDa protein 1 (chaperonin 10)	0.36	10.73	0.014
KLRA1	NM_006611	killer cell lectin-like receptor subfamily A, member 1	1.23	8.19	0.014
RPS25	NM_001028	ribosomal protein S25	0.47	9.95	0.015
EBP	NM_006579	emopamil binding protein (sterol isomerase)	0.41	9.89	0.015
SCAD-SRL	NM_021004	dehydrogenase/reductase (SDR family) member 4	0.80	8.75	0.015
PTCH2	IN_116		0.51	10.44	0.015
GNAT1	NM_000172	guanine nucleotide binding protein (G protein), alpha transducing activity polypeptide 1	0.45	10.26	0.015
C8FW	NM_025195	tribbles homolog 1 (Drosophila)	0.32	11.01	0.015
PRSS12	IN_124		0.31	10.73	0.015
INHA	NM_002191	inhibin, alpha	0.36	10.68	0.015
COL13A1	NM_005203	collagen, type XIII, alpha 1	0.37	10.31	0.015
POLR2F	NM_021974	polymerase (RNA) II (DNA directed) polypeptide F	0.44	10.51	0.015
GKAP42	NM_025211	G kinase anchoring protein 1	0.43	10.68	0.015
MAGEA5	IN_4		0.51	11.07	0.016
AHCYL1	NM_006621	S-adenosylhomocysteine hydrolase-like 1	0.52	8.73	0.016
CRHR2	NM_001883	corticotropin releasing hormone receptor 2	0.48	10.64	0.016
ALDH9A1	NM_000696	aldehyde dehydrogenase 9 family, member A1	0.36	10.91	0.016
DDX5	NM_004396	DEAD (Asp-Glu-Ala-Asp) box polypeptide 5	0.36	10.29	0.016
SNAPC2	NM_003083	small nuclear RNA activating complex, polypeptide 2, 45kDa	0.54	8.70	0.016
APOC4	NM_001646	apolipoprotein C-IV	0.38	10.49	0.016
CRYBA1	NM_005208	crystallin, beta A1	0.47	10.07	0.016
AGTR2	NM_000686	angiotensin II receptor, type 2	0.47	10.87	0.016
GAS11	NM_001481	growth arrest-specific 8	0.33	10.81	0.016
NBR2	NM_005821	neighbor of BRCA1 gene 2	0.52	9.50	0.016
MADH4	IN_0		0.31	10.48	0.016
PPT1	NM_000310	palmitoyl-protein thioesterase 1 (ceroid-lipofuscinosis, neuronal 1, infantile)	0.56	10.38	0.017
CYB5-M	NM_030579	cytochrome b5 outer mitochondrial membrane precursor	0.53	10.20	0.017
ASH2L	NM_004674	ash2 (absent, small, or homeotic)-like (Drosophila)	0.60	10.30	0.017
FXC1	NM_012192	fracture callus 1 homolog (rat)	0.34	10.65	0.017
RalGPS1A	NM_014636		0.40	10.58	0.017
SERPINI2	IN_11		0.37	10.30	0.017
PNMA1	NM_006029	paraneoplastic antigen MA1	0.32	10.72	0.017

H4FA	NM_003538	histone 1, H4a	0.69	10.06	0.017
RPL26	NM_000987	ribosomal protein L26	0.33	10.83	0.017
HEAB	NM_006831	ATP/GTP-binding protein	0.35	10.43	0.017
TNS	UP_106		0.52	10.54	0.017
CLDN5	NM_003277	claudin 5 (transmembrane protein deleted in velocardiofacial syndrome)	0.38	11.11	0.017
ARL1	NM_001177	ADP-ribosylation factor-like 1	0.38	10.79	0.017
IL7	NM_000880	interleukin 7	0.36	10.37	0.018
IL13RA2	NM_000640	interleukin 13 receptor, alpha 2	0.35	10.56	0.018
REPS2	IN_95		0.43	10.74	0.018
FPRL2	NM_002030	formyl peptide receptor-like 2	0.30	10.41	0.018
MANBA	NM_005908	mannosidase, beta A, lysosomal	0.40	10.30	0.018
LOC51065	NM_015920	ribosomal protein S27-like	0.31	10.45	0.018
LTF:376	UP_23		0.37	10.31	0.018
HCGIX	NM_005844	HLA complex group 9	0.49	10.01	0.018
PLA2G1B	NM_000928	phospholipase A2, group IB (pancreas)	0.33	10.24	0.018
SERPINA7	NM_000354	serine (or cysteine) proteinase inhibitor, clade A (alpha-1 antiproteinase, antitrypsin), member 7	0.28	10.54	0.018
DRD3	IN_53		0.43	10.91	0.019
RBMY2B	UP_43		0.65	10.25	0.019
BCAS1	NM_003657	breast carcinoma amplified sequence 1	0.29	11.12	0.019
PEPD	NM_000285	peptidase D	0.39	10.58	0.019
HSF2BP	NM_007031	heat shock transcription factor 2 binding protein	0.48	8.61	0.019
DLX3	NM_005220	distal-less homeo box 3	0.31	10.84	0.019
RNASE3L	IN_54		0.51	10.59	0.019
MBD3	NM_003926	methyl-CpG binding domain protein 3	0.34	10.88	0.019
JWA	NM_006407	cytoskeleton related vitamin A responsive protein	0.52	9.08	0.019
NCAM2	IN_20		0.48	10.25	0.019
PLS3	NM_005032	plastin 3 (T isoform)	0.30	10.45	0.019
HMG1	NM_002128	high-mobility group box 1	0.54	10.60	0.019
SNK	NM_006622	polo-like kinase 2 (Drosophila)	0.52	9.76	0.019
ZFP95	NM_145102	zinc finger protein 95 homolog (mouse)	0.87	8.05	0.020
HS3ST3B1	NM_006041	heparan sulfate (glucosamine) 3-O-sulfotransferase 3B1	0.48	10.73	0.020
CYB5	NM_001914	cytochrome b-5	0.34	10.51	0.020
M9	NM_013234	eukaryotic translation initiation factor 3 subunit k	0.47	10.55	0.020
NDUFB4	NM_004547	NADH dehydrogenase (ubiquinone) 1 beta subcomplex, 4, 15kDa	0.36	10.39	0.020
H2BFA	NM_003518	histone 1, H2bg	0.43	10.49	0.020
G6C	NM_025261	lymphocyte antigen 6 complex, locus G6C	0.30	10.66	0.020
KCNJ11	NM_000525	potassium inwardly-rectifying channel, subfamily J, member 11	0.30	10.62	0.020
MMP7	NM_002423	matrix metalloproteinase 7 (matrilysin, uterine)	0.48	10.88	0.020
UBE4A	NM_004788	ubiquitination factor E4A (UFD2 homolog, yeast)	0.43	10.96	0.021
PDGFRL	NM_006207	platelet-derived growth factor receptor-like	0.38	10.34	0.021
UBA52	NM_003333	ubiquitin A-52 residue ribosomal protein fusion product 1	0.38	10.45	0.021
MEL	NM_005370	RAB8A, member RAS oncogene family	0.30	10.53	0.021
HTR1D	NM_000864	5-hydroxytryptamine (serotonin) receptor 1D	0.36	10.68	0.021
KIR3DL1	IN_16		0.45	10.54	0.021
DIM1	NM_006701	thioredoxin-like 4	0.53	10.08	0.021
VELI1	NM_004664	lin-7 homolog A (C. elegans)	0.37	10.09	0.021
GLO1	NM_006708	glyoxalase I	0.30	10.95	0.021
CCR2	NM_000647	chemokine (C-C motif) receptor 2	0.55	9.69	0.022
MAPT	NM_005910	microtubule-associated protein tau	0.41	10.99	0.022
CYLC2	IN_37		0.51	10.19	0.022

ILVBL	NM_006844	ilvB (bacterial acetolactate synthase)-like	0.43	10.54	0.022
PDE6D	NM_002601	phosphodiesterase 6D, cGMP-specific, rod, delta	0.69	10.32	0.022
FACL3	NM_004457	acyl-CoA synthetase long-chain family member 3	0.41	10.15	0.022
ZNF74	NM_003426	zinc finger protein 74 (Cos52)	0.47	10.94	0.022
PCYT1A	NM_005017	phosphate cytidylyltransferase 1, choline, alpha isoform	0.62	8.66	0.022
BCKDK	NM_005881	branched chain alpha-ketoacid dehydrogenase kinase	0.35	10.85	0.022
AMSH	IN_80		0.50	8.79	0.022
COL16A1	NM_001856	collagen, type XVI, alpha 1	0.40	10.75	0.022
TNFSF8	NM_001244	tumor necrosis factor (ligand) superfamily, member 8	0.40	11.05	0.022
GMPS	IN_86		0.41	10.54	0.022
OVOL1	NM_004561	ovo-like 1(Drosophila)	0.31	10.62	0.023
MYD88	NM_002468	myeloid differentiation primary response gene (88)	0.30	10.49	0.023
PNMA2	NM_007257		0.57	10.29	0.023
FMO1	NM_002021	flavin containing monooxygenase 1	0.50	10.25	0.023
OAS1	NM_016816	2',5'-oligoadenylate synthetase 1, 40/46kDa	0.54	10.50	0.023
LOC51172	NM_016256	N-acetylglucosamine-1-phosphodiester alpha-N-acetylglucosaminidase	0.29	10.69	0.023
IL6	NM_000600	interleukin 6 (interferon, beta 2)	0.47	10.09	0.023
RAGB	NM_006064	Ras-related GTP binding B	0.39	10.38	0.023
LOC51337	NM_016647	mesenchymal stem cell protein DSCD75	0.52	11.23	0.023
LUM	NM_002345	lumican	0.52	10.77	0.023
MAP3K4	NM_005922	mitogen-activated protein kinase kinase kinase 4	0.40	10.40	0.024
NDUFV1	NM_007103	NADH dehydrogenase (ubiquinone) flavoprotein 1, 51kDa	0.40	10.33	0.024
LOC51167	IN_76		0.34	10.81	0.024
TSPAN-2	IN_55		0.29	10.62	0.024
CPLX2	NM_006650	complexin 2	0.96	8.10	0.024
HFE	NM_000410	hemochromatosis	0.27	10.90	0.024
SEMA3E	NM_012431		0.42	10.39	0.025
HUG1	NM_020527		0.39	8.74	0.025
MFGE8	NM_005928	milk fat globule-EGF factor 8 protein	0.34	10.40	0.025
RBBP8	NTF_22		0.32	11.04	0.025
SLIT1	NM_003061	slit homolog 1 (Drosophila)	0.42	8.39	0.025
RALY	NM_007367	RNA binding protein (autoantigenic, hnRNP-associated with lethal yellow)	0.44	10.07	0.025
PLP1	IN_11		0.29	10.39	0.025
ARG2	NM_001172	arginase, type II	0.40	10.97	0.025
ENTPD2	NM_001246	ectonucleoside triphosphate diphosphohydrolase 2	0.29	10.72	0.025
FBXO9	NM_012347	F-box protein 9	0.28	11.08	0.026
HMMR	NM_012484	hyaluronan-mediated motility receptor (RHAMM)	0.30	10.85	0.026
RPL22	IN_4		0.48	10.04	0.026
PNKP	NM_007254	polynucleotide kinase 3'-phosphatase	0.53	10.26	0.026
SNT-1	IN_91		0.52	8.95	0.026
PRB4	NM_002723	proline-rich protein BstNI subfamily 4	0.50	9.94	0.026
CTSL2	NM_001333	cathepsin L2	0.42	11.11	0.026
TIM44	NM_006351	translocase of inner mitochondrial membrane 44 homolog (yeast)	0.36	10.55	0.027
PPARG	NM_015869	peroxisome proliferative activated receptor, gamma	0.30	10.53	0.027
APCL	NM_005883	adenomatosis polyposis coli 2	0.45	8.32	0.027
WDR4	NM_033661	WD repeat domain 4	0.67	7.85	0.027
KRTHB1	NM_002281	keratin, hair, basic, 1	0.48	10.74	0.028
HSPB1	NM_001540	heat shock 27kDa protein 1	0.49	10.26	0.028
DUSP11	NM_003584	dual specificity phosphatase 11 (RNA/RNP complex 1-interacting)	0.32	10.28	0.028
REGL	NM_006508	regenerating islet-derived-like, pancreatic stone protein-like, pancreatic thread protein-like (rat)	0.46	10.90	0.028

GENX-3414	NM_003943	genethonin 1	0.52	10.61	0.028
TLR3	NM_003265	toll-like receptor 3	0.31	11.21	0.028
RAC2	NM_002872	ras-related C3 botulinum toxin substrate 2 (rho family, small GTP binding protein Rac2)	0.34	10.63	0.029
LOC51042	NM_015871	zinc finger protein 593	0.30	10.90	0.029
LPRP	NM_007244	proline rich 4 (lacrimal)	0.36	10.39	0.029
UGT2B4	NM_021139	UDP glycosyltransferase 2 family, polypeptide B4	0.56	10.25	0.029
PELO	IN_148		0.26	10.53	0.029
UXT	NM_004182	ubiquitously-expressed transcript	0.36	10.83	0.029
CPT1A	IN_169		0.74	8.26	0.029
ABCA8	IN_76		0.33	7.96	0.029
SEDL	NM_014563	spondyloepiphyseal dysplasia, late	0.29	11.14	0.030
PKDREJ	NM_006071	polycystic kidney disease (polycystin) and REJ (sperm receptor for egg jelly homolog, sea urchin)-like	0.54	8.84	0.030
DNAJA1	NM_001539	DnaJ (Hsp40) homolog, subfamily A, member 1	0.34	10.90	0.030
ME3	IN_96		0.44	9.57	0.030
PLCG1	NM_002660	phospholipase C, gamma 1	0.34	10.54	0.030
NR3C1	IN_22		0.30	10.77	0.030
NKX6A	NM_006168	NK6 transcription factor related, locus 1 (Drosophila)	0.35	10.93	0.030
BID	NM_197967	BH3 interacting domain death agonist	0.31	10.80	0.030
HSXIAPAF1	NM_017523	XIAP associated factor-1	0.32	10.53	0.031
GPD2	IN_41		0.30	10.45	0.031
LOC51292	NM_016576	guanosine monophosphate reductase 2	0.32	10.50	0.031
NSAP1	UP_108		0.69	8.54	0.031
HSD17B1	NM_000413	hydroxysteroid (17-beta) dehydrogenase 1	0.35	10.63	0.031
CDH17	NM_004063	cadherin 17, LI cadherin (liver-intestine)	0.35	11.11	0.031
PSPN	NM_004158	persephin	0.29	10.65	0.031
ALDOA	NM_184043	aldolase A, fructose-bisphosphate	0.41	10.48	0.031
IFI27	NM_005532	interferon, alpha-inducible protein 27	0.55	10.29	0.032
CLDN10	NM_006984	claudin 10	0.77	10.78	0.032
ATP2C1	NM_014382	ATPase, Ca++ transporting, type 2C, member 1	0.27	10.65	0.032
PITX1	IN_11		0.30	10.71	0.032
MRAS	UP_79		0.34	8.79	0.032
UBE2E1	NM_003341	ubiquitin-conjugating enzyme E2E 1 (UBC4/5 homolog, yeast)	0.38	10.48	0.032
RCN1	NM_002901	reticulocalbin 1, EF-hand calcium binding domain	0.46	9.97	0.032
LOC51191	NM_016323	hect domain and RLD 5	0.25	10.91	0.032
PRKCABP	NM_012407	protein kinase C, alpha binding protein	0.93	8.31	0.033
KLK2	NM_005551	kallikrein 2, prostatic	0.37	10.49	0.033
LOC54466	NM_019003	spindlin family, member 2	0.34	11.09	0.033
ALAS1	NM_000688	aminolevulinate, delta-, synthase 1	0.29	10.66	0.033
SLAM	NM_003037	signaling lymphocytic activation molecule family member 1	0.40	10.55	0.033
TARBP1	NM_005646	TAR (HIV) RNA binding protein 1	0.30	10.82	0.033
WIF-1	IN_117		0.42	10.48	0.034
RABL2A	NM_007082	RAB, member of RAS oncogene family-like 2A	0.40	10.32	0.034
PMS2L8	NM_013440	paired immunoglobin-like type 2 receptor beta	0.36	10.41	0.034
CEACAM1	NM_001712	carcinoembryonic antigen-related cell adhesion molecule 1 (biliary glycoprotein)	0.38	10.63	0.034
PKNOX1	IN_6		0.30	10.76	0.034
TCF12	NM_003205	transcription factor 12 (HTF4, helix-loop-helix transcription factors 4)	0.56	10.11	0.034
CD1C	NM_001765	CD1C antigen, c polypeptide	0.34	9.45	0.034
HOXD3	IN_175		0.40	10.29	0.035
JPH3	IN_171		0.30	10.94	0.035
IL10	NM_000572	interleukin 10	0.27	10.37	0.035

MGB1	NM_002411	secretoglobin, family 2A, member 2	0.39	10.26	0.036
ARHGAP6	NM_001174	Rho GTPase activating protein 6	0.42	10.71	0.036
DELGEF	NM_012139	deafness locus associated putative guanine nucleotide exchange factor	0.39	10.53	0.036
GIF	NM_005142	gastric intrinsic factor (vitamin B synthesis)	0.26	10.52	0.036
FLJ22170	NM_025099		0.27	10.78	0.036
HLA-B	NTF_30		0.40	10.47	0.036
CKS1	UP_159		0.40	9.82	0.036
C2	NM_000063	complement component 2	0.34	10.72	0.036
TDG	NM_003211	thymine-DNA glycosylase	0.35	10.54	0.037
GNE	NM_005476	glucosamine (UDP-N-acetyl)-2-epimerase/N-acetylmannosamine kinase	0.26	10.61	0.037
NDUFA1	NM_004541	NADH dehydrogenase (ubiquinone) 1 alpha subcomplex, 1, 7.5kDa	0.35	10.71	0.037
CS	NM_004077	citrate synthase	0.34	10.44	0.037
KRTHA3A	NM_004138	keratin, hair, acidic, 3A	0.43	10.47	0.038
TFAP2B	NM_003221	transcription factor AP-2 beta (activating enhancer binding protein 2 beta)	0.27	10.62	0.038
GADD45G	NM_006705	growth arrest and DNA-damage-inducible, gamma	0.26	10.89	0.038
TRFP	NM_004053	bystin-like	0.43	10.63	0.039
TK2	NM_004614	thymidine kinase 2, mitochondrial	0.37	10.24	0.039
HSPA1A	NM_005345	heat shock 70kDa protein 1A	0.32	10.73	0.039
CLPX	NM_006660	ClpX caseinolytic protease X homolog (E. coli)	0.45	9.30	0.039
GEMIN4	NM_015721	gem (nuclear organelle) associated protein 4	0.49	10.36	0.039
LY64	NM_005582	lymphocyte antigen 64 homolog, radioprotective 105kDa (mouse)	0.24	10.75	0.039
POLM	NM_013284	polymerase (DNA directed), mu	0.26	10.63	0.039
TFRC	NM_003234	transferrin receptor (p90, CD71)	0.32	10.41	0.040
TUBG1	NM_001070	tubulin, gamma 1	0.32	11.07	0.040
GRB14	IN_41		0.29	10.62	0.040
PRDX5	NM_012094	peroxiredoxin 5	0.29	10.24	0.040
HS3ST2	NM_006043	heparan sulfate (glucosamine) 3-O-sulfotransferase 2	0.44	10.89	0.040
TGM1	NM_000359	transglutaminase 1 (K polypeptide epidermal type I, protein-glutamine-gamma-glutamyltransferase)	0.31	10.57	0.040
LOC51159	NM_016206	colon carcinoma related protein	0.32	10.96	0.040
ZNF216	NM_006007	zinc finger protein 216	0.31	10.77	0.041
FLJ21079	IN_120		0.28	10.69	0.041
ESR2	NM_001437	estrogen receptor 2 (ER beta)	0.49	10.51	0.041
DIAPH1	NM_005219	diaphanous homolog 1 (Drosophila)	0.34	10.74	0.041
HOXA1	NM_005522	homeo box A1	0.47	10.35	0.041
FLJ23311	IN_122		0.38	10.56	0.041
CLECSF2	IN_57		0.25	10.63	0.041
BLCAP	NM_006698	bladder cancer associated protein	0.51	9.89	0.041
C4ORF6	NM_005750	chromosome 4 open reading frame 6	0.36	10.94	0.041
BCL10	NM_003921	B-cell CLL/lymphoma 10	0.36	10.14	0.041
SLC6A6	NM_003043	solute carrier family 6 (neurotransmitter transporter, taurine), member 6	0.31	10.38	0.042
TAS2R14	NM_023922	taste receptor, type 2, member 14	0.27	10.85	0.042
COL2A1	NM_001844	collagen, type II, alpha 1 (primary osteoarthritis, spondyloepiphyseal dysplasia, congenital)	0.33	10.72	0.042
NDUFS3	NM_004551	NADH dehydrogenase (ubiquinone) Fe-S protein 3, 30kDa (NADH-coenzyme Q reductase)	0.31	11.02	0.042
IMP-2	IN_56		0.34	9.65	0.042
FABP5	IN_33		0.31	10.61	0.042
CRP	NM_000567	C-reactive protein, pentraxin-related	0.31	10.81	0.043
STAU	NM_004602	staufen, RNA binding protein (Drosophila)	0.37	10.53	0.043
MKI67	NM_002417	antigen identified by monoclonal antibody Ki-67	0.58	8.66	0.043
CDR1	NM_004065	cerebellar degeneration-related protein 1, 34kDa	0.40	10.51	0.043
TPD52	NM_005079	tumor protein D52	0.41	10.48	0.043

ITPKB	NM_002221	inositol 1,4,5-trisphosphate 3-kinase B	0.32	10.80	0.043
SNAPC1	NM_003082	small nuclear RNA activating complex, polypeptide 1, 43kDa	0.27	10.62	0.044
SCYA21	NM_002989	chemokine (C-C motif) ligand 21	0.37	10.73	0.044
HLA-E	NM_005516	major histocompatibility complex, class I, E	0.30	10.81	0.044
FMO4	NM_002022	flavin containing monooxygenase 4	0.37	9.92	0.045
CENPF	IN_151		0.33	10.33	0.045
ATRX	UP_127		0.36	10.88	0.045
PRKDC	NM_006904	protein kinase, DNA-activated, catalytic polypeptide	0.31	10.39	0.046
GPR72	NM_016540	G protein-coupled receptor 83	0.34	9.41	0.046
PPIL2	NM_148176	peptidylprolyl isomerase (cyclophilin)-like 2	0.30	10.38	0.046
MNPEP	NM_006838	methionyl aminopeptidase 2	0.58	8.27	0.046
ZNF222	NM_013360	zinc finger protein 222	0.35	10.59	0.046
PTGER1	NM_000955	prostaglandin E receptor 1 (subtype EP1), 42kDa	0.44	9.81	0.047
RBMX	UP_113		0.30	10.42	0.047
PPP1R10	NM_002714	protein phosphatase 1, regulatory subunit 10	0.49	10.04	0.047
PDE1C	IN_33		0.47	10.35	0.047
CCNG2	NM_004354	cyclin G2	0.32	10.94	0.047
BM-002	NM_016617	ubiquitin-fold modifier 1	0.46	7.75	0.048
NOX5	IN_132		0.25	10.74	0.048
GPLD1	IN_52		0.32	11.02	0.048
SCYA19	NM_006274	chemokine (C-C motif) ligand 19	0.40	10.15	0.049
MYOM1	IN_165		0.31	10.55	0.049
YWHAG	IN_150		0.36	10.70	0.049
ELF3	IN_0		0.42	10.72	0.049
ATP6S1	IN_40		0.29	10.64	0.049
PPP4C	NM_002720	protein phosphatase 4 (formerly X), catalytic subunit	0.30	10.49	0.050
CH25H	NM_003956	cholesterol 25-hydroxylase	0.30	10.37	0.050
OS-9	NM_006812	amplified in osteosarcoma	0.40	9.70	0.050
