# Supplementary material for: Protein-Binding Microarray Analysis of Tumor Suppressor AP2α Target Gene Specificity
Source: PLoS One. 2011 Aug 18;6(8):e22895. doi: 10.1371/journal.pone.0022895 (PMC3158074; doi:10.1371/journal.pone.0022895)
Supplement: Table S6 — Analysis of the first 50 sequences found to be bound by AP2α in recombinant AP2 extract and in normal and cancer tissues extracts. Scores were defined by Weight Matrix Prediction Algorithms for AP2α and p53. For AP2α, scores <30 were defined as low binding sites. For p53, scores <50 were defined as low binding sites. (PDF) [file pone.0022895.s011.pdf]

Table S6. Occurrence of predicted AP2 and p53 binding sites in target genes of AP2 from various sources

Protein extract probed	% genes with AP2 weight matrix score >30	% genes with p53 weight matrix score >75	% genes with p53 and AP2 weight matrix score >75 and <30, respectively
Genes bound by recombinant AP2α	55	30	14
Genes bound using normal tissue extracts	35	27	18
Genes bound using cancer tissue extracts	63	30	13

Position weight matrix scores of >30 and of >75 were interpreted as indicative of a binding site for AP2α and p53, respectively.
